# The NICU Antibiotics and Outcomes (NANO) trial: a randomized multicenter clinical trial assessing empiric antibiotics and clinical outcomes in newborn preterm infants

**DOI:** 10.1186/s13063-022-06352-3

**Published:** 2022-05-23

**Authors:** Michael J. Morowitz, Anup C. Katheria, Richard A. Polin, Elizabeth Pace, David T. Huang, Chung-Chou H. Chang, Johathan G. Yabes

**Affiliations:** 1 Division of Pediatric General and Thoracic Surgery, University of Pittsburgh School of Medicine, Children’s Hospital of Pittsburgh of UPMC, Rangos Research Center 6th Floor, 4401 Penn Avenue, Pittsburgh, PA 15224 USA; 2 Division of Pediatrics, Sharp Mary Birch Hospital for Women & Newborns, San Diego, CA 92123 USA; 3 Department of Pediatrics, Columbia University, New York, NY 10032 USA; 4 Department of Surgery, University of Pittsburgh School of Medicine, Pittsburgh, USA; 5 Department of Critical Care Medicine, University of Pittsburgh, Pittsburgh, USA; 6 Department of General Internal Medicine, University of Pittsburgh, Pittsburgh, USA

**Keywords:** Microbial colonization, Extremely-low-birthweight, Prematurity, Early-onset neonatal sepsis, Late-onset neonatal sepsis, Necrotizing enterocolitis, Morbidity, Mortality

## Abstract

**Background:**

Early-onset sepsis is an important cause of neonatal morbidity and mortality in the preterm population. Infants perceived to be at increased risk for early-onset sepsis are often treated empirically with broad-spectrum antibiotics while awaiting confirmatory blood cultures, despite an overall incidence of early-onset sepsis of 2–3% among extremely-low-birthweight (ELBW) infants. Recent observational studies associate perinatal antibiotic use with an increased incidence of necrotizing enterocolitis, late-onset sepsis, and mortality among ELBW infants. Given currently available data and variability in clinical practice, we designed a prospective multi-institutional randomized controlled trial to determine the safety of early antibiotic use in ELBW infants.

**Methods:**

The NICU Antibiotics and Outcomes (NANO) trial is a multicenter, double-blinded, randomized controlled trial. A sample of 802 ELBW preterm infants will undergo web-based stratified block randomization to receive empiric antibiotics (EA; ampicillin and gentamicin) or placebo during routine evaluation for early-onset sepsis. Participating sites will use preexisting institutional protocols for antibiotic dosage and duration. Infants born at participating sites with a gestational age of 29 weeks or less are eligible for enrollment. Exclusion criteria include maternal intrauterine infection, hemodynamic or respiratory instability, delivery by caesarean section for maternal indications without labor or prolonged rupture of membranes, and prior administration of antibiotics. The primary outcome is the composite incidence of necrotizing enterocolitis, late-onset sepsis, or death during participants’ index hospitalization. Maternal and infant samples will be collected longitudinally and assessed for differences in microbiome composition and diversity.

**Discussion:**

The NANO trial is designed to compare the rate of adverse outcomes of EA use at birth versus placebo in ELBW preterm infants. If EA at birth worsens clinical outcomes, then the results of the trial may help providers decrease antibiotic utilization in the NICU and subsequently decrease the incidence of complications associated with early antibiotic use in ELBW infants. If we instead find that EA improve outcomes, then the trial will validate a longstanding clinical practice that has not previously been supported by high-quality data. Future studies will assess long-term clinical and microbial outcomes in infants who received empiric antibiotics following delivery.

**Trial registration:**

Trial registration data: June 25, 2019 NCT03997266.

**Supplementary Information:**

The online version contains supplementary material available at 10.1186/s13063-022-06352-3.

## Administrative information

Note: the numbers in curly brackets in this protocol refer to SPIRIT checklist item numbers. The order of the items has been modified to group similar items (see https://urldefense.com/v3/__http://www.equator-network.org/reporting-guidelines/spirit-2727-statement-defining-standard-protocol-items-for-clinical-trials/__;!!NHLzug!fX9TitmC09B4J-g2_vv2xMZQnOBHvBDPjxvG8ruoEqp1p_9jydyIixXY9Gvvs98 )

**Table:** 

Title {1}	NICU Antibiotics and Outcomes (NANO) Trial: A Randomized Multi-center Clinical Trial Assessing Empiric Antibiotics and Clinical Outcomes in Newborn Preterm Infants
Trial registration {2a and 2b}.	NCT03997266 PRO18010284
Protocol version {3}	Version 5/September 2020
Funding {4}	March of Dimes Research Grant #25-FY20-14 1R01HD097578-01 (US NIH Grant/Contract)
Author details {5a}	Michael J. Morowitz, MD, FACS University of Pittsburgh School of Medicine Division of Pediatric General and Thoracic Surgery Children’s Hospital of Pittsburgh of UPMC Anup C. Katheria, MD Attending Neonatologist, Division of Pediatrics Sharp Mary Birch Hospital for Women & Newborns Richard A. Polin, MD Attending Neonatologist, Division of Pediatrics The Trustees of Columbia University in the City of New York David T. Huang, MD MPH University of Pittsburgh Department of Critical Care Medicine Professor of Critical Care Medicine, Emergency Medicine, Clinical and Translational Science Chung-Chou H. Chang, PhD University of Pittsburgh Department of General Internal Medicine Professor of Medicine, Biostatistics, and Clinical and Translational Science Jonathan G. Yabes, PhD University of Pittsburgh Department of General Internal Medicine Assistant Professor of Medicine, Biostatistics, and Clinical and Translational Science Elizabeth Pace, MD University of Pittsburgh School of Medicine Department of Surgery
Name and contact information for the trial sponsor {5b}	Marion W Koso-Thomas, MD Eunice Kennedy Shriver National Institute Marion.koso-thomas@nih.gov 301-435-6873
Role of sponsor {5c}	Funding, monitoring of trial conduct

## Introduction

### Background and rationale {6a}

Due to advances in obstetrics and neonatology, increasing numbers of extremely premature infants now survive [1]. Because of risk factors associated with preterm birth (e.g., intraamniotic infection, preterm labor, and preterm premature rupture of membranes), NICU providers frequently administer broad-spectrum antibiotics to premature low-birthweight infants following delivery [2–4]. Many population-based studies have indicated that 2–4% of extremely-low-birthweight (ELBW) infants develop early-onset sepsis (EOS), a life-threatening vertically transmitted infection most commonly caused by GBS or *E. coli* [5]. Despite the low incidence of EOS, many clinicians choose to treat high-risk populations for EOS with broad-spectrum antibiotics while awaiting blood culture results to document the presence or absence of bacteremia [5]. However, recent observational studies indicate that administration of empiric antibiotics to this population may paradoxically increase the risk of adverse outcomes including late-onset sepsis, necrotizing enterocolitis (NEC), and death [6–10].

Antibiotics are the most commonly administered medications within neonatal ICUs (NICUs) [3]. Despite implementation of antimicrobial stewardship programs, antibiotic utilization rates and prescribing patterns vary significantly. Schulman et al. demonstrated that while antibiotic utilization in Californian NICUs declined from 2013 to 2016, they noted a persistent lack of correlation between antibiotic usage and proven infection or necrotizing enterocolitis [11]. Similarly, in a multi-institutional study using structured self-assessments regarding newborn specific antimicrobial stewardship programs and NICU antibiotic use rates, approximately 75% of infants receiving antibiotics for greater than 48 h from 143 participating medical centers did not have culture-proven infections [12]. These studies demonstrate that providers continue to prescribe antibiotics to uninfected preterm neonates irrespective of the adoption of antimicrobial stewardship programs.

The infant gut microbiome is dynamic during the first 3 years of life and is believed to be important for healthy infant development. Preterm birth, caesarean delivery, and the use of artificial infant formula instead of maternal milk can each disrupt early patterns of gut microbial colonization [13]. Antibiotic treatment is also known to significantly disrupt the microbiome for an extended period of time. The use of antibiotic therapies in children has been associated with a delay in microbiota maturation as well as reduced diversity of bacterial species and strains [14–17]. Furthermore, disruptions in the microbiome have been associated with higher incidences of common morbidities (bronchopulmonary dysplasia and necrotizing enterocolitis). However, unlike clinical factors which cannot be modified (e.g., prematurity), variations in antibiotic utilization rates suggest that antibiotic treatment is a risk factor for a disturbed microbiome (dysbiosis) that can potentially be mitigated. This is important not only because the microbiome contributes to infant development, but also because numerous studies have identified specific associations between intestinal dysbiosis and the occurrence of late-onset sepsis and NEC [18–21].

We designed a prospective multi-institutional randomized controlled trial to address the current issue of early antibiotic use in ELBW infants born with a gestational age less than 29 weeks. Based on current data regarding the negative effects of empiric antibiotic administration on the intestinal microbiome, we also seek to better characterize infant microbiota following perinatal antimicrobial administration [14–16]. Completion of The NICU Antibiotics and Outcomes (NANO) trial will ultimately help to guide antibiotic usage in NICUs and may improve outcomes for extremely preterm infants.

### Objectives {7}

The primary objective of the NANO trial is to test the hypothesis that the composite incidence of late-onset sepsis (LOS), NEC, or death in ELBW premature infants who are randomized to receive empiric antibiotics at birth is significantly different than in infants who receive placebo.

The second objective of the trial is to compare early patterns of gut microbial colonization in premature infants following randomization. We hypothesize that fecal samples obtained in the first month of life from infants receiving empiric antibiotics will contain lower diversity, higher abundance of pathogenic organisms, and lower abundance of commensal anaerobes compared to fecal samples from infants who receive placebo. A final exploratory objective of the trial is to identify microbial taxa associated with delayed or accelerated somatic growth based on weekly weight and length measurements during the first month of life in infants receiving early antibiotics or placebo.

### Trial design {8}

The NANO trial is a multicenter, double-blinded, superiority randomized clinical trial. The trial will utilize parallel groups of infant participants who receive either standard-of-care empiric antibiotics or placebo following randomization. A placebo concurrent control will be utilized given current conflicting data regarding the use and duration of empiric antimicrobial therapy for preterm ELBW neonates [22–24]. Participants will be randomized using 1:1 web-based block randomization stratified by study site to receive study drugs or placebo following eligibility screening and maternal consent. Stratified and block randomization will be used to minimize sample size imbalance between study arms within the different study sites participating in the trial. The trial was registered at ClinicalTrials.gov prior to initiation of participant enrollment.

## Methods: participants, interventions, and outcomes

### Study setting {9}

NANO study sites are university-affiliated academic teaching hospitals and hospital-based birthing centers in the USA and Canada. Sites were selected to ensure an adequate infrastructure needed for completion of the study protocol and recruitment of maternal and infant participants. Institutions were also selected in order to maximize the geographic diversity of our study sites to assist with the trial’s generalizability. We estimated site-specific patterns of enrollment based upon the number of inborn infants admitted to each site with an estimated gestational age of 23–28 weeks. A complete list of the IRB-approved NANO study sites may be found on the corresponding NIH sponsored ClinicalTrials.gov web portal.

### Eligibility criteria {10}

Maternal and infant inclusion and exclusion criteria ensure that enrolled preterm infants are those patients for whom the risks and benefits of receiving empiric antibiotics remains unclear, and clinical equipoise exists. Clinically stable infants born at a gestational age of 23 to 28 6/7 weeks are eligible for enrollment. Infants are to be excluded in the presence of ongoing or worsening respiratory insufficiency, hemodynamic instability, and/or clinical concern for sepsis based on physical exam findings. Additional infant exclusion criteria are utilized to avoid enrolling infants at high risk for EOS who will likely require antibiotics, and to avoid enrolling infants at low risk for EOS who likely will not receive empiric antibiotics at birth [25, 26]. High risk for EOS is defined by the presence of maternal intrapartum fever or diagnosis of chorioamnionitis [27]. Low risk for EOS is defined by caesarean delivery for maternal indications without attempts to induce labor and without rupture of membranes >6 h prior to delivery [28, 29]. Maternal and infant eligibility criteria are further detailed in Table 1. Study sites were eligible for participation if they had a prior history of successful participation in clinical trials involving neonates and were willing to randomize infants to the intervention (administration of ampicillin and gentamicin) and control arms.

**Table 1:** Eligibility criteria

**Infant inclusion criteria**	Newborn infants born with gestational age 23–28 6/7 weeks
	Infants delivered at participating study sites
**Infant exclusion criteria**	Infants at *low risk* for early-onset-sepsis: infants born for maternal indications via caesarean section with rupture of membranes within 6 h of delivery, no attempts to induce labor, or no concern for maternal infection
	Infants at *high risk* for early-onset-sepsis: infants born to mothers with intrapartum fever >38 °C or infants with clinical diagnosis of chorioamnionitis (suspected or definite)
	Infants with respiratory insufficiency requiring invasive mechanical ventilation and FiO2 > 0.40 or non-invasive ventilation (i.e., CPAP) with FiO2 > 0.60 *at time of randomization*
	Infants with ongoing hemodynamic instability requiring vasopressors or more than one 10 ml/kg NS bolus *at time of randomization*
	Clinician concern for sepsis due to physical exam findings (i.e., minimal responsiveness, poor tone)
	Major congenital abnormalities (i.e., cardiac, pulmonary, gastrointestinal anomalies)
	Infants not anticipated to survive beyond 72 h
	Infants who have received antibiotics prior to randomization
**Maternal exclusion criteria**	Mothers that are <18 years old at time of consent

*FiO*_*2*_ fraction of inspired oxygen, *CPAP* continuous positive airway pressure

### Who will take informed consent? {26a}

Individualized study site protocols will be created to enable identification of eligible women who are expected to deliver an infant between 23 and 28 6/7-week gestation. If women pass the initial eligibility screening, study site coordinators and physician investigators will discuss study participation including informed consent. Coordinators and investigators will provide a study brochure as well as an IRB-approved informational video that summarizes trial enrollment, risks and benefits of participation, and the trial protocol including required blood and stool samples. Per standard practices, consent from one parent of potential infant participants will be sufficient for enrollment in the trial. The full consent form, which details these elements, may be found in the supplementary materials.

When antenatal consent is obtained, investigators will track and monitor maternal subjects during their hospital admission. If study participants are discharged prior to delivery, coordinators and investigators will monitor for readmission and subsequent delivery. Maternal participant’s initial informed consent will remain valid if discharged and subsequently readmitted to a participating study site. If potentially eligible infants are born to women that were not screened previously for study participation and are identified within 4 h after delivery, coordinators and investigators may attempt to obtain postnatal informed consent for study participation.

### Additional consent provisions for collection and use of participant data and biological specimens {26b}

Informed consent documentation will disclose the possible use of de-identified maternal and infant data and biological specimens in future and/or ancillary studies as per NIH Guidelines regarding biospecimen storage and tracking [30]. Participant outcome data, stool specimens, and genetic data derived from infant blood samples may be shared with other researchers and federal repositories following approval by the NANO Steering Committee. Genetic data will be de-identified and stored according to the NIH Genomic Data Sharing Policy. Biospecimen storage, tracking, and security are further detailed in the NANO Standardized Operating Protocol (SOP) that is provided as a supplementary material.

## Interventions

### Explanation for the choice of comparators {6b}

We chose to compare EA to placebo (normal saline equivalent) given the lack of data demonstrating clinical benefits associated with antibiotic administration. As demonstrated by recent observational studies, current evidence regarding the risks and benefits of EA administration in preterm ELBW infants continues to remain in flux [8, 10, 31]. Furthermore, current antibiotic stewardship efforts focus primarily on shortening the duration of EA, and do not address the possibility of eliminating EA altogether in preterm ELBW infants [12, 32]. Our use of ampicillin and gentamicin as empiric antibiotics is supported by various studies demonstrating that the microbiology of EOS has remained largely unchanged in the USA over the past decade [33]. *Escherichia coli* and Group B Streptococcus remain the most common bacterial isolates among very-low-birthweight preterm infants diagnosed with EOS, followed by other gram-negative organisms. The combination of ampicillin and gentamicin is commonly used by neonatologists for empiric antimicrobial therapy of EOS given its sensitivity against Group B Streptococcus, enterococcal species, and Listeria monocytogenes [28, 34–36].

EOS remains poorly defined in current literature, with treating physicians and institutions choosing to utilize various clinical signs, laboratory, and microbiology data as evidence for neonatal sepsis. Currently, the isolation of an infective organism in either blood or cerebrospinal fluid remains as one of the most common criteria for diagnosing neonatal sepsis [37]. Various studies have demonstrated that the overwhelming majority of ELBW infants receiving empiric antibiotics do not have culture-positive EOS [38, 39]. In a study of 227 well-appearing term and late preterm infants exposed to chorioamnionitis but not treated with empiric antibiotics, there were no documented cases of culture-positive EOS [40]. Thus, even in those well-appearing infants with maternal risk factors for EOS, such as chorioamnionitis or premature ROM, the use of antibiotics may not prove necessary.

### Intervention description {11a}

Following determination of maternal and infant trial eligibility, infants will be randomized by IRB-approved study site investigators into one of two study groups: empiric antibiotics (parenteral ampicillin and gentamicin) or volume-matched placebo (normal saline). Antibiotics used during the trial will be commercially available medications at each study site. Dosages and dosing intervals of ampicillin and gentamicin will be based on local site-approved dosing guidelines and protocols documented in each of the study sites’ standard operating protocols. Infants randomized to receive placebo will receive volume-matched equivalents of normal saline on an analogous schedule to that of ampicillin and gentamicin administration.

Randomization will take place within the first 4 h of life. Infants will then receive their first dose of unlabeled study drug (ampicillin and gentamicin, or placebo) within 120 min of randomization by registered neonatal ICU nurses. Subjects will continue to receive either empiric antibiotics or placebo as originally assigned until the end of the study drug administration period. Following the intervention period, administration of further antibiotics will be at the discretion of treating neonatologists. A timeline of study interventions for maternal and infant participants can be seen in additional files [see Additional file 1].

### Criteria for discontinuing or modifying allocated interventions {11b}

We anticipate that <5% of infant subjects will experience worsening of conditions prompting clinicians to order additional antibiotics that will be termed “rescue antibiotics.” Clinicians will be allowed to order rescue antibiotics as they see fit, with no restrictions imposed by the study protocol. Should this occur, the treating physicians will remain blinded to the initial study drug assignment and study pharmacists will be unblinded. Patients who have received a rescue option will continue to be followed until discharge from the NICU.

There are two anticipated clinical scenarios where rescue antibiotics may be required. The first scenario will involve an attending neonatologist wishing to guarantee the infant receives ampicillin and gentamicin and not only placebo. When this occurs, a second set of study drug orders will be placed. If the infant’s original assignment was placebo, pharmacy will prepare unlabeled ampicillin and gentamicin. If the original assignment was ampicillin and gentamicin, pharmacy will prepare unlabeled placebo and placebo. Investigational pharmacists will then proceed to assist with appropriate study drug dosing based on the infant’s initial treatment arm assignment. This will allow study investigators to remain blinded to participants’ allocation.

The second rescue scenario will involve a decision by an attending neonatologist to empirically treat suspected or confirmed bacterial threats not optimally covered by ampicillin and gentamicin with open-label non-study antibacterial (e.g., penicillin for congenital syphilis, meropenem for ESBL producing *E. coli*). These are ordered outside of the study per routine care and are not the responsibility of the site investigational drug service. Treating physicians may also choose to discontinue study medications prior to the end of the study drug period for infants who are deemed to be at low risk of developing EOS following randomization.

### Strategies to improve adherence to interventions {11c}

The NANO Clinical Coordinating Center (CCC) at the University of Pittsburgh will provide formal training to team members at all approved study sites. The training will detail all aspects of the study protocol and carefully review the process for data entry using the secure web-based trial portal. For all maternal and infant subjects, trial timepoints including the time of randomization and time of study drug administration will be recorded into the portal and monitored for protocol deviations. Coordinators and investigators will be responsible for documentation of study drug orders, including the discontinuation of study drugs and use of rescue antibiotics. The CCC will promote compliance with regular feedback to each study site and identify solutions for rectifying non-compliance. The CCC will also continue to track site performance on a monthly basis with standardized reports of study enrollments as well as protocol deviations. Clinical coordinators and study investigators will routinely communicate to help minimize protocol deviations and to address ongoing concerns or questions pertaining to the trial protocol.

### Relevant concomitant care permitted or prohibited during the trial {11d}

Maternal and infant participants will receive standard-of-care medical therapies following enrollment into the NANO trial. Therapies include but are not limited to blood draws, urine collections, administration of intravenous fluids, and enteral nutrition as deemed appropriate by treating physicians. Infants may undergo invasive procedures and interventions as indicated. Sites will also be allowed to administer probiotics to infants if currently utilized in institutional protocols. Recent animal models and clinical studies have demonstrated conflicting evidence regarding probiotics and their association with the incidence of necrotizing enterocolitis, sepsis, and mortality [41]. Use of probiotics by study sites will be accounted for in secondary statistical analyses.

### Provisions for post-trial care {30}

The NANO trial has no provisions for ancillary or post-trial care. Participants that experience adverse events during trial participation will receive standard-of-care medical therapies from each of the individual study sites by licensed healthcare providers. Neither maternal nor infant participants will receive monetary compensation for incurring harm due to the NANO trial.

### Outcomes {12}

#### Primary outcome

The primary outcome of the NANO trial is the composite incidence of NEC, LOS, or death during an infant’s index hospitalization. The use of a composite incidence of outcomes as the study’s primary outcome was based on the observed individual incidence rates of NEC, LOS, and mortality in preterm neonates [42–44]. Given reported low incidence rates of NEC, LOS, or mortality in neonates, the use of a composite outcome increased the feasibility of achieving an adequate sample size and proceeding with a randomized clinical trial.

We define NEC by Bell’s stage II or III criteria for moderate or advanced NEC in infants who are greater than 7 days of age [45, 46]. ELBW infants presenting with similar symptoms at less than 7 days of age will be considered to have spontaneous intestinal perforation unless proven otherwise during subsequent laparotomy, given observational studies demonstrating an earlier presentation of intestinal perforation versus NEC in premature infants [47–49]. We define LOS as a positive blood culture obtained after 72 h of life that results in treatment with antibiotics for 5 days or more [37].

#### Secondary outcomes

Secondary outcomes are the individual incidence of NEC, LOS, and death during infants’ index hospitalizations. The microbiome analysis endpoints are (1) the alpha diversity (Richness and Shannon Index), (2) the beta diversity, and (3) the differential abundance of individual bacterial taxa.

### Participant timeline {13}

Table 2 shows the intervention schedule.

**Table 2:** Intervention schedule

TIMEPOINT	STUDY PERIOD							
	Prenatal enrollment	Postnatal enrollment	Allocation	Post-allocation				Close-out
			4 h after delivery	***2 h***	***24 h***	***36 h***	***48 h***	***>48 h***
**ENROLLMENT:**								
**Eligibility screen**	X	X						
**Informed consent**	X	X						
**Allocation**			X					
**Study drug administration**				X	X	X	X	
**INTERVENTIONS:**								
**Empiric antibiotics**				X	X	X	X	
**Placebo**				X	X	X	X	
**Rescue antibiotics**				*	*	*	*	
**ASSESSMENTS:**								
**Demographic variables**	X	X	X					
**Incidence of NEC, LOS, death**			X	X	X	X	X	X
**Infant fecal samples**					X			X
**Infant blood sample**					X			
**Maternal fecal samples**					X			
**Maternal vaginal swab**					X			
**Infant weight and length measurements**					X			X

^*^Administration of rescue antibiotics determined by site physician investigators

### Sample size {14}

Sample size was calculated based on our primary hypothesis that the incidence of composite adverse events including NEC, LOS, and/or death is significantly different in ELBW infants that receive empiric antibiotics versus infants that receive placebo. Based on 1,000,000 simulations for the group-sequential test for comparing two proportions and the O’Brien-Fleming alpha spending method for two interim analyses, we need 382 infants in each arm to reach 90% power to test the primary hypothesis using a two-sided significance level of 0.05. Anticipating a 5% attrition rate, we will recruit 802 infants to reach at least 90% power.

Most or all ELBW infants have historically received empiric antibiotics, and most published studies of clinical outcomes for ELBW infants have not separated those babies born at high or low risk for EOS. Thus, to our knowledge, there is no perfect benchmark to guide estimates of the incidence of LOS, NEC, and death in the NANO study population. To calculate event rates in this study, we used data from the Vermont Oxford Network and Pediatrix Clinical Data Warehouse (CDW) after attempting to recapitulate NANO inclusion and exclusion criteria. We conservatively estimate that the composite incidence of LOS, NEC, or death in infants receiving empiric antibiotics will be 22%, which is similar to incidence rates seen in data from the CDW. To estimate the effect size between experimental groups, we considered published data regarding the incremental daily increased risk of adverse events with administration of antibiotics [50, 51]. We estimate the odds of the composite outcome will increase by 1.35 with each day of antibiotic therapy assuming a total of 48 h of antibiotic administration. Thus, we estimated incidence of the composite outcome in infants that do not receive antibiotics as approximately 13.5%.

For microbiome analyses, assuming that the alpha diversity is normally distributed, we will need a total sample size of 78 over two groups to detect a 43% reduction in alpha diversity at a 14-day timepoint with a 5% level of significance and 90% power. The effect size is based on previously published reports demonstrating the negative effects of antibiotic administration on the human microbiome [52–54]. We will exceed power to discern differences in alpha diversity, but will plan for much larger sample size to allow not only for taxonomic analyses across samples from the overall EA and placebo groups, but also subgroups (e.g., caesarean delivery only).

### Recruitment {15}

IRB-approved study coordinators and physician investigators at each site will utilize electronic medical records and direct communication to identify and recruit potential maternal and infant participants. Individualized site protocols within obstetric units will be created at each of the study sites to enable consistent identification of eligible women who are likely to deliver an infant at or before 28 6/7week gestation. Recruitment strategies may include review of admission to obstetric triage as well as labor and delivery units for potential maternal participants and neonatology consultation. Women presenting within this gestational age window routinely meet with one or more neonatologists prior to delivery. At the time of consultation, neonatologist co-investigators and research staff will provide women with informational study brochures and a link to the NANO trial video. Study team members will remain available for consultation with maternal participants throughout their hospital admission to answer questions and provide reassurance as needed relating to the NANO trial.

Each NANO study site will maintain screening logs to assist with participant recruitment. Screening logs will contain de-identified data including maternal age, maternal race, and screening date. Sites will submit screening logs on regular intervals to the CCC for review. Overall and individual site enrollment will be closely monitored throughout the trial period. If we are unable to recruit sufficient numbers of participants to reach the target sample size, additional sites will be added as needed following approval by the NANO Steering Committee.

## Assignment of interventions: allocation

### Sequence generation {16a}

After parental consent has been obtained, study coordinators and investigators will continually monitor maternal participants to ensure that they remain eligible for participation (e.g., absence of chorioamnionitis). When infants are delivered by maternal participants, the infants likewise will be assessed for eligibility (Table 1). Staff will subsequently input patient identification information including medical record number and/or birth date into the study’s secure web-based portal. For mothers and infants meeting all criteria for participation, the family will be randomized 1:1 using web-based block randomization with random block sizes from 2 to 6 stratified by study site to the different treatment arms of ampicillin and gentamicin or volume-matched equivalent of normal saline (placebo). As such, multiples (siblings) will be randomized to the same treatment arm which would limit parental consent burden and cross-contamination between arms. In addition, randomizing multiples to the same treatment arm is commonly utilized due to their similar genetic backgrounds as well as prenatal exposures.

### Concealment mechanism {16b}

Investigational pharmacists at each study site will be the only clinical providers unblinded to infant treatment arm assignments. Following computer-generated block randomization, investigational pharmacists will receive a secure notification from the study portal regarding final treatment allocation as well as pertinent patient data including weight (kg) for dosing purposes. Investigators who performed the initial randomization will receive an electronic confirmatory message via the trial portal if the pharmacy was appropriately notified and randomization was successful. Pharmacists will then proceed to formulate ampicillin and gentamicin or placebo (normal saline equivalent) based on previously determined study site antibiotic weight-based dosages. Unlabeled study drugs will then be delivered to the infant’s hospital room for prompt administration by a registered NICU nurse.

### Implementation {16c}

In summary, IRB-approved study site coordinators and physician investigators will work with their fellow obstetricians and neonatologists to identify and recruit mothers of infants with an estimated gestational age of 23 to 28 6/7 weeks. Following successful enrollment into the trial after maternal and infant eligibility screening, site coordinators or investigators will input participant data in the secure web-based trial portal for randomization. The computerized portal system will generate a secure allocation sequence based on 1:1 block randomization stratified by study site. Investigational pharmacists will be securely notified as to infant participant allocation and will subsequently prepare study drugs as indicated.

## Assignment of interventions: blinding

### Who will be blinded {17a}

Infant participants will receive unlabeled study medications formulated by investigational pharmacists according to their computer-based allocation. Medications will be formulated by investigational pharmacists to ensure that they are undistinguishable by investigators and nursing staff, including temperature regulation and storage. Maternal and infant participants, clinical coordinators, study personnel, and physician investigators will remain blinded to participant treatment arm assignments throughout the duration of the trial. In the setting of the need to administer “rescue antibiotics,” healthcare providers will continue to remain blinded to infants’ initial treatment allocation. Investigational pharmacists will assist as needed in the formulation of additional ampicillin and/or gentamicin based on the infant’s initial treatment arm allocation.

Following the study drug administration period, participants will continue to remain blinded to infants’ allocation until completion of the trial. Unblinded data evaluation during the active recruitment phase of the trial will be restricted to a designated study statistician and the DSMB. Investigators will be unblinded when all data collection is completed, and the web-based data collection system is locked for final analysis.

### Procedure for unblinding if needed {17b}

Unblinding is not recommended throughout the duration of the NANO trial. Administration of antibiotics beyond the study drug period will be at the discretion of healthcare providers at each of the study sites. In situations in which NICU providers wish to continue the use of gentamicin following the completion of the study period, they will require site-specific gentamicin therapeutic drug monitoring. This poses an unavoidable risk for discerning treatment allocation of study participants by clinical pharmacists and healthcare providers. The study protocol recommends documenting clinical pharmacokinetic assessments in a patient’s secure web-based NANO dispensing log to decrease the risk of unblinding and to continue to utilize investigational pharmacists as able to assist with gentamicin level monitoring.

## Data collection and management

### Plans for assessment and collection of outcomes {18a}

Study site coordinators and investigators will undergo data management training by the CCC prior to the initiation of the trial. Training will consist of a review of the secure web-based portal created for the NANO trial, including web-based data collection forms, online reporting of adverse events, documentation of specimen collections, and weekly infant length and weight data entry. The CCC will oversee the creation and management of the NANO trial web-based portal and make changes to data collection forms as needed based on feedback from the study sites.

Trained study site coordinators and co-investigators will obtain de-identified baseline demographic and clinical information via medical chart review and patient and/or physician interviews following trial enrollment. An encoding table will assign an individual study number to every participant’s name and medical record at each institution. All relevant clinical and trial data will be entered into web-based forms on the trial’s secure web-based portal by study site coordinators and investigators. The portal will utilize primarily drop-down selections and multiple-choice responses and provide automatic prompting for missing variables. Variables that will be inputted into data collection forms for mother and infant pairs are listed in Table 3.

**Table 3:** Data variables

Data variables for data collection form		
**Maternal**	**Demographics**	DOB, race, highest education level
	**Lifestyle**	Tobacco and alcohol use, BMI
	**Current pregnancy history**	Final antepartum admission date, number of fetus(es), prenatal obstetrics visit, history of spontaneous preterm birth
	**Diagnoses** (Yes/No)	Insulin-dependent diabetes, maternal hypertension, preeclampsia, antepartum hemorrhage, placenta previa, abruptio placenta, fetal growth restriction, cervical insufficiency, preterm labor
	**Antibiotics administered within 30 days of delivery**	Diagnoses: positive urine cultures, genital tract infection, skin/soft tissue infection, vaginal yeast infection, PPROM, GBS, presumed or confirmed intraamniotic infection or chorioamnionitis; drug names, length of treatment
	**Other drugs**	Tocolytic therapy, betamethasone, antenatal corticosteroids; drug names, length of treatment
**Infant baseline**	**Delivery**	Date, time, gestational age, sex, birth weight (kg)
	**Randomization**	Date
**Infant hospital course**	**Outcomes at 1 week of age**	EOS diagnosis 1. If Yes: causative organism, antibiotics and dates of treatment Days of antibiotics received during week 1 of life
	**Weekly**	Weight (kg), length (cm), LOS diagnosis (Yes/No), NEC diagnosis (Yes/No), death (Yes/No)
	**Nutrition (days 3, 7, 14, 28, 60)**	Enteral nutrition, type of milk or formula received, date subject reached full enteral feedings
	**Discharge**	Date of discharge, culture results, diagnoses: Grade 3 or 4 IVH, ROP, CLD, days of endotracheal intubation, positive blood cultures, positive respiratory cultures, positive urine cultures, positive cerebrospinal fluid cultures
	**Antibiotics prescribed**	Diagnosis, drug name, total number of days administered
	**IV antifungal medications**	Total number of days prescribed

### Plans to promote participant retention and complete follow-up {18b}

The NANO trial will maximize both enrollment and participant retention via consistent and reliable communication between parents and physician investigators responsible for the care of their infants. Each institution will develop site-specific communication strategies to optimize participant retention. Such strategies will include clear communication of the study protocol, including timing and frequency of sample collections, provision of contact information for local research coordinators and/or investigators, and continued reinforcement of the trial’s goal to improve outcomes for preterm infants. Research staff will also strive to preserve and respect the privacy of participants and their families throughout the study period.

Infant participants will be monitored throughout their hospitalization in the NICU following the study drug administration period, ensuring complete study follow-up. Data regarding the incidence of the primary and secondary outcomes will be obtained by study site coordinators and/or investigators regardless of discontinuation or deviations from the study protocol, including administration of rescue antibiotics or cessation of study drugs prior to the end of the study period. Outcomes will also still be assessed despite the continuation of antibiotic therapies following the end of the study drug period; total days of antibiotic administration and the type and dosage of antibiotic therapies will be recorded for infants receiving additional antibiotics. Fecal samples will also be obtained from these infants for subsequent microbiome analyses.

If parents choose to refuse or withdraw consent for infant participants following enrollment into the trial, a limited set of demographic data, such as sex, age, and race, will be collected via site screening logs. This information will allow for comparison of patients who did and did not successfully enroll in the study, permitting the analysis of potential selection biases and the generalizability of the final study results. Collection of information will be determined by the individual study site’s IRB approval.

### Data management {19}

The NANO trial’s primary data entry system will be a secure online-based portal accessible via a password-protected study website that will be maintained via the Data Coordinating Center at the University of Pittsburgh CRISMA Biostatistics and Data Management Core. The portal will be used for patient randomization, electronic data collection forms, tracking logs for sample collection and shipment, as well as storage of study-related documents. Access will be restricted to study investigators, research staff, and committee members via unique usernames and passwords. Clinical site coordinators and additional trained research staff will be responsible for ensuring that all necessary patient data is collected and entered into the electronic data collection form. Required patient data variables can be seen in Table 3. Clinical coordinators will also be responsible for completing and maintaining all mandatory source documents in each of the participants’ research records.

### Confidentiality {27}

Personal information of potential and enrolled participants will be collected by IRB-approved study site coordinators and investigators and input into the secure web-based NANO portal system. Only approved research staff will have access to participants’ personal information.

Cryopreserved patient stool and blood samples will be de-identified such that no patient identifier information is accessible. A key of de-identified samples will be maintained on a secure password-protected institutional server at The University of Pittsburgh until the completion of the NANO trial. De-identified patient samples may be released or studied only with express written consent of the NANO Steering Committee. All banked specimens will be stored indefinitely at The University of Pittsburgh.

### Plans for collection, laboratory evaluation and storage of biological specimens for genetic or molecular analysis in this trial/future use {33}

One to two spontaneously expelled infant fecal samples will be obtained weekly by nursing staff until 8 weeks of life or discharge from the NICU for microbiome analyses. If infant participants remain in the NICU for longer than 8 weeks, monthly fecal samples will be collected until discharge. Study site coordinators and research personnel will store fecal samples in predesignated research areas prior to being shipped to the CCC. If a subject is diagnosed with NEC or LOS, additional stool samples may be collected. In addition to stool, one infant blood draw will be requested within the first week of life during the time of other clinical blood draws for genetic analyses. A volume of 0.3 to 0.4 mL will be drawn by trained NICU personnel who routinely perform blood draws per institution-specific protocols. Blood draws may be limited by treating providers as indicated based on the infant’s clinical status. After blood samples are collected, they will be frozen and stored prior to shipment to the CCC for genomic analyses.

Study sites with the ability to obtain maternal intrapartum vaginal and rectal swabs for microbiome analyses by obstetric co-investigators will be identified prior to study site on-boarding and NANO training. If vaginal and/or rectal swabs cannot be obtained due to lack of study site infrastructure or research personnel availability, a postpartum maternal fecal sample will be obtained via self-collection. Fecal samples will be obtained within 1 week postpartum and subsequently sent to the CCC for microbiome analyses following site storage protocols.

Further details involving biospecimen collection and storage may be found in the trial’s manual of operating procedures (MOP) as listed in the supplementary materials.

## Statistical methods

### Statistical methods for primary and secondary outcomes {20a}

The distribution of baseline variables between study arms will be evaluated for successful randomization. The primary analysis is an intent-to-treat (ITT) analysis that includes two interim analyses at 1/3 and 2/3 of enrollment using O'Brien-Fleming stopping rules, and a final analysis. The primary outcome will be analyzed using a generalized linear model (GLM) with a log link fitted via generalized estimating equations (GEE) with exchangeable working correlation matrix and employing robust variance estimates. This will account for non-independence of observations due to clustering of infants within families. The model will include treatment as a fixed effect adjusted for site and gestational age. Our primary hypothesis that the rate of composite adverse events (NEC, LOS, and mortality) differ between infants receiving empiric antibiotics compared to those receiving placebo will be tested via the Wald test of the treatment assignment. Effect estimates will be presented using risk ratios (RR) with 95% confidence intervals (CI).

In secondary analyses, we will examine each component (NEC, LOS, death) of the composite adverse outcome separately using the same analytic approaches as the primary outcome.

### Interim analyses {21b}

Two planned interim analyses prior to the final analysis will be performed by the NANO Data Coordinating Center at The University of Pittsburgh. Analyses will occur at 1/3 and 2/3 of expected participant enrollment. The DSMB will be responsible for reviewing overall recruitment, safety, and data collection at each of the interim analyses. The DSMB and a designated study statistician will have access to unblinded study data to allow for interim data analyses using O’Brien and Fleming stopping rules defined a priori controlling for an overall Type I error of 0.05. As demonstrated in Table 4, the DSMB will make their final recommendations regarding the results of interim data analyses and may recommend early trial termination with unanticipated safety concerns, including an increased incidence of composite adverse outcome in either the EA or placebo arm. They may also recommend cessation of the trial if it is unable to successfully recruit participants, encounters improper data handling, or is not able to successfully implement study protocols.

**Table 4:** Stopping rules based on the O’Brien-Fleming alpha spending method

Interim	Decision action	***Z*** test statistic boundary value^**a**^	***P*** value
**First**	§ Stop the trial due to higher incidence of composite adverse events in EA	*Z* > 3.66474	*P* < 0.00025
	§ Stop the trial due to higher incidence of composite adverse events in placebo	*Z* < −3.66474	*P* < 0.00025
	§ Continue the trial	*Z* in [−3.66474, 3.66474]	*P*^3^ 0.00025
**Second**	§ Stop the trial due to higher incidence of composite adverse events in EA	*Z* > 2.50210	*P* < 0.01235
	§ Stop the trial due to higher incidence of composite adverse events in placebo	*Z* < −2.50210	*P* < 0.01235
	§ Continue the trial	*Z* in [−2.50210, 2.50210]	*P*^3^ 0.01235
**Final**	§ Incidence of composite adverse events is significantly higher in EA	*Z* > 1.96	*P* < 0.05
	§ Incidence of composite adverse events is significantly higher in placebo	*Z* < −1.96	*P* < 0.05
	§ Incidence of composite adverse events is not significantly different between EA and placebo	*Z* in [−1.96, 1.96]	*P*^3^ 0.05

^a^*Z* test statistic = [(incidence of NEC/LOS/death in EA) − (incidence of NEC/LOS/death in placebo)]/SE

### Methods for additional analyses (e.g., subgroup analyses) {20b}

Subgroup analyses will be conducted to better understand treatment effects and to identify subgroups of patients for whom the treatment was particularly beneficial or harmful. Subgroups will be predefined to limit selection bias. Subgroups will include: study site, preterm rupture of membranes, infant sex, gestational age, delivery mode, preterm labor, maternal antenatal antibiotic doses, maternal socioeconomic status including education and insurance status, and infant diet (maternal milk, donor milk, or formula).

Bacterial DNA will be extracted from maternal and infant fecal samples and swabs, amplified, and sequenced according to established protocols. In analyses of these samples, we shall investigate alpha diversity (Richness and Shannon Index), beta diversity, and the differential abundance of individual bacterial taxa. Data from each experimental group will be compared at each time point during the infant’s first month of life as well as in longitudinal trend analyses conducted throughout the duration of the infant’s NICU admission. Alpha diversity, beta diversity, and abundance of taxa will be analyzed using linear mixed-effects models, PERMANOVA (permutational multivariate analysis of variance) for repeated measurements data, and ANCOM-BC (ANalysis of Composition Of Microbiomes with Bias Correction) respectively.

Weight, length, and head circumference *Z*-scores will be calculated for birth and weekly postnatal growth measurements using Fenton and Olsen growth curves for preterm infants [55]. A temporal growth curve model will be developed for each group of babies. We will use these growth models to compare the temporal differences in growth curve patterns between the two groups of infants.

### Methods in analysis to handle protocol non-adherence and any statistical methods to handle missing data {20c}

We will provide reasons for any missing data and summarize the proportion of patients with missing data for each outcome and by study arm and site. Baseline patient characteristics will be compared between those who have complete outcome data and those that do not. Multiple imputations using multivariate imputation by chained equations will be used to conduct ITT analyses in the case of missing participant data. Predictive mean matching and logistic regression will be used to impute continuous and binary outcomes, respectively. We will assume that missing outcome data are missing-at-random (MAR) and that they can be imputed reasonably well from the observed study data. Imputations will be performed based on the study arm to which the patient was originally assigned.

Given per-protocol analyses may suffer from selection bias, we will use an instrumental variable approach to estimate the complier average casual effect (CACE). The CACE will measure the impact of the treatment in the subgroup of the population that complies with the assigned treatment. The treatment assignment will be used as the instrument because its impact on outcome is expected to be entirely mediated through the receipt of the treatment.

### Plans to give access to the full protocol, participant level-data and statistical code {31c}

Trial protocol, data, and statistical code will be made available as per the NIH Policy on Dissemination of NIH-Funded Clinical Trial Information. Results from the trial will be submitted less than 1 year after the trial’s primary completion date.

## Oversight and monitoring

### Composition of the coordinating center and trial steering committee {5d}

The NANO trial will be led by the Clinical Coordinating Center (CCC) and Data Coordinating Center (DCC) at the University of Pittsburgh as well as the NANO Steering Committee. The primary decision-making body of the study will be the NANO Steering Committee, consisting of the primary investigators at the University of Pittsburgh, Mary Sharp Birch Hospital, and Columbia University.

The CCC will be responsible for the finalization of the clinical protocol and manual of operations, facilitation of study recruitment, data collection, and the organization and oversight of the individual clinical sites. The CCC will maintain close communication with all study sites via scheduled coordinator and co-investigator meetings. Furthermore, members of the CCC will perform site visits as needed to ensure adherence to the protocol and data management procedures. The CCC will also serve as the primary liaison to the central IRB at the University of Pittsburgh as well as the Data and Safety Monitoring Board (DSMB) and will complete and submit reports documenting enrollment, adverse events, site performance, and quality control as needed.

The NANO Steering Committee will be responsible for overseeing the trial and making decisions regarding protocol and data management system changes, as well as the recruitment of additional study sites. The committee will continuously monitor recruitment and work with each clinical site to reach recruitment goals. The Steering Committee will also serve as the Publication Committee and will ensure that the results from the NANO trial will be published in a manner consistent with CONSORT and ICMJE authorship guidelines. Secondary and ancillary studies will be evaluated and approved by the Steering Committee on an as needed basis.

### Composition of the data monitoring committee, its role and reporting structure {21a}

The University of Pittsburgh Office of Clinical Research will support the trial’s Data Safety and Monitoring Board (DSMB). The DSMB will be responsible for reviewing the study protocol prior to initiation of enrollment. The DSMB will also be responsible for reporting data and safety monitoring, adverse event data, an assessment of relevant scientific literature and its impact on the design of the study, and a summary of procedural reviews conducted to ensure subject privacy to the primary study site’s IRB.

The University of Pittsburgh will be the home of the NANO Data Coordinating Center (CRISMA BDMC) and the central Institutional Review Board (Pitt HRPO). The DCC will be responsible for assuring the standardization, collection, management, and quality control of the data as well as the statistical design and analysis of the study. The DCC will monitor the data collected from all participating study sites and subsequently entered into the NANO secure web-based portal.

### Adverse event reporting and harms {22}

All adverse events and serious adverse events will be recorded from the time of consent until hospital discharge. Events will be solicited from parents of infant subjects, attending physicians, and bedside nurses. The medical record will also be reviewed for presence of adverse events. Study sites are instructed to notify the CCC regarding any adverse event within 48 h of its recognition.

Adverse events that are unexpected and serious and suggest that the interventions place subjects at greater risk than previously recognized will be reported to the IRB within 30 days. External adverse events are to be reported to the respective study site’s IRB or research monitoring committee. The CCC will also be responsible for reporting any serious adverse event to the DSMB within 24–48 h of their knowledge of the event. After review of the SAE report, the DSMB may choose to call an emergency meeting to review the event. All other adverse events are to be recorded and logged for a cumulative quarterly adverse event report to the DSMB. All deaths will be reviewed by the DSMB within 30 days of the event.

Study site investigators are responsible for reviewing participant’s medical records, laboratory values, and radiographic data following the occurrence of an adverse event. Pertinent information as well as the investigators impression of the diagnosis will be recorded in the participant’s file on the secure study portal. The study site investigator will assess causality between the event and the study protocol using best clinical judgement. The DSMB will review the investigator’s findings and recommend follow-up or protocol modifications as needed.

### Frequency and plans for auditing trial conduct {23}

Study site screening logs, data entry, and participant enrollment will be continually monitored by the Clinical Coordinating Center. The CCC will conduct individual study site visits as needed if there is a concern that a site is not meeting trial expectations regarding enrollment and protocol adherence. The review process will be overseen by the NANO Steering Committee.

### Plans for communicating important protocol amendments to relevant parties (e.g., trial participants, ethical committees) {25}

The CCC will be responsible for disseminating information regarding study protocol amendments to all the study centers and responsible parties. The University of Pittsburgh SOP will be updated as needed and disbursed to the Steering Committee and study centers for review following the central IRB approval of protocol changes. The DSMB will be notified as needed as to major protocol amendments as determined by the primary investigators.

### Dissemination plans {31a}

Prior to study completion, examples of data collection forms and the study protocol will be available upon request for other members of the scientific community. The finalized NANO dataset will be made public with appropriate documentation and provided to the NICHD. The study investigators will disseminate study results and write manuscripts including primary results as well as other methodology and pre-planned analyses of secondary outcomes supported by The University of Pittsburgh BDMC. Following the publication of the primary results paper, an archived dataset with documentation will be made available for additional uses by outside investigators in collaboration with the study investigators.

The Publications and Presentations policies and procedures for the NANO trial will be developed, implemented, and enforced by the Publications Committee. The Publications Committee will be composed of the NANO Steering Committee. The P&P policies and procedures will be developed as part of the MOP and communicated to all participating study sites. All investigators will be encouraged to participate in opportunities for presentation and publications. These policies will provide for optimizing the use of valuable data collected by the study and provide an additional non-financial incentive for participating investigators.

## Discussion

We encountered several study design challenges during the creation and initiation of the NANO trial. These challenges revolved around key aspects of the trial: identifying the appropriate target population, our antenatal and postnatal consent process, and the selection and use of antibiotics. Decisions regarding these aspects of the trial incorporated current literature and previous research efforts and were based on discussions between the CCC, Steering Committee, and participating clinical sites.

During the initial discussions for the NANO trial in 2018, the study’s target population consisted of infants at low risk for EOS, including those born for maternal indications without preterm premature rupture of membranes (PPROM) or preterm labor (PTL). At that time, feedback from potential study sites indicated that these were the only infants that neonatologists would consider managing without empiric treatment for EOS. However, over the course of just a few years, antibiotic practice patterns in the NICU shifted and many potential study sites reported that they no longer administered EA on a routine basis to infants delivered prematurely for maternal indications. This has become common practice among neonatologists following recommendations from the Committee on Fetus and Newborn, and similarly it remains common practice among neonatologists to administer EA to infants at the other end of the risk spectrum—infants at high risk for EOS due to the presence of maternal intrapartum fever or intraamniotic infection [5]. Accordingly, we developed inclusion and exclusion criteria to define our population of interest, for whom neonatologists are likely to retain equipoise regarding the decision to treat or not treat empirically for EOS. Common examples of this patient population are infants born prematurely to mothers with PPROM and/or PTL but without evidence of intraamniotic infection as defined by the American College of Obstetricians and Gynecologists (ACOG) [56].

Given the unpredictable nature of preterm delivery and short enrollment window, identification and enrollment of mother and infant pairs will be a rate-limiting step for execution of the NANO trial. There are advantages and disadvantages to antenatal and postnatal strategies for approaching potential maternal participants. With antenatal enrollments, it is far simpler after delivery to administer study drug within the first 6 h of life to newborns that meet criteria for study participation. However, mothers presenting in preterm labor may not be approachable prior to delivery by study coordinators and physician investigators. With postnatal enrollments, coordinators and investigators can know with certainty whether mothers fit study criteria and concomitantly can assess infant eligibility. Nevertheless, it may be difficult to find the opportunity to review details of the trial with parents in the postpartum period. It also may not be possible to obtain postnatal consent and randomize a newborn within the first 4 h of life as detailed in the study protocol. The CCC will assist each of the study sites in developing consent processes that tailor to their specific neonatal and obstetric practices. Based on site feedback, we believe that there will be a role for both antenatal and postnatal consent to allow accrual of 802 infant participants.

We sought to respect clinician and site practices and ensure patient safety, while preserving methodologic rigor and blinding. First, we discussed choice of EA type and duration with sites. Issues included the use of gentamicin and variable EA administration patterns. Similar to practices documented in current literature, most institutions utilized ampicillin and gentamicin given their known efficacy toward microbes associated with EOS in preterm neonates [34]. After discussions regarding the benefits and risks to using gentamicin versus cephalosporins, including the risk of unblinding with gentamicin level monitoring, sites agreed to the use of ampicillin and gentamicin to infants who are allocated to receive empiric antibiotics. We elected to write the study protocol such that sites may utilize previously approved institution-specific guidelines for antibiotic administration with the assistance of investigational pharmacists. This flexibility in study drug administration will allow for ease of implementation of the study protocol at the various sites. Sites did agree to the discontinuation of study drugs following a period of 36–48 h, given recent observational data demonstrating the safety of discontinuing empiric antibiotic therapies 36–48 h following initial administration in those infants without positive cultures and without clinical evidence of EOS [23, 43, 57, 58]. Both of these features allow a uniform protocol with local customization and will preserve blinding.

Second, we crafted a “rescue antibiotics” plan to ensure patient safety while minimizing threats to blinding. Neonatologists may choose to administer “rescue antibiotics” if clinical conditions change and they become uncomfortable with the possibility that a given study subject may have been allocated to the placebo arm of the study. This aspect of the study protocol will ensure the safety of infants by guaranteeing that, regardless of initial treatment allocation, subjects will receive ampicillin and gentamicin if desired by treating providers. Providers may also choose to discontinue study drug and prescribe other antibiotics as indicated if they believe the infant is not appropriately covered by ampicillin or gentamicin. To ensure that providers continue to remain blinded to participant’s initial treatment allocation, investigational pharmacists will assist with the formulation and delivery of “rescue antibiotics”. By maintaining our double-blinded design and preventing investigators from knowledge of infant treatment allocation, we hope to prevent the incorporation of observer bias into our final analyses while also providing appropriate clinical care to participants. Following the cessation of the 36–72-h study period, neonatologists may choose to prescribe additional antibiotic therapies as they see fit.

In summary, the NICU Antibiotics and Outcomes Trial is the first multicenter, placebo-controlled, double-blinded, randomized controlled trial to assess whether empiric antibiotic use in the first hours of life increases or decreases the rate of adverse outcomes in ELBW preterm infants. Results of the trial may demonstrate that EA increases the composite incidence of late-onset sepsis, necrotizing enterocolitis, and/or mortality. If the incidence of the primary outcome is found to be higher in infants who have received antibiotic therapies, then empiric use of antibiotics in NICUs in this population should be reconsidered. Further studies may be designed to ascertain the long-term sequelae of early empiric antibiotic administration including their effect on growth, cognitive development, and development of other childhood disease states.

## Trial status

Protocol version 5/September 2020

Recruitment start: September 2020

Recruitment end: Spring 2024

## Supplementary Information

**Additional file 1: Figure 1.** Maternal and infant participant timeline. Description of data: NANO intervention timeline for maternal and infant participants.

**Additional file 2.**

**Additional file 3.**

**Additional file 4.**

## Data Availability

The NANO Steering Committee and CCC will have access to the final trial dataset.

## Abbreviations

AE: Adverse event
ANCOM-BC: Analysis of Composition Of Microbiomes with Bias Correction
CACE: Complier average causal effect
CDW: Clinical Data Warehouse
CCC: Clinical Coordinating Center
DCC: Data Coordinating Center
DSMB: Data and Safety Monitoring Board
EA: Empiric antibiotics
ELBW: Extremely-low-birthweight infants
EOS: Early-onset sepsis
ESBL: Extended spectrum beta-lactamase
GBS: Group B Streptococcus
GEE: Generalized estimating equations
HHS: Health and Human Services
IDS: Investigational drug service
ITT: Intent-to-treat
IND: Investigational new drug
IRB: Institutional Review Board
LOS: Late-onset sepsis
MAR: Missing-at-random
MOP: Manual of Procedures
NEC: Necrotizing enterocolitis
NICU: Neonatal intensive care unit
NIH: National Institutes of Health
PERMANOVA: Permutational multivariate analysis of variance
ROM: Rupture of membranes
SAE: Serious adverse event
