# Supplementary figures and images for: The NICU Antibiotics and Outcomes (NANO) trial: a randomized multicenter clinical trial assessing empiric antibiotics and clinical outcomes in newborn preterm infants
Source: Trials. 2022 May 23;23:428. doi: 10.1186/s13063-022-06352-3 (PMC9125935; doi:10.1186/s13063-022-06352-3)

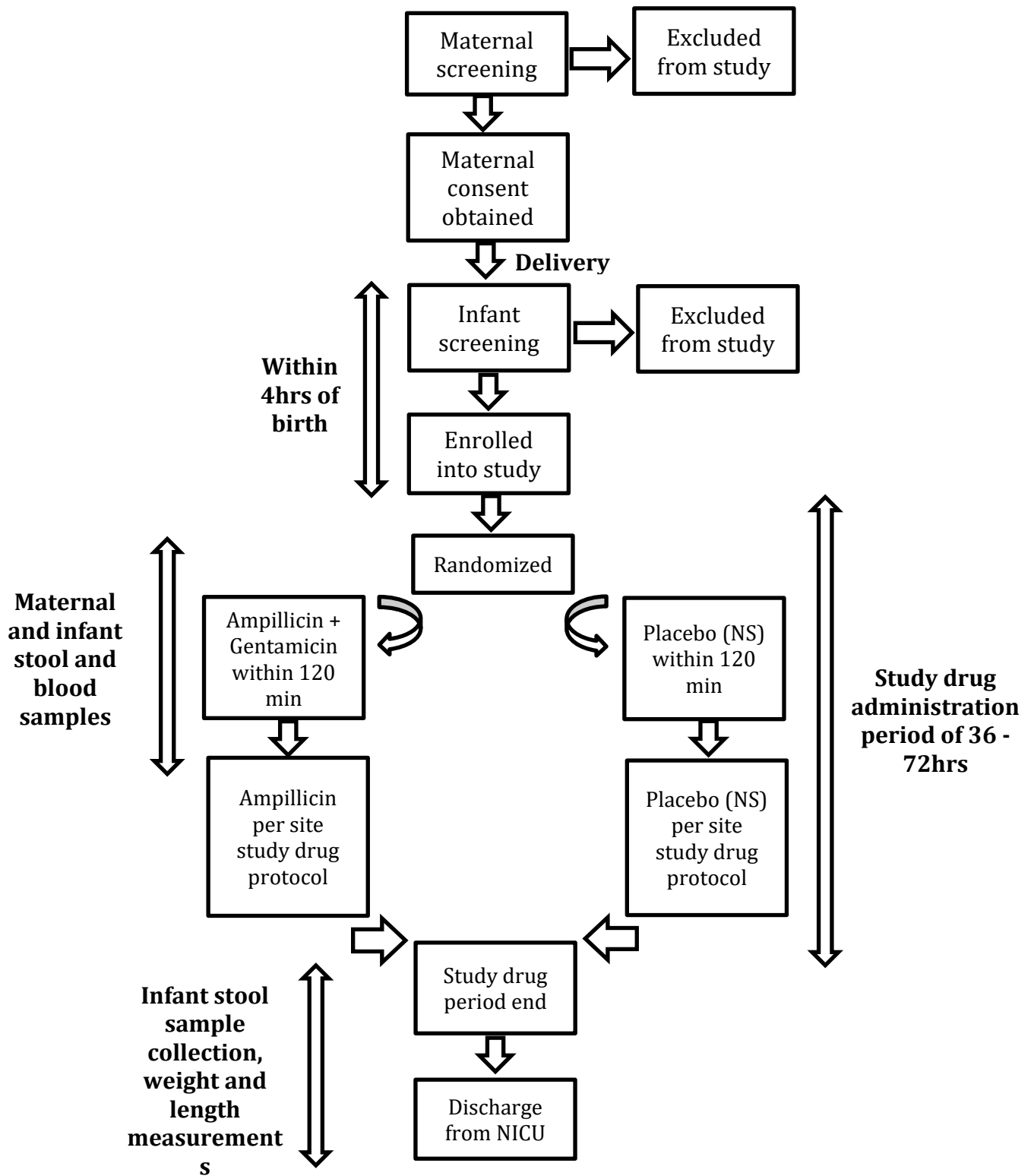

Supplement: Supplementary file 1 — Additional file 1: Figure 1. Maternal and infant participant timeline. Description of data: NANO intervention timeline for maternal and infant participants. [file 13063_2022_6352_MOESM1_ESM.pdf]
