# Supplementary material for: The NICU Antibiotics and Outcomes (NANO) trial: a randomized multicenter clinical trial assessing empiric antibiotics and clinical outcomes in newborn preterm infants
Source: Trials. 2022 May 23;23:428. doi: 10.1186/s13063-022-06352-3 (PMC9125935; doi:10.1186/s13063-022-06352-3)
Supplement: Supplementary file 2 — Additional file 2. [file 13063_2022_6352_MOESM2_ESM.docx]

THE NICU ANTIBIOTICS AND OUTCOMES TRIAL (NANO)

This page summarizes key details about the NANO trial. We hope that this information will help you decide whether you would like to enroll yourself and your newborn baby in this study. More detailed information is provided in the rest of this document. If you have questions, please be sure to ask a member of the research team.

You are being asked whether you would like to enroll yourself and your newborn baby in a clinical trial, a type of research study. Being in a clinical trial may offer some benefits and/or pose some risks compared to regular medical care.

If you decide not to take part in the clinical trial, your baby will receive regular medical care for his/her hospitalization in the NICU at [INSERT LOCAL SITE NAME]. The standard of care for extremely low birthweight neonates is usually to receive antibiotics for 36-72 hours after delivery until the results from blood cultures that were drawn at the time of delivery are available.

The purpose of this clinical trial is to:

- determine if it is safe to **not** give newborn premature infants like yours (babies that do **not** have signs of infection) antibiotics in the first 48 hours of life.
- compare health outcomes (e.g., infections, poor growth, etc.) of infants who do receive antibiotics after delivery versus infants who do not receive antibiotics after delivery
- collect samples (infant stool and blood and maternal vaginal, rectal and stool) that will be used to study the relationship between antibiotics and the development of your baby’s gut microbiome (the types of bacteria, viruses and fungus that are present in your baby’s intestines).

If you decide to participate, your baby will be randomly assigned to receive 1-2 days of either antibiotics or a normal saline solution after he/she is admitted to the NICU. The nurses will collect weekly stool samples from your baby’s dirty diapers until eight weeks of life and then 1 sample will be collected monthly until your baby is discharged from the NICU. The nurses will also collect a sample of blood from your baby at the time of birth.

No researchers have ever conducted a study such as this. Therefore, we do not know if giving antibiotics increases or decreases the risks of health problems.

Possible risks of this study:

- If your baby is randomized to receive antibiotics, the possible risks are health problems that may be associated with giving antibiotics when an infection is not present. These include baby’s blood stream infections after 3 days of life, severe intestinal problems, serious long-term breathing problems and others that are listed on pages (7,8) of this consent.
- If your baby is randomized to receive a placebo (normal saline) instead of antibiotics, there is a risk if your baby has an infection in his/her bloodstream that was not expected. This risk would be a delay in antibiotic treatment for an infection that was not treated immediately after delivery, which may or may not increase the risks of complications of sepsis, which would include death and neurological impairment.

You may choose not to participate in this study and your baby will receive the standard of care as every other baby. It will not affect their care in any way.

There is no guarantee that your child will benefit from participating in this study. There is potential for some benefit with either the placebo or treatment group and that is why we are doing this study. We hope that the information learned from this study will improve health outcomes in premature infants in the future.

If you decide not to participate in this research, your choices may include:

- Your baby receiving antibiotics if recommended by his/her doctor
- Taking part in another study
- You and your baby receive routine care for a premature delivery

CONSENT TO ACT AS A PARTICIPANT IN A RESEARCH STUDY

**STUDY TITLE**: THE NICU ANTIBIOTICS AND OUTCOMES TRIAL (NANO)

**Principal Investigator (PI):** [INSERT NAME, ADDRESS, PHONE (24 HOUR)]

**Co-Investigator (Co-I):** [INSERT NAME, ADDRESS, PHONE]

**Source of Support:** National Institute of Health (NIH)

You are being asked to take part in a research study and permit your baby to participate in this same research study for premature infants born between a gestational age (GA) of 23 weeks to less than or equal to 28 weeks. A member of the research team will explain what is involved in this study and how it will affect you and your baby. This consent form describes the study procedures, the risks and benefits of participation, as well as how you and your baby’s confidentiality will be maintained. Please take your time to ask questions and feel comfortable deciding whether to allow you and your baby to participate or not. This process is called informed consent. If you decide to allow yourself and your baby to participate in this study, you will be asked to sign this form. You will be given a copy of the form and should keep this copy for your records. It has information, including important names and telephone numbers, to which you may wish to refer in the future.

**WHY IS THIS STUDY BEING DONE?**

Historically, doctors have prescribed a few days of antibiotics to most premature babies, as a small percentage of premature babies are born with a bad infection called sepsis. If blood tests prove that there is no infection, then the antibiotics are stopped. However, approximately less than 2% of premature babies have this infection. It is possible that giving antibiotics increases the risk of infection and may worsen your baby’s health. It is also possible that giving antibiotics will improve your baby’s health. The purpose of this study is to help doctors decide whether antibiotics should be used in this situation.

**HOW MANY PEOPLE WILL TAKE PART IN THE STUDY?**

About 802 premature newborns and their mothers will be enrolled in this study from [INSERT LOCAL SITE NAME] Hospital and at least 6 other large hospitals within the United States. Approximately [INSERT LOCAL PROJECTED ENROLLMENT] mother and baby pairs will be enrolled from [INSERT LOCAL SITE NAME].

**WHAT PROCEDURES WILL BE PERFORMED FOR RESEARCH PURPOSES?**

By enrolling your baby in the study, you will also authorize (give permission to) this research team to access your medical record and obtain information regarding:

- Demographics
- Information about your pregnancy and delivery
- Antibiotics given to you before, during and after delivery
- Steroids given to you prior to your delivery
- Information about your placenta

This list is meant to serve as an example and additional questions may be asked.

This research study will involve the recording of current and/or future identifiable medical information from your baby’s hospital medical record including:

- Your baby’s medical record number and date of birth
- Antibiotics that your baby received during his/her NICU admission
- What types of feedings (your breast milk, donor breast milk or formula) and extra calories (fortification) that your baby was given in the hospital
- Any medical problems that your baby may have had while they were in the hospital
- The results of any tests or procedure that your baby received while in the NICU
- Your baby’s weekly length and weight measurements that are recorded in his/her medical record

This authorization is valid for an indefinite period of time; this identifiable medical record information may be made available to members of the research team for an indefinite period of time.

***Research Procedures:***

**Mother’s Procedures**:

1. When you deliver your baby, the doctor may obtain a vaginal sample from you at the time of delivery. A sterile swab (q-tip) will be gently inserted into the lower part of the vagina. The doctor will insert this swab without using a speculum (medical instrument that can be used for a vaginal exam). The swab will be rotated for about 10 seconds and then removed. We will analyze the germs (bacteria, viruses and fungii) present within this sample and compare it with the germs (bacteria, viruses and fungii) found in your baby stool samples.
2. When you deliver your baby, the doctor may obtain a rectal sample from you at the time of delivery. A sterile swab (q-tip) will be gently inserted to a depth of 4cm into the rectum. The swab will be rotated 3 times and then removed. We will analyze the germs (bacteria, viruses and fungi) present within this sample to help us understand your microbiome (genetic material that lives on and inside your body).
3. If you and your baby are enrolled after you deliver your doctors will not collect a vaginal or rectal sample.
4. You may be asked to collect a maternal stool sample within the first week after you deliver to provide to the study team. We will provide you with a hospital toilet liner, a pair of gloves, a wooden stick and a container to collect your stool sample. You can give the sample to the nurse taking care of your baby. This sample will be de-identified and put in the freezer in the research room.
5. We would like you to answer some questions about you and your general health. These questions will include questions about your diet, medications you were taking during your pregnancy, your education and ethnicity. If you chose not to answer these questions because you are uncomfortable, you and your baby can still take part in this study.

**For your baby:**

1. Randomization: One group of infants in this study will receive standard antibiotic therapy (ampicillin and gentamicin). The other group will receive an inactive substance (normal saline) instead of antibiotics. This normal saline is also called a placebo. Your baby will be randomly assigned to one of these two research groups. This means that your baby will have a 50/50 chance (like flipping a coin) of being assigned to either the antibiotic group or the placebo group. Neither the researchers or you will be able to choose what group your baby is in.

If you deliver more than one baby and they are each enrolled in this study, each of the babies that you deliver will be randomized to the same study treatment group.

1. Blinding: Neither you, your baby’s doctor, or the people taking care of your baby will know if your baby is receiving antibiotics or placebo (normal saline). This is called “blinding”. It is done to make sure that the observations and results are not affected by bias when the treatment group is known.

Your baby will only be permitted to participate if his/her doctor believes there are no signs of an infection. After your baby is born, the doctors taking care of your baby will closely monitor his/her vital signs (heart rate, breathing and temperature) and laboratory results. During this time, if your baby’s doctors identify signs of an infection, then your baby will receive antibiotics regardless of whether antibiotics or placebo were given to your baby in the first 2 days. Sample collection will continue.

**Participation in this trial will in no way affect any other part of your baby’s care.**

1. Stool collection: Spontaneously passed stool (feces) samples will be collected by your baby’s nurses from his/her dirty diapers. One to two samples will be collected for the first eight weeks after your baby is born and then once a month until your baby’s discharge from the NICU. These samples will be used to study the germs in your baby’s stool samples, how they change over time and to identify chemicals produced by the bacteria and viruses within the stool. The samples will be labeled only with a code number so that nobody outside of the [INSERT LOCAL SITE NAME] will be able to link a sample to your baby’s name. The samples may be sent outside of [INSERT LOCAL SITE NAME] for analysis but nobody outside of the [INSERT LOCAL SITE NAME] study team will be able to link samples to your baby’s name.
2. Blood draw: A blood draw of 0.3 to 0.4 mL will be taken to analyze your baby’s genes. will be taken.The blood draw will coordinate with clinical blood draws so as to minimize the number of times an indwelling line is accessed or to reduce the possibility of bruising by performing the heelstick After the sample is collected, it will be frozen for shipment. The samples will be labeled only with a code number so that nobody outside of the [INSERT LOCAL NAME] will be able to link a sample to your baby’s name. The samples may be sent outside of the [INSERT LOCAL SITE NAME] for analysis but nobody outside of the [INSERT LOCAL SITE TEAM] study team will be able to link samples to your baby’s name.
3. If your baby is transferred to [INSERT LOCAL SITE NAME], the nurses there will continue to collect stool samples as described above until your baby’s discharge. We will continue to review your baby’s medical record as described above while they are at [INSERT LOCAL SITE NAME].

In addition to several different types of bacteria, your baby’s stool samples will also contain some genetic material, proteins and other compounds (metabolites) that are produced by his/her body. We will study what is in your baby’s intestinal tract and how it relates to the genetic material and proteins that are produced by the bacteria. As part of this research, we may send genetic material and/or a sample of your baby’s stool to researchers at other institutions. There is a possibility that future genetic testing may include whole genome sequencing (WGS). These researchers will not receive any information about your baby, and the samples that we send to these researchers will be de-identified, which means they will have no way of knowing which samples came from which babies.

You will not be informed of the genetic information that may be received from your samples.

**HOW LONG WILL I BE IN THE STUDY?**

If you provide consent to participate in this study, you and/or your baby will remain in this study until your baby is discharged from [INSERT LOCAL SITE NAME]. Should we receive additional resources, we may contact you in the future for a follow-up study.

**WHAT ARE THE RISKS OF THE STUDY?**

- Randomization – Your baby will be assigned to a study group shortly after delivery (to receive antibiotics or not receive antibiotics). Although giving antibiotics is standard of care for most premature babies, it is possible that giving antibiotics increases the risk of infection and may worsen your baby’s health. It is also possible that giving antibiotics will improve your baby’s health. Finally, it is also possible that giving antibiotics makes no difference at all. The purpose of this study is to help doctors decide whether antibiotics should be used in this situation.
- Placebo – If your baby is assigned to the placebo group, your baby will receive normal saline instead of antibiotics. Normal saline is made up of water and salt and has no active substances. Normal saline is commonly given to infants to treat dehydration and has no known side effects.
- Ampicillin and gentamicin are usually given as standard of care. Since your baby may receive these antibiotics through randomization for this study, the risks of their administration may include;

skin rashes, hives, mild gastrointestinal upset with changes in stool. Temporary changes in kidney function that regulate electrolytes (sodium, calcium and magnesium) that go away after the drugs have been stopped and rarely hearing losses. These side effects are usually seen when high doses of the antibiotics have been ordered or the drugs are given for an extended period of time.

- Confidentiality – There is also a potential risk of loss of privacy when involved in a research study. The study staff will make every effort to keep you and your baby’s information private. All samples obtained from you and/or your baby will be identified with a study number, not names or medical record numbers. The code that links this number with you and/or your baby will remain in the research office at [INSERT LOCAL SITE NAME] in a password protected electronic file accessible to only the research coordinator or a person chosen the by the PI or Co-I.
- If we would use any of the stored samples for genetic research, the samples would be de-identified and no information would be put into your baby’s medical record.
- **Genetic Testing –** The risks associated with gene studies include the potential for a breach of confidentiality which could affect future insurability, employability, or reproduction plans, or have a negative impact on family relationships and/or result in paternity suits or stigmatization.
- **To further safeguard your privacy, genetic information that may be obtained in this study will not be placed in your baby's medical record**. Therefore, this study will not affect your baby’s future employment or his/her health insurance coverage. A Federal law, called the Genetic Information Nondiscrimination Act (GINA), generally makes it illegal for health insurance companies and group health plans to use genetic information in making decisions regarding eligibility or premiums. GINA also makes it illegal for employers with 15 or more employees to use your genetic information when making decisions regarding hiring, promoting, firing, or setting the terms of employment. This new Federal law does not protect you against genetic discrimination by companies that sell life, disability, or long-term care insurance.

**ARE THERE ANY BENEFITS TO TAKING PART IN THE STUDY?**

This research study may offer a potential benefit toinfants randomized to the placebo group, as this group may have less health problems and infections than infants that receive antibiotics right after delivery; however, there is no guarantee that your child will benefit from participating in this study . We hope that the information learned from this study will improve health outcomes inpremature infants in the future.

**ADVANTAGES** AND **DISADVANTAGES** OF PARTICIPATION IN THE NANO TRIAL

| **Treatment Arm 1: Assigned to receive antibiotics for at least 2 days after delivery** | | |
| --- | --- | --- |
| Possible advantages | - We estimate that 1 or 2 of every 100 study participants will be born WITH a blood stream infection that was not immediately recognized. These 1 or 2 babies will receive earlier antibiotic treatment if they are in this treatment group. It is possible that this earlier treatment is beneficial in babies born without signs of infection. | |
| Possible disadvantages | - Studies suggest that the following major medical problems may occur in premature infants that receive antibiotics at birth when they do not demonstrate signs of a blood stream infection: | |
|  | - Blood stream infections after 3 days of life - Side effects from the antibiotics (ampicillin and gentamicin) that include; skin rashes, hives, mild gastrointestinal upset with changes in stool, temporary changes in kidney function and rarely hearing losses. - Severe intestinal problems (e.g. Necrotizing enterocolitis) - Serious long-term breathing problems - Serious long-term eye problems - Abnormal brain development - Death during the NICU hospitalization | |
|  |  |  |
| **Treatment Arm 2: Assigned to receive saline solution (placebo) for 2 days after delivery** | | |
| Possible advantages | - If our research finds that the above medical problems are more common for infants that receive antibiotics at birth, then infants assigned to this treatment group can be expected to have fewer major medical problems. - Exposure to antibiotics can promote the growth of harmful bacteria that are more difficult to kill with antibiotics. This is called antibiotic resistance. Infants assigned to this treatment group may have less types of bacteria in their gut or lungs that are antibiotic resistant. - Studies suggest that infants who are born without a blood stream infection and do not receive antibiotics may grow better | |
| Possible disadvantages | - The risks of receiving placebo rather than antibiotics have not previously been studied and therefore unproven. We estimate that about 1 or 2 of every 100 study participants will be born WITH a blood stream infection that is not immediately known. It is possible that babies randomized to placebo could suffer from a delay in starting antibiotic coverage if they in are one of the babies that have an infection not immediately known in the first few hours of life. Such a delay may or may not increase the risks of complications of infection, which may include death or neurologic impairment. If your baby would be randomized to placebo, they will not receive antibiotics until laboratory results or the baby’s clinical condition suggests that an infection might be present. | |

**RESEARCH RELATED INJURY**

INSERT LOCAL COMPENSATION FOR INJURY LANGUAGE.

**WHAT ARE THE COSTS?**

There will be no costs to you, your family, or your insurance carrier resulting from participation in this study.

Usual medical care costs include all services that are considered medically necessary for your baby’s care during his/her hospitalization. The cost of this usual, ongoing medical care will be the responsibility of you or your insurance and may include deductibles and co-payments. Similarly, this care will be subject to all the same requirements and restrictions of your insurance.

**WILL I BE PAID FOR MY PARTICIPATION?**

There is no reimbursement (payment) for participating in this study.

You/your baby’s information and specimens used in this research study may contribute to a new discovery or treatment.  In some instances, these discoveries or treatments may be of commercial value and may be sold, patented, or licensed by the investigators and the [INSERT LOCAL SITE NAME] for use in other research or the development of new products.  You will not retain any property rights, nor will you share in any money that the investigators, the [INSERT LOCAL SITE NAME], or their agents may realize.

**WHAT ABOUT CONFIDENTIALITY?**

Study records that identify you and your baby will be kept confidential. Paper records and electronic records with confidential information will be stored in locked rooms and electronic records will be password protected. These records will be accessible only by study representatives.

A notation that you are taking part in this research study may be made in your electronic medical record. A copy of this signed consent form will also be added your/your child’s medical record information. Information from the research that relates to your general medical care may be included in the record (for example, list of allergies, results of standard blood tests done at the hospital labs).

The data collected in this study will be used for the purpose described in the form. By signing this form, you are allowing the research team access to your baby’s medical records and your medical records, which include Protected Health Information. Protected Health Information (PHI) consists of any health information that is collected about you or your baby, which could include your medical history or your baby’s medical history. The research team includes the individuals listed on this consent form and other personnel involved in this study at [INSERT LOCAL SITE NAME].

In addition to the investigators listed on the first page of this authorization (consent) form and their research staff, the following individuals will or may have access to identifiable information (which may include your identifiable medical information) related to you and your baby’s participation in this research study:

- Authorized representatives of the study sponsor (NIH) and the University of Pittsburgh Office of Research Protections may review your identifiable research information (which may include your identifiable medical information) for the purpose of monitoring the appropriate conduct of this research study.
- The de-identified research information that is obtained about you and your baby may be shared with investigators that are participating in this study from other hospitals.
- [INSERT ADDITIONAL LOCAL ENTITIES THAT MAY HAVE ACCESS TO PHI]

We will protect the privacy and confidentiality of your and your baby’s records, as described in this document, but cannot guarantee the confidentiality of the research records, including information obtained from the medical record, once your and your baby’s personal information is disclosed to others outside of [INSERT LOCAL SITE NAME].

To help us protect your privacy, we have a Certificate of Confidentiality from the National Institutes of Health. The researchers can use this Certificate to legally refuse to disclose information that may identify you in any federal, state, or local civil, criminal, administrative, legislative, or other proceedings, for example, if there is a court subpoena. The researchers will use the Certificate to resist any demands for information that would identify you, except as explained below.

The Certificate cannot be used to resist a demand for information from personnel of the United States Government that is used for auditing or evaluation of federally‐funded projects or for information that must be disclosed to meet the requirements of the federal Food and Drug Administration (FDA).

You should understand that a Certificate of Confidentiality does not prevent you or a member of your family from voluntarily releasing information about yourself or your involvement in this research. If an insurer, employer, or other person obtains your written consent to receive research information, then the researchers may not use the Certificate to withhold that information.

The Certificate of Confidentiality will not be used to prevent disclosure to state or local authorities to prevent serious harm to yourself, children or others, for example in cases of baby abuse or neglect. If the researchers learn that you or someone with whom you are involved is in serious danger of harm, they will need to inform the appropriate agencies as required by [INSERT LOCAL STATE] law.

The PI is not required to release to you research information that is not part of your baby’s medical record. The information and stool samples will be kept indefinitely. This is because information that is collected for research purposes continues to be used and analyzed for many years and it is not possible to determine when this will be complete. The data, samples and genetic data generated from samples may be shared with other researchers and with federal repositories, in a de-identified manner (without identifiers).

Information from this study may be used in medical publications or presentations or may be deposited into publicly accessible databases of genetic information. Your name and your baby’s name and other identifying information will be removed before this information is used.

A description of this clinical research study is available on <http://www.ClinicalTrials.gov>, as required by U.S. law. This web site will not include information that can identify you. At most, the web site will include a summary of the results. You can search the web site at any time.

**WHAT ARE MY RIGHTS AS A PARTICIPANT?**

Taking part in this study is voluntary. If you choose not to participate in this study, your care and your baby’s care at the [INSERT LOCAL SITE NAME] will not be affected. You may withdraw, at any time, consent for you and your baby’s participation in this research study, including your authorization to allow the research team to review your/your baby’s medical records. If you do so, you and your baby will no longer be permitted to participate in this study. Any information obtained from you/your baby up to that point will continue to be used by the research team. Leaving the study will not affect your care at the [INSERT LOCAL SITE NAME].

If you choose for you and your baby to no longer be in the study, and you do not want any of your future health information to be used, you must inform the PI in writing at the address on the first page.

**FOR HOW LONG WILL THE INVESTIGATORS BE PERMITTED TO USE AND DISCLOSE IDENTIFIABLE INFORMATION RELATED TO THIS RESEARCH STUDY?**

[INSERT LOCAL DATA RENTENTION LANGUAGE]

**VOLUNTARY CONSENT/ PARENTAL PERMISSION**
The above information has been explained to me and all of my current questions have been answered. I understand that I am encouraged to ask questions about any aspect of this research study during the course of this study, and that such future questions will be answered by a qualified individual or by the investigator(s) listed on the first page of this consent document at the telephone number(s) given. I understand that I may always request that my questions, concerns or complaints be addressed by a listed investigator.

I understand that I may contact the Human Subjects Protection Advocate of the IRB Office, University of Pittsburgh (1-866-212-2668) to discuss problems, concerns, and questions; obtain information; offer input; or discuss situations in the event that the research team is unavailable.

By signing this form, I agree for me and my baby to participate in this research study and authorize the use of my and my baby's medical record information for the purpose described above. A copy of this consent form will be given to me/my baby.

I understand that, as a minor (age less than 18 years), my baby is not permitted to participate in this research study without my consent. Therefore, by signing this form, I give my consent for his/her participation in this research
study.

___________________________________________

Printed Name of Mother-Subject Signature of Mother

_______________________________________ ___________________________________________

Printed Name of Baby-Subject Date/Time

**CERTIFICATION of INFORMED CONSENT**
I certify that I have explained the nature and purpose of this research study to the above-named individual(s), and I have discussed the potential benefits and possible risks of study participation. Any questions the individual(s) have about this study have been answered, and we will always be available to address future questions as they arise. I further certify that no research component of this protocol was begun until after this consent form was signed.

Printed Name of Person Obtaining Consent Role in Research Study

Signature of Person Obtaining Consent Date/Time
