# Supplementary material for: The NICU Antibiotics and Outcomes (NANO) trial: a randomized multicenter clinical trial assessing empiric antibiotics and clinical outcomes in newborn preterm infants
Source: Trials. 2022 May 23;23:428. doi: 10.1186/s13063-022-06352-3 (PMC9125935; doi:10.1186/s13063-022-06352-3)
Supplement: Supplementary file 3 — Additional file 3. [file 13063_2022_6352_MOESM3_ESM.docx]

**PROTOCOL TITLE:**

The NICU Antibiotics and Outcomes Trial (NANO)

**PRINCIPAL INVESTIGATOR:**

**Michael J. Morowitz, MD, FACS**

Associate Professor of Surgery

University of Pittsburgh School of Medicine

Attending Pediatric Surgeon, Division of Pediatric General and Thoracic Surgery

Children’s Hospital of Pittsburgh of UPMC

Rangos Research Center 6^th^ Floor

4401 Penn Avenue

Pittsburgh, PA 15224

(412) 692-5976

[Michael.morowitz@chp.edu](mailto:Michael.morowitz@chp.edu)

**VERSION NUMBER/DATE:**

Version 5/September 2020

**REVISION HISTORY**

| **Revision #** | **Version Date** | **Summary of Changes** | **Consent Change?** |
| --- | --- | --- | --- |
| 1 | 1/30/2020 | Updated statistical analysis, specified infant blood draw collected for genetic analysis, changed study drug dosing and duration, specified how patients with suspected infection will be treated | Yes |
| 2 | 3/20/2020 | Updated rescue antibiotic language and statistical analysis plan | No |
| 3 | 4/21/2020 | Updated Section 8.0 and updated languages that recruitment and consent documents will be available in | No |
| 4 | 6/23/2020 | Updated screening log information and added an example in rescue antibiotic scenario, updated DCF table, updated timeline for maternal stool collection | No |
|  |  |  |  |

Table of Contents

[1.0 Study Summary 3](#_Toc496162129)

[2.0 Objectives 4](#_Toc496162130)

[3.0 Background 4](#_Toc496162131)

[4.0 Study Endpoints 5](#_Toc496162132)

[5.0 Study Intervention/Investigational Agent 5](#_Toc496162133)

[6.0 Procedures Involved 6](#_Toc496162134)

[7.0 Data and Specimen Banking 11](#_Toc496162135)

[8.0 Sharing of Results with Subjects 12](#_Toc496162136)

[9.0 Study Timelines 12](#_Toc496162137)

[10.0 Inclusion and Exclusion Criteria 12](#_Toc496162138)

[11.0 Local Number of Subjects 14](#_Toc496162140)

[12.0 Recruitment Methods 14](#_Toc496162141)

[13.0 Withdrawal of Subjects 14](#_Toc496162142)

[14.0 Risks to Subjects 15](#_Toc496162143)

[15.0 Potential Benefits to Subjects 16](#_Toc496162144)

[16.0 Data Management and Confidentiality 16](#_Toc496162145)

[17.0 Provisions to Monitor the Data to Ensure the Safety of Subjects 22](#_Toc496162146)

[18.0 Provisions to Protect the Privacy Interests of Subjects 25](#_Toc496162147)

[19.0 Compensation for Research-Related Injury 25](#_Toc496162148)

[20.0 Consent Process 26](#_Toc496162150)

[21.0 Process to Document Consent in Writing 26](#_Toc496162151)

[22.0 Setting 26](#_Toc496162152)

[23.0 Resources Available 27](#_Toc496162153)

[24.0 Multi-Site Research 27](#_Toc496162154)

# Study Summary

| **Study Title** | The NICU Antibiotics and Outcomes Trial (NANO) |
| --- | --- |
| **Study Design** | Randomized, placebo controlled, double blinded multicenter trial |
| **Primary Objective** | To compare the incidence of late onset sepsis (LOS), necrotizing enterocolitis (NEC), and death in premature infants ≤28 weeks gestation randomized to receive empiric antibiotics (EA) or placebo at birth. |
| **Secondary Objective(s)** | To compare early patterns of gut microbial colonization in premature infants ≤28 weeks gestation randomized to receive empiric antibiotics (EA) or placebo at birth. |
| **Research Intervention(s)/ Investigational Agent(s)** | The intervention consists of administering ampicillin and gentamicin at site approved dosing guidelines OR a volume matched equivalent of normal saline. |
| **IND/IDE #** | IND Exempt (PIND 143353) |
| **Study Population** | Newborn infants born at participating study sites with gestational age of 23-28 weeks |
| **Sample Size** | 802 |
| **Study Duration for individual participants** | Duration of index hospitalization |
| **Study Specific Abbreviations/ Definitions** | **ELBW-** Extremely Low Birth Weight Infants  **LOS**- Late Onset Sepsis  **NEC**- Necrotizing Enterocolitis: A disease that affects mostly the intestine of premature infants. The wall of the intestine is invaded by bacteria, which can cause local infection and inflammation that can destroy the intestinal wall.  **EA**- Empiric Antibiotics  **EOS-** Early Onset Sepsis  **NICU-** Neonatal Intensive Care Unit  **NIH-** National Institutes of Health  **AE-** Adverse Event  **SAE-** Serious Adverse Event  **IND**- Investigational New drug  **IDS**- Investigational Drug service  **IRB-** Institutional Review Board  **HHS-** Health and Human Services  **DSMB-** Data and Safety Monitoring Board  **MOP-** Manual of Procedures  **DCC-** Data Coordinating Center |

# Objectives and Aims

# The goal of the NANO Trial is to test the hypothesis that the rate of adverse outcomes is higher in ELBW infants receiving EA compared to infants receiving placebo. We have three aims:

# Aim 1. To test the hypothesis that the composite incidence of LOS, NEC, and/or death is significantly different in infants that receive EA and infants that receive placebo.

Aim 2. To test the hypothesis that fecal samples in the first month of life from infants receiving EA will contain lower diversity, higher abundance of pathogens, and lower abundance of commensal anaerobes than fecal samples from infants receiving placebo.

Aim 3**.** (Exploratory) To identify microbial taxa associated with delayed or accelerated somatic growth (weekly weight and length z-scores) during the first month of life in infants receiving placebo or EA.

# Background

# Early onset sepsis (EOS) is a rare but morbid bloodstream infection that can occur in newborn infants during the first 3 days of life. Timely diagnosis of EOS is challenging in extremely low birthweight infants since there are no accepted biomarkers for the disease and because the clinical signs of EOS (e.g. respiratory failure) can overlap with common non-infectious physiologic derangements in preterm infants. As a result, the standard of care in many NICUs is to administer empiric antibiotics (EA) to preterm infants during the first 2-7 days of life or until a workup for EOS is complete^9–11^. Population-based studies have consistently shown that 80-90% of extremely low birthweight (ELBW) infants receive EA despite an EOS incidence of 2% or less^12–14^.

# It is increasingly recognized that adverse effects of antibiotics extend beyond the well-recognized problem of antibiotic resistance to include unintended clinical consequences related to eradication of the human microbiota. Several recent studies in preterm infants have identified associations between early antibiotic exposure and adverse events including necrotizing enterocolitis (NEC) and late onset sepsis (LOS)^15–24^. For these reasons, antibiotic stewardship initiatives have been widely adopted in NICUs and indeed they have successfully reduced the length of EA therapy – however nearly all ELBW infants continue to receive at least a short course of EA at birth. It is generally accepted that published guidelines on the subject are outdated^25^, and therefore it is not surprising that EA practice patterns (e.g. length of therapy) vary wildly among providers and across centers^12,26–28^.

Our multidisciplinary team has more than 10 years of experience monitoring temporal changes in the gut microbiota of preterm infants^32–44^. In previous studies, we have documented that EA exposure is associated with unambiguous changes in the microbiota favoring growth of pathogens^10–23^. Our preliminary data supports published studies demonstrating a dose-dependent relationship between EA and a composite outcome of LOS, NEC, or mortality^45^. We and others have also described associations between antibiotics, gut bacterial colonization patterns, and infant growth^46,47^.

Completion of this trial may position us to conclude that EA therapy **worsens** outcomes or that EA **improves** outcomes in premature infants. If the former is true, results could rapidly be translated into a decrease in antibiotic usage in NICUs. Should we find *improved* outcomes with antibiotic administration, this trial would provide physicians with confidence to continue administering EA – a common practice at present despite a lack of high quality data. It is also possible that significant differences will **not** be observed between study arms and, depending upon the details, this result may itself be useful to care providers.

# Study Endpoints

# Primary Outcome: Composite incidence of adverse outcomes (NEC, LOS, or death during the index hospitalization).

# NEC will be defined strictly by Bell’s stage II or III criteria for moderate or advanced NEC^50^. To reduce overlap between NEC and diagnosis of spontaneous intestinal perforation, the diagnosis of NEC will only be considered in infants > 7 days of age. LOS is defined as a positive blood culture obtained after 72 hours of life and intent to treat with antibiotics for 5 days or more^195^. Death is defined as death during the index hospitalization.

Secondary Outcomes: NEC during the index hospitalization, LOS during the index hospitalization, or death during the index hospitalization.

# Study Intervention/Investigational Agent

Once maternal and infant eligibility are established, infants will be randomized into one of two different study groups: receiving conventional empiric antibiotics (ampicillin and gentamicin) or placebo while completing an evaluation for early onset sepsis. It is essential that randomization occurs within the first 4 hours of life.

All key resources for this proposal will be authenticated to enhance the reproducibility of our data, as appropriate and according to NIH policy (Notice Number: NOT-OD-17-068). Antibiotics used in this clinical trial will be commercially available pharmaceuticals sourced individually at each study site and administered to investigative pharmacy SOPs specific to each site. Lot numbers will be tracked to aid in identifying reagents that may be performing outside acceptable limits.

# Procedures Involved

# The NANO trial has been designed to study the longstanding clinical practice of empirically administering intravenous antibiotics to extremely low birthweight (ELBW) infants in the first days of life. It is an 802-subject multicenter placebo-controlled double-blinded randomized clinical trial to test the hypothesis that the incidence of adverse outcomes is higher in babies receiving EA in the first week of life compared to babies receiving placebo. We target a population of ELBW infants in whom the clinical decision to use or not use EA is currently most challenging -- infants that are clinically stable that did not have a known exposure to intraamniotic infection and were not born preterm for maternal indications.

# 6.1 Randomization and Blinding*.* For each infant born to a mother that has provided consent, eligibility will be assessed. One consent document is sufficient for both the mother and her baby. Thus, there will be a two-part screening to first determine maternal eligibility, and then to determine infant eligibility. IRB approved staff will screen infants based on inclusion/exclusion criteria. The site coordinator, or research staff, will discuss eligibility with approved staff and if the patient is eligible, input patient data into the web based system. Once data is inputted, research staff will randomize eligible families 1:1 using web-based block randomization stratified by study site to receive EA or placebo. Multiples (i.e. siblings) will be randomized to the same treatment arm. Staff will not see the treatment arm that patients are assigned to. Rather, treatment arm and patient information will be sent to each site’s investigational pharmacy, where study drug will be drawn and sent to the patient’s room. The staff member who performed randomization will see a confirmation message that randomization was successfully sent to the pharmacy. The investigational pharmacy is unblind to patient’s allocation. Participants, treating clinicians, and study staff will all be blinded to allocation. Randomization must occur within the first 4 hours of life. Outcome assessors and statistical summaries for trial monitoring will be unaware of group allocation. Unblinded data evaluation during the trial will be restricted to a designated study statistician and the DSMB. We will unblind investigators and begin analyses only after all data collection forms are completed, data queries resolved, and data are locked for analysis.

# 6.2 Study Procedure*.* The intervention consists of administering either conventional EA or placebo while completing an evaluation for early onset sepsis. Each site will source antibiotics individually. Either conventional EA or placebo must be given intravenously within 120 minutes following randomization. If EA is given, it will consist of ampicillin and gentamicin administered according to local, site-approved dosing guidelines clearly reflected in each site’s SOP. Infants randomized to receive placebo will receive volume-matched equivalents of normal saline matching the analogous schedule of ampicillin and gentamicin administration.

6.3 Rescue antibiotics. We anticipate that <<5% of study subjects will experience worsening of conditions prompting clinicians to order additional antibiotics that will be termed "rescue antibiotics." Clinicians will have complete freedom to repeat blood cultures and to order the antibiotics that they feel are most appropriate to treat the infant, with no restrictions imposed by the study protocol. This is a specific safety measure for NANO but is fully consistent with the standard of care for infants experiencing clinical deterioration. Should this occur, the treating physicians will remain blinded to the initial study drug assignment. The study pharmacist will be unblinded. Patients who have received a rescue option will continue to be followed until discharge from the NICU.

# Rescue antibiotic therapy. There are two primary scenarios -- both rare -- that may be encountered requiring rescue antibiotic therapy.

# The first scenario may occur if an Attending Neonatologist wishes to guarantee the infant actually receives ampicillin and gentamicin and not only placebo. To accomplish this, a second set of study drug orders will be placed while the original study drug orders are continued. For the second set, if the original assignment was placebo, pharmacy will prepare ampicillin and gentamicin. If the original assignment was ampicillin and gentamicin, pharmacy will prepare placebo and placebo. Sites that give ampicillin every 8 hours according to their local standard of care can elect to provide blinded active ampicillin as rescue antibiotic instead of both placebo and ampicillin in order to reduce daily drug administration burden. This would not affect the gentamicin rescue, which is only a single dose.

# The order and label for rescue antibiotics should include some rescue status indicator to avoid preparation/dispensing/administration errors. If this rescue antibiotic scenario occurs, providers will have a guarantee that the infant is receiving ampicillin and gentamicin. Providers will not know what the initial order set consisted of, in order to maintain blinding. The second set of orders should discontinue at the same time as the first round of orders. The schedule for subsequent ampicillin doses after the study period has ended (if desired) would be based on the timing of the split difference between the last study placebo and active ampicillin administrations. The schedule for subsequent gentamicin doses will be based on local standard therapeutic drug monitoring.

# The second rescue scenario will involve a decision by an Attending Neonatologist to empirically treat suspected/confirmed bacterial threats not optimally covered by ampicillin and gentamicin (e.g. penicillin for syphilis, ceftriaxone for gonorrhea, azithromycin for chlamydia, vancomycin or nafcillin for Staph, meropenem or cefepime for MDR GNR) with open label non EA antibacterials. These are ordered outside of the study and are not the responsibility of the IDS. Routine Candida prophylaxis (e.g. fluconazole) is not considered an open-label antibacterial for the purposes of this study and its use is not viewed as rescue therapy.

#

# 6.4 Early Discontinuation of EA. Study drugs can be discontinued at the discretion of the Attending Neonatologist prior to the completion of the study period, just as EA therapy might normally be discontinued outside the context of the trial. The Attending Neonatologist should ideally discuss their desire to discontinue study drugs early with the PI and research team before doing so. Potential reasons for discontinuing study drugs include:

# •The Attending wants the participant to receive rescue antibiotic therapy (see above).

# •They have decided that EA treatment is no longer necessary for clinical care (e.g. blood culture negative and no clinical concern for infection).

# Ordering and administering antibiotics after the study period is at the discretion of the Attending Neonatologist. In such cases, NICU prescribers will place new orders for these antibiotics per routine care. IDS is not responsible for these antibiotics since they are beyond the study period and the participant has completed the study drug therapy. The NICU treatment team will decide their dosing and start times. A sensible approach would be to stay on the same schedule as the study drugs. If the participant was previously converted to blinded EA, the start time can be the split difference between the two sets’ schedules. Gentamicin plasma concentration monitoring may be used to help with determining start times (see next section). Prescribers will not be unblinded to determine these start times. They should remain blinded and should not count study doses towards the total days of therapy that the baby ultimately receives.

# 6.5 Gentamicin plasma concentration monitoring. It is expected that a small percentage of participants will receive gentamicin after the study period. This will trigger site-specific procedures for routine care gentamicin therapeutic drug monitoring, the results of which will may make it possible for blinded providers and staff to discern group allocation. This is expected and unavoidable. Pharmacists who routinely perform clinical pharmacokinetics in the NICU should therefore be part of the unblinded study pharmacist team. If allowed by site practice standards, clinical PK assessments that include dosing histories may be kept with the participant’s Dispensing Log rather than in their medical chart, in order to minimize the risk of unblinding.

# 6.6 Timeliness*.* Timeliness of the study intervention is of paramount importance and will be closely monitored. To facilitate proper implementation of the study intervention, it is the job of the coordinator to inform the attending physician, nurse practitioner and/or providers that consent has been given and randomization has occurred using an online system.

# This protocol has been reviewed by the Food and Drug Administration (Department of Health and Human Services), and it was determined that an IND was not required to administer ampicillin or gentamicin as proposed for the NANO Trial.

# 6.7 Records and Data. Data to be obtained will include de-identified demographic and clinical information extracted from the medical record. Site coordinators will collect data by direct observation, chart review, and/or physician interview, and will enter data into a web-based form that minimizes burden and cost. Clinical data relevant to the study will be entered into this same database, and investigators will use only a specially assigned study number. An encoding table that links the study number to the subject’s name and medical record number within each institution will reside on a password-protected computer behind clinical firewalls.

| Maternal and infant data variables to be collected are summarized in Table 1 below. **Table 1. Variables for data collection form** | | |
| --- | --- | --- |
| **Maternal** | **Demographics** | DOB, race, highest education level. BMI |
|  | **Pregnancy history** | Number of fetus(es), number of infants born, prenatal obstetrics visit, history of spontaneous preterm birth, progesterone therapy prior to admission, GBS status |
|  | **Diagnoses during current pregnancy** (Yes/No) | Insulin-dependent diabetes, maternal hypertension, preeclampsia, antepartum hemorrhage, placenta previa, abruptio placenta, fetal growth restriction, cervical insufficiency, preterm labor, PPROM, # of days PPROM intra-amniotic infection, positive placenta culture, positive urine cultures, genital tract infection, skin/soft tissue infection, vaginal yeast infection |
|  | **Prenatal antibiotics administered within 30 days of delivery** | Antibiotic name, start date, end date, reason |
|  | **Other drugs administered during current pregnancy** | Tocolytic therapy, preoperative prophylactic antibiotics, betamethasone, antenatal corticosteroids |
| **Infant Baseline** | **Delivery** | Date, time, gestational age (weeks/days), sex, birth weight (kg), birth length (cm) |
|  | **Randomization** | Inclusion/exclusion criteria, PHI |
| **Infant Hospital Course** | **Outcomes at 1 week of age** | Culture positive EOS diagnosis   1. If Yes: causative organism   Days of antibiotics received during Week 1 of life |
|  | **Weekly** | Weight (kg), length (cm), LOS diagnosis (Yes/No), NEC diagnosis (Yes/No)antibiotic administration |
|  | **Nutrition (Days 3, 7, 14, 28, 60)** | Enteral nutrition, type of milk received, date subject reached full enteral feedings |
|  | **Hospitalization** | Hospital admission date, NICU admission date |
|  | **Discharge** | Date of discharge, diagnoses: (Grade 3 or 4 IVH, ROP, CLD, PDA), days of endotracheal intubation, positive blood cultures, positive respiratory cultures, positive urine cultures, positive cerebrospinal fluid cultures), total number of days antibiotics, total number of days of oral or IV antifungal medications, antibiotics within first 72 hours of life |
|  | **Events** | AE, SAEs UAP, protocol deviations reporting |
|  | **Antibiotics prescribed** | Antibiotic used, start and end date, reason |
|  | **Positive Cultures (>5 days of antibiotic treatment)** | Date, source of culture, isolated organism |
|  | **Study Drug** | Start date, end date, time |

# 6.6 Sample Collection.

# Infant

# 1-2 spontaneously expelled fecal samples for microbiome analyses will be obtained weekly from study subjects up until 8 weeks of life. After 8 weeks of life, 1 spontaneously expelled fecal sample will be collected monthly. If a subject is diagnosed with NEC or LOS, additional stool samples may be requested, as available. SOPs will be distributed to all sites to ensure proper collection of fecal samples.

An additional research blood sample for genetic analysis will be drawn one time and should be done at the time of clinical blood draws. However, if this blood draw is missed, it can be done in the neonate's first week of life. A volume of 0.3 to 0.4mL will be drawn in EDTA tubes and shaken well. After the sample is collected, it will be frozen for shipment. The blood draw will be performed by NICU personnel who routinely draw blood on preterm babies. It will be either the bedside nurse or the respiratory therapist depending on whether the blood is drawn from an umbilical catheter or by heelstick.

**Maternal**

# Intrapartum vaginal and rectal swabs will be collected on mother’s at sites that have infrastructure that allows them to do so. We recognize maternal collections will not be feasible at all study sites and in all cases, and therefore anticipate collecting maternal fecal and vaginal samples from 25% of mothers participating in the study. Sites that can collect these samples will be designated before enrollment of their first patient. SOPs will be distributed to all sites to ensure proper collection of these samples.

# If a vaginal and rectal swab cannot be obtained due to lack of infrastructure or personnel, a postpartum maternal fecal sample (self-collected) will be collected. If a rectal swab is missed at sites that plan on collecting maternal swabs, a fecal sample should be collected. Fecal samples must be collected within one week postpartum. Samples will be obtained exclusively for research purposes, and there will be no testing of patients beyond obtaining stool samples and recording demographic data and clinical history. An electronic database will be used to track sample collection and storage history.

6.8 Follow Up. Currently, there are no plans for follow-up but there is a possibility for funding in the future.

6.9 Consent. Sites will develop an SOP to identify women as early as possible that are admitted to participating hospitals and expected to deliver an infant at or before 28 weeks gestation. If eligible, and if the attending obstetrician provides permission, then trained research personnel will explain the research protocol and the process of informed consent. Research staff will also have the option of showing an IRB approved video to parents. This video has been created to provide a brief background about the trial, discuss the procedures involved and touch upon the risks associated with participating in this trial. This video is not required to be shown at sites but is recommended. Informed consent requires that the mother of the study subject understand the details of the study and agrees, without coercion, to participation in the study. Study representatives at each site will introduce and explain the study to the mothers and present them with the detailed consent form to read and review. It will be the responsibility of the site investigator to ensure that the mother is given full and adequate verbal and written information about the nature, purpose, benefit, and the potential risks of the study. A copy of signed informed consent document will be provided to the mother and another will be placed in the medical chart. The original document will be placed in the study files. Permission for study participation will be obtained from one parent. If there are multiples, a consent document must be obtained for each child.

# Data and Specimen Banking

#

# A single sample of blood from each study subject will be cryopreserved at The University of Pittsburgh for genotyping and/or exome sequencing at a later date. They will be housed in the CRISMA Clinical Research Biospecimen Core Laboratory at Pitt. All sites will receive shipping supplies and instructions on how to properly ship samples back to the University of Pittsburgh for proper storage.

Infant stool samples and self-collected maternal stool samples will be collected, de-identified and stored for microbiome analyses. Maternal vaginal and rectal swabs will be collected by the obstetrics team and stored for paired microbiome analyses.

# Cryopreserved patient samples will be de-identified such that no patient identifier information is accessible. A key of de-identified samples will be preserved on a secure password protected institutional server. Patient identifiers will be preserved in this protected environment until the study is completed, and then they will be de-identified.

# De-identified patient samples may be released or studied only with the express written consent of the NANO Steering Committee.

All banked biological specimens will be stored indefinitely.

# Sharing of Results with Subjects

Parents of study subjects will not be informed of results of genetic or microbiome analyses that may be performed on samples collected from their children.

# Study Timelines

The duration of study participation for each individual study subject will be identical to the duration of his/her index NICU hospitalization. Enrollment in the NANO Trial is anticipated to begin in 2020 and conclude in 2024. Primary analyses of study data are anticipated to be completed by 2025.

Major annual milestones are also listed here:

- **Year 1:** IRB approval at the coordinating center and all study sites; completion of web-based data collection form; negotiation and execution of contracts between study sites; completion of two pre-launch site visits; initial study rollout.
- **Year 2:** Study launched at all sites; 30% enrollment completed; data from first interim analysis sent to DSMB; microbiome sequencing and analysis begins.
- **Year 3:** Data from second interim analysis sent to DSMB after 60% enrollment completed.
- **Year 4:** 90% enrollment completed.
- **Year 5:** 100% enrollment completed; completion of data querying and cleaning; data lock achieved; microbiome analysis completed; submission of abstracts/manuscripts for publication; submission of final study report to NICHD.

# Inclusion and Exclusion Criteria

# Labor and delivery antenatal units will be screened for all admissions of mothers with pregnancies ≤28 weeks GA.

# 10.1 Inclusion/Exclusion Criteria.

# Participant Inclusion Criteria:

- Newborn infants born with gestational age of 23-28 weeks
- Inborn infants at participating study sites
- Mothers of babies (23-28 weeks) that were not approached prenatally may be approached within 4 hours of delivery if they are inborn and do not present with any maternal or infant exclusion criteria

# Participant Exclusion Criteria:

- Infants at low risk for EOS – born for maternal indications via caesarean section with ROM within 6 hours of delivery, no attempts to induce labor, and no concern for maternal infection
- Infants at high risk of EOS born to mothers with intrapartum fever (> 38ºC) or clinical diagnosis of chorioamnionitis (suspected or definite)
- Infants with respiratory insufficiency requiring invasive mechanical ventilation and FiO_2_ > 0.40 or non-invasive ventilation (i.e. CPAP) and FiO_2_ > 0.60 at time of randomization
- Infants with ongoing hemodynamic instability requiring vasopressors or more than one fluid bolus at time of randomization
- Clinician concern for sepsis due to physical exam findings, e.g. lethargy
- Major congenital anomalies
- Infants not anticipated to survive beyond 72 hours
- Infants who have received antibiotics prior to randomization
- Mothers that are <18 years old at time of enrollment

# 10.2 Study Restriction. As detailed in the text, study participation will be restricted to premature newborns and sample collection will be limited to the first months of life. This will enable us to test the hypothesis that EA increases the incidence of adverse outcomes after premature birth. For this reason, it is necessary to limit our study to this specific patient demographic. Prisoners and institutionalized women are excluded from the study.

10.3 Screening Log. Screening logs provide documentation of patients that are screened for study eligibility. NANO clinical sites will be instructed to maintain patient screening logs per standard practice. The CCC will provide a template for sites to use. At any time, the CCC can request copies of deidentified screening logs to track enrollment.

# Local Number of Subjects

We anticipate 40% of enrollments to occur at The University of Pittsburgh, around 325 patients, with the remainder distributed relatively even across the additional study sites.

# Recruitment Methods

# Sites will develop protocols to identify women as early as possible that are admitted to participating hospitals and expected to deliver an infant at or before 28 weeks gestation. Trained research personnel will identify and discuss informed consent with parent(s) of potential study subjects. Because variable amounts of time will have elapsed between consent and delivery, research staff will meet briefly on a regular basis with parents that have provided antenatal consent. If antenatal consent is not possible, research staff will seek postpartum consent as clinical circumstances permit. For each infant born to a mother that has provided consent, the coordinator will confirm eligibility of the infant with IRB approved staff. Thus, there will be a two-part review to first determine maternal eligibility, and then to determine infant eligibility. Only infants meeting eligibility criteria and consented for participation will be enrolled.

# Our strategy to maximize enrollment and retention of study subjects relies upon continual communication with parents and providers of study subjects. We will adopt site-specific and centralized strategies designed to optimize retention. At the time of informed consent and subsequently, we will strive to explain the details and the timeline of the study protocol as clearly as possible. We will encourage parents of study subjects to contact research coordinators and/or site PIs with questions. We will also attempt to be as flexible as possible regarding the timing of sample collections, respecting the privacy of NICU families and respecting the limited time and availability of NICU nursing staff. As often as possible, we will strive to convey to NICU providers and families that the motivation for this trial is to minimize antibiotic exposure and to improve outcomes for preterm infants. Barriers to retention of infants should be minimal as the period during which research data are being collected is limited to the index NICU hospitalization.

Labor and delivery antenatal units will be screened for all admissions of mothers admitted with pregnancies ≤28 weeks GA.

There is no reimbursement (payment) for participating in this study.

# Withdrawal of Subjects

No subject will be withdrawn from the study unless his/her parent(s) elects to withdraw the infant from further participation and notifies the study team accordingly.

For subjects that have been withdrawn from the trial, data, including samples obtained prior to the point of withdrawal can be used by the research team as originally described in the informed consent form.

# Risks to Subjects

14.1 Potential risks*.* The trial may be associated with the following risks. Mechanisms to protect against these risks are specified below and will be detailed in the study consent document.

1. Risk of not receiving EA therapy. As shown in our preliminary data, many or most ELBW infants currently receive EA after delivery. **We do not know if outcomes for these infants will be improved without antibiotics. The study is designed to answer this question.** As with any placebo controlled trial, it is possible that participants will be harmed by receiving placebo instead of receiving active treatment. In this trial, the use of a placebo is in accordance with the Declaration of Helsinki since the efficacy and safety of EA therapy have not been clearly established. Because the study targets infants that are not critically ill, we believe that the risk of withholding antibiotic therapy is low. Further, subjects receiving placebo will not be subject to additional risks of serious or irreversible harm as a result of receiving placebo rather than antibiotics, since the trial will not hinder subjects from receiving any care or interventions necessary for their protection. Accordingly, both the plan for statistical analysis and the MOP anticipate and account for the small percentage of subjects that will be converted from EA to an extended, therapeutic course of antibiotics.
2. Risk of receiving EA therapy. Studies suggest that the following major medical problems may be significantly more likely in premature infants that receive antibiotics at birth when they do not demonstrate signs of a blood stream infection: blood stream infections after 3 days of life, side effects from the antibiotics that include; skin rashes, hives, mild gastrointestinal upset with changes in stool, temporary changes in kidney function and rarely hearing loss, severe intestinal problems (i.e. Necrotizing enterocolitis), serious long-term breathing problems, serious long-term eye problems and abnormal brain development. These side effects are usually seen when high doses of the antibiotics have been ordered or the drugs were given for an extended period of time.
3. Risk from collection of fecal samples and vaginal and rectal swabs. Infant stool samples will be collected by the bedside nurse from spontaneously expelled feces discovered in infant diapers. Maternal fecal samples will be self-collected. Maternal vaginal and rectal swabs will be collected by obstetricians during delivery. There are no known risks to infants or their mothers associated with these collections.
4. Risk from collection of blood samples. If heel stick, then the added blood draw may very slightly increase the risk of bruising due to the addition time of squeezing the heel and sometimes even needing a second “prick”. If by venipuncture or through a line, then the risk is negligible since the risks are already assumed by the clinical lab draw.
5. Risk of loss of confidentiality. There is a risk of breach of confidentiality related to the demographic and clinical data that has been recorded. In order to minimize this risk, all records pertaining to subject identifiable data will be stored in a locked file cabinet in the office of research staff and/or on password-protected computers behind a firewall. All study personnel who have contact with potential participants or data will have completed a course on human subjects’ protection that covers the importance of maintaining confidentiality. The PHI information collected for the purposes of this research study will be assigned a research study code and any personal identifiers will be removed from this information. Personal identifiers will not be attached to research data. All subject information will be handled in compliance with HIPAA.

# Potential Benefits to Subjects

Both the published literature and our preliminary data suggest that infants randomized to the placebo may enjoy improved health outcomes relative to infants that receive empiric antibiotics. These possible benefits include but are not limited to: 1) decreased incidence of NEC, LOS, and death, 2) improved postnatal growth, and 3) decreased incidence of common morbidities among preterm infants, e.g. retinopathy. We therefore believe it is plausible that the benefits of study participation will outweigh the risks of study participation.

# Data Management and Confidentiality

# The hypothesis of the primary outcome is that the incidence of composite adverse events (NEC, LOS, or death) is significantly different in ELBW infants receiving EA and infants receiving placebo. Based on 1,000,000 simulations for the group-sequential test for comparing two proportions and O’Brien-Fleming alpha spending method for two interim analyses, we need 382 infants in each arm to reach 90% power to test the hypothesis of the primary outcome using a two-sided significance level of 0.05. We base our event rates for the standard of care (i.e. infants receiving EA) on published literature regarding risk of adverse events with each day of antibiotics, on recent data from the Vermont Oxford Network ^51^, and upon data from the Pediatrix CDW. We used 13.5% and 22% for the placebo and EA groups, respectively in the sample size calculations. The effect size (odds ratio 1.35 *per day of* EA) is based on the scant literature available to assess the risk-benefit ratio of EA. Another assumption made in the calculation includes intraclass correlation (ICC) of 0.01. Based on in-house data for babies born at Magee-Womens Hospital in the past 4 years, we estimate that 20 out of 100 infants will be twins or triplets. Anticipating a 5% attrition rate, we will recruit 802 infants (from ~670 families; 401 infants in each arm) to reach at least 90% power.

Given recent admission numbers and anticipated moderate growth at NANO study sites, we estimate that the 7 sites will admit 2700 infants ≤28 weeks GA over a 4-year period, allowing 6 months for trial rollout and 3-6 months in Year 5 for final analyses and preparation of manuscripts. We estimate that 1755 infants (65% of total) will meet eligibility criteria.  Based upon our surveys of recently delivered mothers (see above) and our experience with NICU RCTs^173,174,176^, we believe it is reasonable to expect that parents of 30-50% of these 1755 infants will elect to participate. A 46% consent rate will yield approximately 807 study subjects (29.9% of total admissions ≤28 weeks GA).  If the rate of missing outcome data is 5%, then the trial will have 766 subjects – yielding 383 patients per group. This randomized clinical trial will thus have excellent power to detect clinically meaningful differences between the EA and placebo groups with respect LOS/NEC/Death. If the consent rate is lower than 46%, we will add enrollments at 1-2 additional sites.

We will compare the distribution of baseline variables between study arms to assess randomization success. To summarize continuous variables, we will use means and standard deviations or medians and interquartile range, and will use frequencies and percentages for categorical variables. Graphical methods (e.g., histogram, boxplot) will be used for assessing the overall shape of a continuous variable. No formal statistical hypothesis tests will be performed to avoid unnecessary testing.

The primary analysis is an intent-to-treat (ITT) analysis that includes two interim analyses at 1/3 and 2/3 enrollment, and a final analysis using O'Brien-Fleming stopping rules. The primary test for the primary outcome is that the **incidence of composite adverse events is significantly different in ELBW infants receiving EA compared to infants receiving placebo**.

The primary outcome will be analyzed using a generalized linear model (GLM) with a log link fitted via generalized estimating equations (GEE) with exchangeable working correlation matrix and employ robust variance estimates. This will account for non-independence of observations due to clustering of infants within families. The exchangeable correlation was selected primarily due to parsimony. Furthermore, infants within a twin or triplet can be considered exchangeable (i.e., the assigned infant ID within a set is arbitrary). The model will include treatment as fixed effects adjusted for site and gestational age. The primary hypothesis will be tested via the Wald test of the treatment assignment and effect estimates will be presented using risk ratios (RR) with 95% confidence intervals (CI).

In secondary analyses, we will examine each component (NEC, LOS, death) of the composite adverse outcome separately using the same analytic approach as the primary outcome.

*Microbiome analysis*. For microbiome analyses in **Aims 2 and 3**, assuming that the alpha diversity is normally distributed, we need a total samples size of 78 over two groups (39 per group) to detect a 43% reduction in alpha diversity (from 1.75 to 1) at the 14 day timepoint with a 5% level of significance and 90% power. This effect size is based upon our preliminary data and also published reports, as described in the Research Strategy. We will easily exceed power to discern differences in alpha diversity but will plan for much larger sample size to allow not only for taxonomic analyses across samples from EA and placebo groups, and also subgroups (as defined above for Aim 1 analysis).

Bacterial 16S rRNA gene sequences will be extracted from infant samples, amplified, and sequenced on the Illumina Miseq according to established protocols used routinely by the Morowitz, Gregory, and Peddada laboratories. In analyses of these samples, we shall investigate three important parameters, namely, the alpha diversity (Richness and Shannon Index), the beta diversity, and the differential abundance of individual bacterial taxa. We hypothesize that samples from EA subjects will contain increased abundance of pathogens (e.g. *Enterococcaceae* and *Enterobacteriaceae)* and decreased abundance of commensal anaerobes (e.g. *Bacteroidaceae* and *Bifidobacteriaceae*) relative to samples from infants receiving placebo.

**For Aim 2**, bacterial 16S rRNA gene sequences from weekly fecal samples collected during the first month of life from each experimental group will be compared at each time point and also with longitudinal trend analyses. Temporal trend analysis of alpha diversity will be performed with the Constrained Linear Mixed Effects (CLME) models package developed by the Peddada group. The analysis does not make any distributional assumptions, and takes into account the repeated measurement feature in the data since longitudinal measurements are obtained on each subject. The method is also robust against heteroscedasticity as it is based on Best Linear Unbiased Predictor (BLUP) residual bootstraps. The temporal trend analysis of beta diversity will be performed using an extension of PERMANOVA for repeated measurements data. As for temporal differential abundance analyses within each experimental group, we will use the newer version of ANCOM for detecting trends in abundance, while allowing repeated measurements as well as covariates. ANCOM was developed by Peddada and colleagues, and is based on the software ORIOGEN developed by Peddada. Note that differential abundance analyses of taxa between groups cannot be performed using standard ANOVA or t-test because these data reside inside a simplex. Currently, among the many methods used in the literature for differential abundance analyses, ANCOM is the only method that controls the false discovery rate at the desired nominal level.

We will measure the importance of clinical factors known to impact the infant gut microbiota, including mode of delivery, gestational age, diet, and maternal antibiotic exposure while remaining blinded to the identity of treatment assignment groups. In each case, we shall perform pairwise comparisons of individual variables (e.g. vaginal delivery vs. caesarean section) after adjusting for the remaining factors. Initially, we will consider diet (formula milk vs. human milk and maternal vs. donor milk) as a binary variable, but ultimately may perform more granular analyses regarding percentage of calories from each type of milk. Similarly, we will initially consider gestational age (23-26 weeks vs. 27-28 weeks) and maternal antibiotic exposure (yes/no) in pairwise comparisons, but later could consider gestational age as a continuous variable or could distinguish between classes of maternal antibiotics. We will also incorporate microbiome analyses of maternal vaginal swabs and early postpartum fecal samples, but anticipate such samples for only 25% of study subjects.

**For Aim 3 (exploratory)**, weight, length, and head circumference Z-scores will be calculated for birth and weekly postnatal growth measurements using Fenton and Olsen growth curves for preterm infants. Using the CLME methodology, we shall (a) develop nonparametric temporal growth curve model for each group of babies and (b) compare the temporal differences in growth curve patterns between the two groups of babies. The methodology will adjust for various confounders in the study. Furthermore, as noted earlier, CLME is entirely nonparametric as it does not make any distributional assumptions, robust to heteroscedasticity and does not rely on any parametric shape of the growth curve. Secondly, we shall also perform high dimensional regression analysis by regressing growth at time “t” on the vector of OTU counts from the previous time point using LASSO type high dimensional regression methodology used by us in Bertelsen et al.^191,197^. By including an interaction in the model between OTUs and experimental groups, we hope to detect taxa that are differentially associated with delayed and accelerated growth patterns in the two groups. The methodology will not only identify taxa that are associated with growth but will also provide statistical significance of the selected taxa.

The University of Pittsburgh will be the home of the NANO DCC (CRISMA BDMC) and the central IRB (Pitt HRPO). The DCC will be responsible for assuring the standardization, collection, management and quality control of the data as well as the statistical design and analysis of the study. The DCC will monitor the data from all sites.

16.1 CRISMA’s BDMC. CRISMA will serve as the study Data Coordinating Center (DCC). The core of the trial data management and communications system will be the project website, which will include a shared document section and a data system area. The website will include a personnel directory, project calendar, and shared documents. Sections with restricted access will be setup for members of the steering committee, and other individuals as needed. The data system area of the website will be the interface for data entry and data management. Reports comparing actual with expected recruitment will be developed for each site. Drop-out will also be monitored routinely. Protocol adherence reports will include enrollment of ineligible patients, follow-up data collection outside of protocol-defined windows, and important deviations from protocol. Reports will be provided to the sites, the Steering Committee, and the DSMB.

# They will monitor all aspects of study performance (e.g., enrollment, data processing time) and protocol compliance (e.g., randomization), as well as adherence to established adverse event reporting and event adjudication procedures. They will regularly provide reports to the clinical site coordinators addressing scheduling and delinquency. The project manager and PIs will conduct data monitoring site visits per a predetermined plan, prioritizing sites where specific data issues are identified or data concerns arise (risk-based monitoring).

16.2 Online Portal. The center of the NANO trial’s communications system will be a portal accessible via a password-protected study website which will be maintained by the DCC. The site will be used for day-to-day communications among the NANO study team by providing immediate communication, effective collaboration and project management. Access will be restricted to study investigators, research staff and committee members via unique usernames and passwords. The DCC’s password policy is consistent with National Institute of Standards and Technology (NIST) password policy. The primary interface is a collection of organized and individual web features, each representing a single tool which will vary in presentation and availability based on a defined user’s role (e.g., investigator, clinical site coordinator) and group association(s) (e.g., clinical site, committee membership).

16.3 Missing data. Despite best efforts to obtain follow-up data, we anticipate some loss-to-follow-up. We will describe the extent and reasons that data are missing, summarizing the proportion of patients with missing data for each outcome and by study arm and by site. We will compare baseline patient characteristics between those who have complete outcome data and those that do not. It is anticipated that, on rare occasion (<<5% of subjects), even before culture results are received, an attending neonatologist caring for a study subject will elect to prolong antibiotic therapy beyond 72 hours (e.g. due to clinical deterioration). This will be classified as a protocol deviation. Such infants will be analyzed according to intention to treat principles.

Optimal ITT includes analysis of data from all subjects randomized and cannot be directly adopted in the presence of missing data. To resolve this, we chose multiple imputations to conduct the ITT analyses. Multiple imputed datasets will be generated using multivariate imputation by chained equations, an approach designed for multivariate data that can accommodate mixed data types. We will use predictive mean matching and logistic regression to impute continuous and binary outcomes, respectively. We will generate 100 imputed datasets to maintain power, although 3-5 imputed datasets are usually sufficient to obtain excellent results^196^. We assume that missing outcome data are missing-at-random (MAR) in that they can be imputed reasonably well from the observed study data. We will perform imputation based on the study arm to which the patient was assigned. Auxiliary variables for the imputation model will include patient baseline variables (e.g., age, sex, race, and study site).

16. 4 Sensitivity analyses. We will assess the robustness of study findings to assumptions about missing data, the primary analysis population, and covariates used for adjustment. Specifically, we will perform the following:

- We will conduct a missing-not-at-random (MNAR) sensitivity analysis using control-based imputation in which all data are imputed based on the placebo arm.
- Since ITT generally biases towards no difference, we will perform a per-protocol analysis in which the complier average causal effect (CACE) will be estimated by using the treatment assignment as an instrumental variable.
- Baseline variables that were found to be strongly imbalanced between the treatment groups will be added as adjustment variables to the primary analysis model.

16.5 Data storage and security. Initial stages of data management will be done by experienced clinical data managers, database developers, data research associates, and data processing staff, supported by computer system analysts, programmers, and information technology specialists. These persons will be responsible for data quality and timeliness, documentation of processes and procedures, and training of data management staff.

Data will be entered directly into a secure, backed-up, 24-hour, web-based database using electronic forms developed by the BDMC staff with the assistance of project investigators and statisticians. Data entry screens will incorporate range and logical edit checks, both within and across forms. A data monitoring plan written before the start of data collection, will serve as a reference guide for the development of case report forms, data handling conventions, reporting, data dictionaries, supporting meta data, as well as project closeout activities, communication and coordination plans among the PIs, clinical teams, sites coordinators, and staff and faculty-level statisticians.

Identifiable medical record information will be used and will remain at each site until the study is completed in 2025. PHI information will not be shared across sites.

The link between your PHI and the study ID code will be destroyed ten years after study completion. Records of your baby will be maintained until he/she is 23 years of age. After that time, identifiers will be destroyed, and research data will be coded and retained anonymously indefinitely.

# Provisions to Monitor the Data to Ensure the Safety of Subjects

17.1 Safety monitoring and event reporting. As with any experimental procedure, there may be adverse events or side effects that are currently unknown and certain of these unknown risks could be permanent, severe or life threatening. We will capture the major potential adverse events related to EA during the study and examine each adverse event using standard reporting methodology. Research staff will strictly comply with IRB policies for the reporting of adverse events. To assist with reporting of adverse events, an event table will be distributed to all sites prior to enrollment.

*Definition of an adverse event (AE).* Any untoward medical occurrence in a subject temporally associated with the trial protocol, whether or not it is considered causally related to the trial protocol. Examples include: (1) significant or unexpected worsening or exacerbation of the condition under study, (2) or new conditions detected or diagnosed after protocol initiation even though it may have been present prior to the start of the study.

*Definition of a serious adverse event (SAE).* Any untoward medical occurrence during the index hospitalization that: (1) results in death, (2) is life-threatening, or (3) results in disability/incapacity.

All AEs and SAEs will be recorded from the time of randomization until hospital discharge. AEs and SAEs will be solicited from parents of subjects, attending physicians, and bedside nurses; the medical record will also be reviewed daily for the presence of events. Notification of coordinating center regarding any SAE will take place within 48 hours of recognition. The coordinating center will then be responsible for notifying the DSMB regarding all SAEs within 48 hours. When an AE/SAE occurs, it will be the site PI’s responsibility to review the pertinent records, notes, laboratory, and radiographic data. This information will be recorded along with the site PI’s impression of the diagnosis. The site PI will assess causality between the event and the study protocol using best clinical judgment, and this will be reviewed by the DSMB who will recommend if follow-up or modification of the study protocol is necessary. All deaths will be reviewed by the DSMB within 30 days of reporting said event.

17.2 Data safety and monitoring board (DSMB). The University of Pittsburgh Office of Clinical Research, Health Sciences / CTSI will provide the logistical management and support of the DSMB. A letter of support can be provided to sites, if needed. We propose to include neonatologists, maternal fetal medicine specialists, infectious disease specialists, and a statistician and/or epidemiologist experienced in the conduct of clinical research and unaffiliated with any study team members. Members will consist of persons independent of the investigators who have no financial, scientific, or other conflict of interest with the study. Written documentation attesting to absence of conflict of interest will be required. The DSMB will review the study protocol prior to study rollout and will meet on a semi-annual basis to review recruitment, retention, data completeness, protocol deviations, and adverse events. Safety data will be examined on an ad hoc basis if safety concerns arise from trial data or from external research or literature.

The DSMB will:

1. Review the research protocol, informed consent documents and plans for data and safety monitoring;
2. Evaluate the progress of the study, including periodic assessments of data quality and timeliness, participant recruitment, accrual and retention, participant risk versus benefit, adverse events, unanticipated problems, performance of the trial sites, and other factors that can affect study outcome;
3. Consider factors external to the study when relevant information becomes available, such as scientific or therapeutic developments that may have an impact on the safety of the participants or the ethics of the study;
4. Review clinical center performance, make recommendations and assist in the resolution of problems reported by the PI;
5. Protect the safety of the study participants;
6. Report on the safety and progress of the study;
7. Make recommendations to the PI, and if required, to the NIH / NICHD concerning continuation, termination or other modifications of the study based on the observed beneficial or adverse effects of the treatment under study;
8. Monitor the confidentiality of the study data and the results of monitoring;
9. Assist the PI by commenting on any problems with study conduct, enrollment, sample size and/or data collection.

The first meeting will take place before study initiation to discuss the protocol, approve the commencement of the study, and to establish guidelines to monitor the study. A safety officer (Chairperson) will be identified at the first meeting. This person will be the contact person for serious adverse event reporting. The DSMB will review the final protocol before commencing enrollment and then meet semi-annually and at any other needed interval (based on reports) to ensure that no serious adverse consequences occur because of either administering or withholding the intervention. An emergency meeting of the DSMB will be called at any time by the Chairperson should questions of patient safety arise.

DSMB reviews will consider the occurrence of adverse events, problems with loss of confidentiality, or other unanticipated problems, and will consider whether the anticipated benefit-to-risk ratio of study participation is altered by the findings in the safety monitoring process. Particular attention will be given to confirm that the study protocol is protecting the privacy of research subjects as anticipated. The following will be reported to the IRB: date of data and safety monitoring; summary of adverse event data including an assessment of intervention causality; summary of the assessment of relevant scientific literature and its impact on the design of the study; summary of procedural reviews conducted to ensure subject privacy. This report will be accompanied by a final conclusion regarding changes of the anticipated benefit-to-risk ratio and recommendations related to continuing, changing, or terminating the study. Recommendations to change the study will be accompanied with a detailed rationale for the proposed changes.

17.3 Interim analyses and stopping rules. The NANO Coordinating Center will conduct two interim analyses and a final analysis using O’Brien and Fleming stopping rules defined *a priori* controlling for an overall Type I error rate of 0.05. The first and second interim analyses will be scheduled at approximately 1/3 and 2/3 enrollment, respectively. The DSMB will review recruitment, safety, data collection, and analysis results at each interim analysis. Before trial completion, only the DSMB and a designated study statistician will have access to unblinded data. **During the interim assessments, the DSMB could recommend early trial termination due to unanticipated safety concerns, or due to one or more of the following reasons:**

1. A significant difference is found for the study hypothesis and a Z test statistic significance bound is crossed because of excess incidence of a composite adverse outcome (NEC, LOS, or death) in the EA arm.
2. A significant difference is found for the study hypothesis and a Z test statistic significance bound is crossed because of excess incidence of a composite adverse outcome (NEC, LOS, or death) in the placebo arm.
3. Failure to obtain success in the implementation of the trial either through failure to accrue subjects at the necessary rate, improper data handling, or inability to implement study protocols.

# Provisions to Protect the Privacy Interests of Subjects

# 18.1 Risk of loss of confidentiality. The risk of loss of confidentiality will be minimized by de-identifying patient samples such that no patient identifier information is accessible and preserving a key of de-identified samples which is password protected on a secure institutional server. Hardcopies of consent forms will be kept in locked offices. Patient identifiers will be preserved in this protected environment until the study is completed, and then they will be de-identified. A copy of the consent form will also be added to subject’s medical chart as well as a separate notation in EMR that they are a participating in the NANO trial. Non-identifiable data including microbiome data will be preserved until all analysis is complete. Data collection forms will be accessed via a secure web-based data entry system with access limited to study personnel only. The data will be transferred to the study database server via a secure internet connection. Database access is limited to the NANO data management team in accordance with state and federal regulations.

Parents of potential study subjects will be encouraged to take as much time as needed to ask questions and feel comfortable deciding whether to allow their children to participate or not.

# Compensation for Research-Related Injury

Emergency medical treatment for injuries solely and directly related to a study subject’s participation in this research study will be provided by the hospital participating in NANO caring for the particular study subject. Insurance providers may be billed for the costs of this emergency treatment, but none of those costs will be charged directly to study subjects or their families. If a subject’s research-related injury requires medical care beyond this emergency treatment, his/her family will be responsible for the costs of this follow-up care. At this time, there is no plan for any additional financial compensation. Subjects and their families do not give up any legal rights by agreeing to participate in the NANO Trial.

# Consent Process

# Sites will develop a protocol to identify women as early as possible that are admitted to participating hospitals and expected to deliver an infant at or before 28 weeks gestation. If eligible, and if the attending obstetrician provides permission, then trained research staff will explain the research protocol and the process of informed consent. Informed consent requires that the parents of the study subject understand the details of the study and agree, without coercion, to participation in the study. Study representatives at each site will introduce and explain the study to the parents and present them with the detailed consent form to read and review. It will be the responsibility of the site investigator to ensure that each parent is given full and adequate verbal and written information about the nature, purpose, benefit, and the potential risks of the study. A copy of signed informed consent document will be provided to the parents and another will be placed in the medical chart. The original document will be placed in the study files. Participation for study participation will be obtained from one parent. If there are multiples, a consent document must be obtained for each child.

Child assent for this trial involving newborn infants will not be possible.

Oral and written information provided to parents of possible study subjects will be available in Spanish and other languages, as needed.

# Process to Document Consent in Writing

# For each infant enrolled in NANO, documentation of informed consent and study enrollment will be entered into the official medical record.

#

# Setting

NANO study sites are university affiliated birthing hospitals that have participated in clinical trials involving preterm infants and/or their mothers. They have been selected for participation based upon clinical research experience, projected recruitment, infrastructure that will enable study protocol execution, and geographic diversity. We expect each site to recruit 2-3 subjects monthly, allowing for completion of study enrollment in approximately 4 years.

# Resources Available

Each NANO study site is equipped with clinical, laboratory, and health records systems that will enable prompt identification of subjects, randomization, and delivery of study drug or placebo. Additional sites may be added during the trial planning period. Primary study activities of subject enrollment and randomization will occur across all sites according to a single study protocol. Each site will have a lead investigator, and together they will ensure that the protocol and trial is implemented as designed. Given recent admission numbers and anticipated moderate growth at NANO study sites, we estimate that study enrollment can be completed within a 4-year period, allowing 6 months for trial rollout and 3-6 months in Year 5 for final analyses and preparation of manuscripts.

# Multi-Site Research

Collectively, NANO study sites will enroll **802 inborn infants** delivered in the obstetrical facilities of participating NANO study sites, and their mothers. The NIH Single IRB policy applies to this study, and we have identified no exceptions. As indicated in the attached letter, the Pitt HRPO will serve as the central IRB for the NANO trial, including the lead center, the data coordinating center, and those selected or added after award. Sites will sign a reliance agreement that will include a communication plan. All participating sites will agree to rely on the designated single IRB, without exception. All proposed study sites are SmartIRB centers, which will facilitate finalization of agreements between institutions. The project manager at The University of Pittsburgh, under supervision of Dr. Morowitz and Dr. Polin, will provide applicable conflict of interest management plans for relying site study teams to the Pitt HRPO, and will provide confirmation to the Pitt HRPO that relying site study teams have completed relevant training and are qualified to conduct the proposed research. The project manager will also provide documentation of IRB determinations to relying site study teams, and provide copies of IRB-approved materials to the lead study team. Additional responsibilities of the Pitt team will include providing the consent form template to relying site study teams, obtaining and collating study wide information for continuing review to the Reviewing IRB. Dr. Polin will coordinate interactions with the Data and Safety Monitoring Board, will lead all reviews of protocol deviations, and will report reportable events (e.g., unanticipated problems, noncompliance, subject complaints) to the Pitt HRPO in conjunction with Dr. Morowitz.

24.1 Communication. **Effective communication is the single most critical factor to achieve the goals of any clinical trial.** The project management strategy for NANO includes tools and techniques that place emphasis on communicating at the appropriate time with highly pertinent information and as widely as possible to all appropriate study personnel. A communication plan will be developed in the first phase of the study that identifies each stakeholder group, their communication needs, the facilitator, and the preferred frequency and methods of communication. The plan will also set the standard for how and when study information will be shared. Recognizing the importance of communication with all members of the NANO investigative team, the other investigators and clinic settings will have both regularly scheduled meetings with the PIs, and be encouraged to communicate via email and unscheduled calls as needed.

A major portion of the communication plan includes meetings and conference calls. For meetings and conference calls, standardized agendas and meeting minutes will be used to aid consistent communication. All minutes will include a list of agenda items, action items and follow-up of action items from previous meetings. The supporting documentation will be provided to committee members before each meeting with enough lead-time to allow for additional input.

The center of the NANO trial’s communications system will be a portal accessible via a password-protected study website which will be maintained by the DCC. The site will be used for day-to-day communications among the NANO study team by providing immediate communication, effective collaboration and project management. Access will be restricted to study investigators, research staff and committee members via unique usernames and passwords. The DCC’s password policy is consistent with National Institute of Standards and Technology (NIST) password policy. The primary interface is a collection of organized and individual web features, each representing a single tool which will vary in presentation and availability based on a defined user’s role (e.g., investigator, clinical site coordinator) and group association(s) (e.g., clinical site, committee membership).

24.2 Document Library*.* The Document Library will be used to store operations memos, data collection forms, manuals, training materials, and manuscripts so that study personnel have easy access to these materials. The Document Library area will have restricted access to individual libraries as needed.

24.3 Help Center. A Help Center/Frequently Asked Questions (FAQ) section of the website will be used to assist Co-investigators and Coordinators when answers are needed to questions, such as, clarification of inclusion/exclusion criteria or particular aspects of the study protocol. The web-based Help Center will list the most frequently accessed FAQ entries. A search dialog will permit the researcher to find other FAQ entries based on keywords. If the FAQ database does not sufficiently aid in finding an answer to a question, a direct link to the Help Request form is available. The Help Request form may be completed by the researcher and submitted online. Upon submission, an email notification will be sent to the appropriate DCC and/or CCC personnel. Once a request is resolved, the requestor will receive an email verifying the solution.

24.5 Data Management System. A link to the Data Management System will be accessible to study personnel who have appropriate authorization. This area will provide access to the data management system for data entry, verification, error correction, data tracking, reporting and randomization. In order to prevent unauthorized access to incoming data while in transit, this area of the website will use a server certificate to encrypt all incoming traffic over a secure channel using Secure Sockets Layer (SSL).
