# Supplementary material for: The NICU Antibiotics and Outcomes (NANO) trial: a randomized multicenter clinical trial assessing empiric antibiotics and clinical outcomes in newborn preterm infants
Source: Trials. 2022 May 23;23:428. doi: 10.1186/s13063-022-06352-3 (PMC9125935; doi:10.1186/s13063-022-06352-3)
Supplement: Supplementary file 4 — Additional file 4. [file 13063_2022_6352_MOESM4_ESM.docx]

## The NICU Antibiotics and Outcomes Trial

## Manual of Operating Procedures (MOOP)


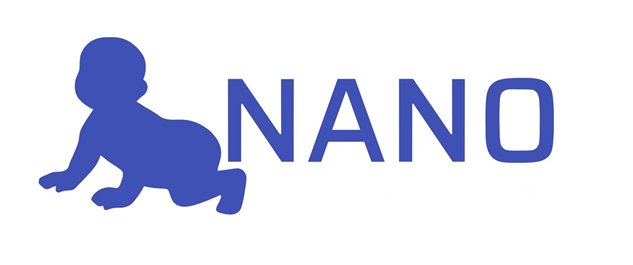


**IND Exempt (PIND 143353)**

**ClinicalTrials.gov ID (NCT03997266)**

**University of Pittsburgh IRB (PRO18010284)**

1. Introduction 4
2. Overview 4
3. MOOP Contents and Organization 4
   1. Study Aims 4
   2. Study Organization and Responsibilities 5
      1. NANO Study Roster 5
      2. Clinical Coordinating Center 5
      3. Clinical Sites 6
      4. Steering Committee and Publication Committee 6
      5. Pharmacy Activities 7
      6. NICHD Contact Information 8
   3. Training Plan 8
   4. Communications Plan 9
   5. Study Flowchart 9
   6. Recruitment Plan 10
   7. Screening and Eligibility Criteria 11
      1. Screening 11
      2. Screening Log 11
      3. Eligibility Criteria 11
      4. Co-Enrollments 12
      5. Medical Record Review 12
      6. Re-Screening 13
   8. Informed Consent and HIPAA 13
      1. HIPAA Authorization 14
   9. Participant Retention 15
   10. Study Intervention 15
       1. Assessing Eligibility 15
       2. Randomization 15
       3. Study Drug 17
   11. Blinding and Unblinding 20
   12. Study Measurements and Procedures 21
       1. Sample Collection 21
       2. Data and Specimen Banking 22
       3. Shipping of Samples 23
       4. Tracking Samples 23
       5. Data Collection 24
       6. Early Discontinuation 25
   13. Safety Reporting 25
       1. Reporting to the IRB 26
       2. Reporting to the DSMB 27
   14. Study Compliance 27
   15. Data Collection and Study Forms 28
       1. Source Documentation 28
       2. Participant Binder 28

**Table of Contents**

# 1.0 Introduction

3.16 Study Forms 28

- 1. Administrative Forms 28
  2. Data Safety and Management 29
  3. External Data 29
  4. Retention of Study Documentation 29
     1. Genomic Data Sharing 31
  5. Quality Control Procedures 31
     1. Standard Operating Procedures (SOPs) 31
     2. Site Monitoring 31
     3. Reports 31
  6. Confidentiality Procedures 32
  7. Data and Safety Monitoring Activities 32
  8. Study Completion and Closeout Procedures 32
     1. Participant Notification 32
  9. Publications and Access to Data 33
  10. MOOP Maintenance 34

**Appendices**

Appendix 1: NANO Clinical Site Protocol 35

Appendix 2: Historical Summary of NANO Protocol Changes 60

Appendix 3: Coordinating Center Study Roster 61

Appendix 4: Clinical Site Contact Information 63

Appendix 5: Steering and Publication Committee Members 69

Appendix 6: NANO Dispensing Example and Log 70

Appendix 7: Educational Materials 73

Appendix 8: Co-Enrollment and Application 77

Appendix 9: ICF Template 80

Appendix 10: NANO Event Table 91

Appendix 11: Protocol Deviation Log 93

Appendix 12: AE/SAE Event Table 94

This Manual of Operating Procedures (MOOP) should be used by The NICU Antibiotics and Outcomes Trial (NANO) Coordinating Center and all clinical sites. The role of this MOOP is to facilitate consistency in protocol implementation and data collection across participants and clinical sites. Use of the MOOP increases the likelihood that the results of the study will be scientifically credible and provides reassurance that both mother and infant safety and scientific integrity are closely monitored.

**2.0 Overview**

The NANO MOOP is a dynamic document that describes study organization, screening, recruitment, enrollment, randomization, data collection methods, data flow, electronic data collection forms (eDCFs) and quality control procedures. The MOOP will be updated throughout the study to reflect any protocol or consent modifications as well as refinement of the eDCFs and study procedures. The MOOP will be maintained in a format that allows it to be easily updated. Each page of the MOOP contains the version number and date. As pages are revised, an updated version number and associated date will replace the original pages in the MOOP. All previous versions will be archived.

# 3.0 MOOP Contents and Organization

The NANO MOOP details the study procedures and describes study-specific documents. A list of topics covered in the MOOP can be found in the Table of Contents on pg.2-3.

**3.1 Study Aims**

The goal of the NANO Trial is to test the hypothesis that the rate of adverse outcomes is higher in extremely low birthweight (ELBW) infants receiving empiric antibiotics (EA) compared to ELBW infants receiving placebo. There are three aims:

**Aim 1.** To test the hypothesis that the composite incidence of late onset sepsis (LOS), necrotizing enterocolitis (NEC), and/or death is significantly different in infants that receive EA and infants that receive placebo.

**Aim 2**. To test the hypothesis that fecal samples in the first month of life from infants receiving EA will contain lower diversity, higher abundance of pathogens and lower abundance of commensal anaerobes than fecal samples from infants receiving placebo.

**Aim 3.** (Exploratory) To identify microbial taxa associated with delayed or accelerated somatic growth (weekly weight and length z-scores) during the first month of life in infants receiving placebo or EA.

Appendix 1 contains Version 4 dated April 2020, of the NANO protocol. These appendices will be found throughout the NANO MOOP document. Appendix 2 contains a historical summary of all protocol changes.

**3.2 Study Organization and Responsibilities**

The main leadership bodies of the study consist of the Clinical Coordinating Core and the Data Coordinating Center. The organization also includes the clinical sites, the National Institute of Child Health and Human Development, and a Data and Safety Monitoring Board. The primary decision-making body of the study will be the NANO Steering Committee.

Non-urgent research related questions should be directed towards the MOOP. If an answer cannot be found, please reach out to the research coordinator at UPMC Children’s Hospital of Pittsburgh at the email address below.

The University of Pittsburgh will be the home of the NANO Data Coordinating Center (DCC), CRISMA Biostatistics and Data Management Core (BDMC), the central Institutional Review Board (IRB) and the University of Pittsburgh Human Research Protection Office (HRPO).

**Study procedure relation questions can be emailed to NANO@pitt.edu**

**3.2.1 NANO Study Roster**

The NANO study roster can be found in Appendix 3.

**3.2.2 Clinical Coordinating Center (CCC)**

The CCC will be responsible for the finalization of the clinical protocol, manual of operations, and the organization and oversight of the clinical sites. Under the leadership of the CCC are the sites which are responsible for recruitment and data collection. Dr. Morowitz, Dr. Katheria and Dr. Polin will share primary responsibility for the clinical aspects of the study.

The responsibilities of the NANO CCC include:

- Development and maintenance of the MOOP
- Development of the randomization scheme and procedures
- Serve as liaison to the central IRB
- Adverse event monitoring and reporting to the Data & Safety Monitoring Board (DSMB)
- Communication with clinical sites, scheduling of meetings and training sessions, responding to and documenting ad hoc communications
- Site visits (as needed) to ensure adherence to the protocol and procedures
- Quality control procedures
- Reports (e.g. enrollment, adverse events, participant status, site performance, quality control, DSMB, IRB)
- Distribution of all changes, updates and policies of reports and documents to all clinical sites, and to the DMSB as necessary

**3.2.3 Clinical Sites**

Clinical sites participating in NANO are university affiliated and/or private birthing hospitals that have previously participated in clinical trials involving preterm infants and/or their mothers. They have been selected based upon clinical research experience, projected recruitment, infrastructure that will enable study protocol execution and geographic diversity.

The roles and responsibilities of the investigators and their clinical sites will include:

- Compliance with the protocol, MOOP, local and central IRB, Federal and State regulations
- Identification and recruitment of potential study participants
- Assuring eligibility criteria
- Subject enrollment
- Protection of participant’s rights
- Data collection and entry into online web-based database
- Sample collection and shipment of samples to CCC
- Retention of subjects
- Communication of questions, concerns, and/or observations to the CCC

Appendix 4 contains a list of all active NANO sites and contact information.

**3.2.4 Steering Committee and Publication Committee**

The NANO Steering Committee, which will also serve as the Publication Committee, will ensure that results from the NANO Trial will be published in a manner consistent with Consolidated Standards of Reporting Trials (CONSORT) guidelines and the *Recommendations for the Conduct, Reporting, Editing and Publication of Scholarly Work in Medical Journals* established by the International Committee of Medical Journal Editors (ICMJE), which are intended “to ensure that contributors who have made substantive intellectual contributions to a paper are given credit as authors, but also that contributors credited as authors understand their role in taking responsibility and being accountable for what is published.”

Core, currently planned manuscripts will be drafted and submitted by the NANO Steering Committee. The plan is to submit core, currently planned manuscripts as authored by “The NANO Investigators,” with notation of both the writing committee and contributors. Specific journal policies may alter this approach.

The NANO Steering Committee will be responsible for making any final determinations by majority rule regarding prospective authorship, and will strictly adhere to the following four criteria for authorship set forth in the ICMJE’s *Recommendations for the Conduct, Reporting, Editing and Publication of Scholarly Work in Medical Journals*:

- Substantial contributions to the conception or design of the work; or the acquisition, analysis, or interpretation of data for the work; AND
- Drafting the work or revising it critically for important intellectual content; AND
- Final approval of the version to be published; AND
- Agreement to be accountable for all aspects of the work in ensuring that questions related to the accuracy or integrity of any part of the work are appropriately investigated and resolved.

These recommendations further advise that all authors formally disclose any conflicts of interest pertaining to the conduct or reporting of the research in question. Specifically pertaining to large multi-author or collaborative groups, the recommendations state: “When submitting a manuscript authored by a group, the corresponding author should specify the group name if one exists, and clearly identify the group members who can take credit and responsibility for the work as authors.”

Secondary and ancillary studies will be evaluated and approved by the Steering Committee on a rolling basis. The NANO Steering Committee will review and approve all manuscript proposals and final manuscripts prior to submission. Authorship on secondary and ancillary studies will be based on written input and will follow definitions listed below. The NANO contributor list will be appended and submitted with each of these manuscripts.

Contributors who meet fewer than all 4 of the criteria below for authorship will most likely not be listed as authors but should be acknowledged if data collection is complete at their respective clinical site. Such contributors may include individuals who:

- Served as clinical investigators or clinical site study coordinators
- Assisted with study design, data collection, data analysis, or manuscript preparation
- Provided purely technical help and/or writing assistance
- Served as scientific advisors

Because readers may infer that acknowledged contributors endorse the data and conclusions presented in a manuscript, contributors must provide written permission to the CCC to be acknowledged within NANO related publications.

Appendix 5 contains the list of NANO Steering Committee members.

**3.2.5 Pharmacy Activities**

Logistical challenges are inherent in conducting a randomized, placebo controlled, double blinded multicenter trial with a time-sensitive protocol and have been taken into consideration. The NANO CCC has had many discussions with the investigative pharmacists at the NANO clinical sites indicating prior experience with round-the-clock interventions. We have budgeted for and planned the details of pharmacy activities including estimated workload, space, and investigative pharmacist time; staff training prior to study launch; purchase of drugs; and monitoring of supplies. The specifics of integrating prescription and delivery of study drug into the patient record will be individualized at each institution with either custom digital or hardcopy builds.

Jason Sauberan, PharmD will serve as the lead pharmacist for the NANO Trial.

**3.2.6 NICHD Contact Information**

The Eunice Kennedy Shriver National Institute of Child Health and Human Development (NICHD) is at https://www.nichd.nih.gov/. The Grants Management Specialist is responsible for the negotiation, award and administration of this project and for interpretation of Grants Administration policies and provisions. The Program Official is responsible for the scientific, programmatic and technical aspects of this project. These individuals work together in overall project administration. Prior approval requests (signed by an Authorized Organizational Representative) should be submitted in writing to the Grants Management Specialist. Requests may be made via email.

Grants Management Specialist: Saiyda Khan

Email: khansa@mail.nih.gov Phone: (301) 496-5001

Program Official: Marion Koso-Thomas, MD, MPH

Email: marion.koso-thomas@nih.gov Phone: (301) 435 6873 Fax: (301) 496-3790

National Institute of Child Health and Development (NICHD) will be considered the sponsor of NANO. The program official (Dr. Marion Koso-Thomas) will serve as the NICHD liaison to the NANO DSMB.

**3.3 Training Plan**

Site education will be provided to each site individually 3-4 weeks before their projected roll out date. Once site enrollment has begun, additional training will be provided as needed. Education will consist of:

1. Site Initiation Calls
   - Initiation calls will be conducted by the project manager and principal investigator (PI) prior to the start of enrollment at each clinical site. Calls will be focused on ensuring each site understands all components of the trial, (i.e. screening plan, sample collection, SOPs, etc.) has study-related, IRB approved documents organized and gives the opportunity for the PI to address any remaining logistical issues and/or questions.
2. Video Based Trainings
3. Educational Presentations
   - PowerPoint presentations will be distributed from the CCC to each site’s coordinator and PI. Sites will decide how to use these presentations to train staff who will be assisting with the trial.
4. Site Visits
   - Site visits prior to enrollment of their first patient will be done on an as needed basis. The Steering Committee and the clinical site PIs will determine if a site visit is necessary.
5. Check-In Calls
   - Sites will be asked to participate in monthly “all site” calls. This call will allow each site the opportunity to share best practices with each other while also getting important updates from the CCC.

**3.4 Communications Plan**

Effective communication is the single most critical factor to achieve the goals of any clinical trial. The project management strategy for NANO includes tools and techniques that place emphasis on communicating highly pertinent information in a timely fashion to all appropriate study personnel. A major portion of the communication plan includes scheduled meetings and conference calls. Standardized agendas and meeting minutes will be used to aid consistent communication. Minutes will include a list of agenda items, action items and follow-up of action items from previous meetings. Supporting documentation will be provided to committee members before each meeting with enough lead-time to allow for additional input. The CCC coordinator will be responsible for developing and sharing these documents.

The center of the NANO Trial’s communications system will be an online web-based database accessible via a password-protected study website which will be maintained by the DCC. Key aspects of this database include patient randomization, electronic data collection forms (eDCF), adverse event reporting, tracking logs for sample collection and shipment and links to study-related documents. The database can also be used for day-to-day communications amongst the NANO clinical teams by providing immediate communication, effective collaboration and project management. If a clinical site has an urgent question, they should call or email the CCC directly before referring to the database (NANO@pitt.edu). Access will be restricted to study investigators, pharmacy, research staff and committee members via unique usernames and passwords. The DCC’s password policy is consistent with National Institute of Standards and Technology (NIST) password policy. The primary interface is a collection of organized and individual web features, each representing a single tool which will vary in presentation and availability based on a defined user’s role (e.g., investigator, clinical site coordinator) and group association(s) (e.g., clinical site, committee membership).

**3.5 Study Flowchart-** Shown in Figure 1.

**Figure 1.** Study Flow Diagram


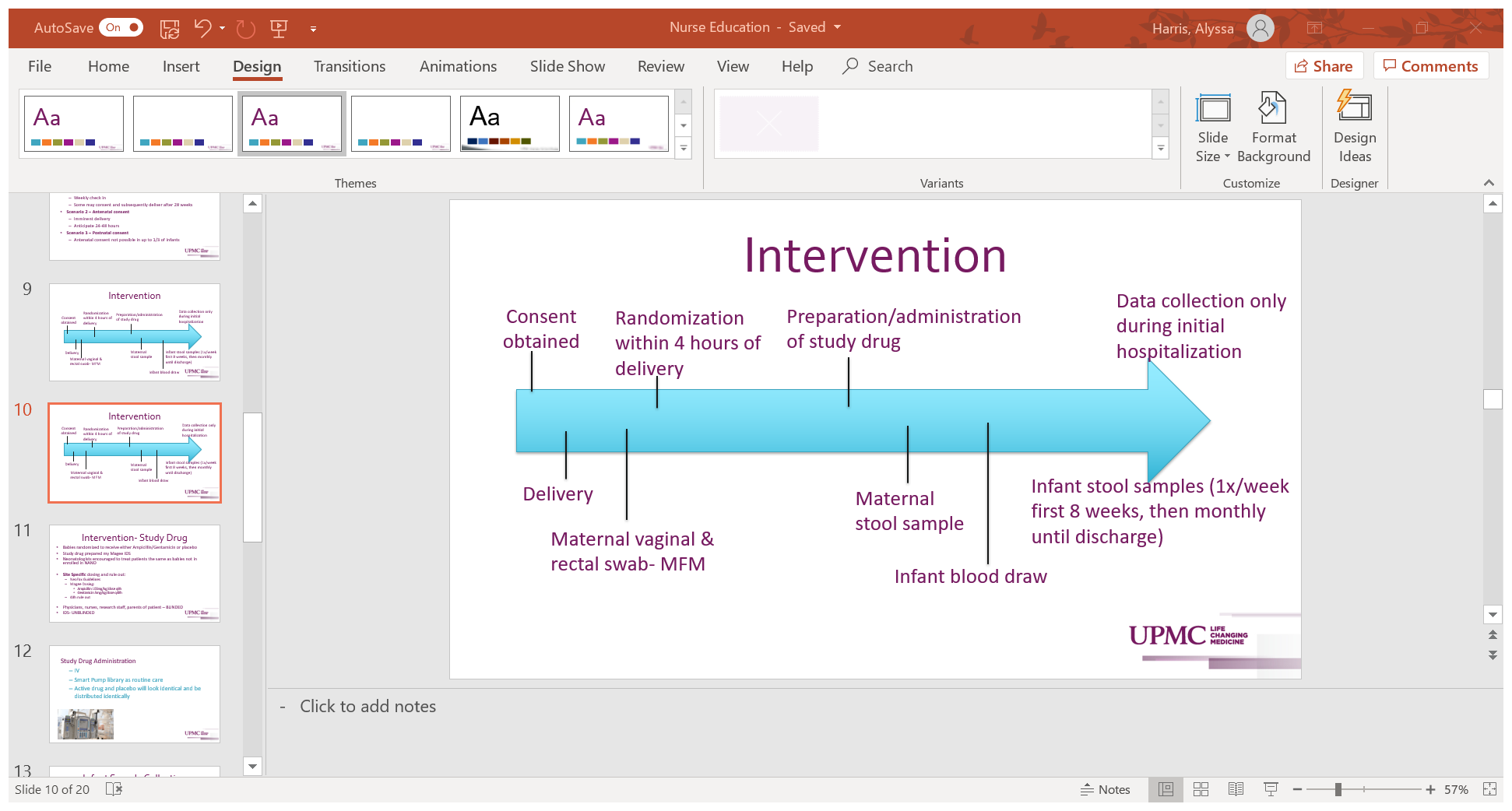


**3.6 Recruitment Plan**

To assist clinical sites in recruiting study participants, this section of the MOOP describes the target population and suggests recruitment strategies. Sites will develop site-specific protocols to identify women as early as possible that are admitted to participating hospitals and expected to deliver an infant at or before 28 weeks gestation, which will also be notated as 28 6/7 weeks throughout this manual. Women will be identified by the neonatal team, maternal fetal medicine (MFM) or by medical record screening which may be performed by study coordinators and additional research staff.

Either the treating Obstetrician/MFM specialist, mother’s bedside nurse, or consulting neonatologist will ask permission for the study team to approach the mother to discuss “a research study related to the administration of antibiotics in preterm babies.” If the mother agrees, the caregiver will inform a member of the study team, preferably the coordinator, who will approach the mother to discuss the study and obtain consent. This introduction of the NANO Trial by the mother’s caregiver is essential to prevent “cold-calling.”

Study discussion will begin with a brief video summarizing the study. This video will be accessible at all sites in both English and Latin American Spanish. The video should be shown through a secure URL. The video may be shown to family members in addition to the patient and can be viewed as many times as the patient and their family wishes. Showing this video is not required but is strongly recommended. If a site does not plan to show this video, it should be documented.

**Note**: Showing this video does not replace the informed consent process. Physicians and/or IRB approved staff must still verbally review each page of the consent document. The video is meant to be a supplemental recruitment tool.

An educational pamphlet will also be provided to sites. Sites will fill in site specific contact information and distribute the pamphlet to parents of potential subjects. Pamphlets include general study information including rationale, brief summary of antibiotics, study procedures and site contact information. If the video is not shown at a clinical site, the pamphlet should still be given to the mother. Each site is responsible for printing their own pamphlets. If any guidance is needed, please reach out to the CCC. A template of this pamphlet can be found in Appendix 7.

If a mother is interested in the study after watching the video and reading through the pamphlet, please refer to Section 3.8 on obtaining informed consent.

Each clinical site is expected to recruit 2-4 subjects monthly, allowing for completion of study enrollment in approximately 4 years.

The Steering Committee will continuously monitor recruitment and will work with clinical sites to meet recruitment goals. If enrollment is below target goals, we will review study-wide data regarding the number of potential patients screened and the number randomized, and the reasons for ineligibility or refusal to participate. Solutions to recruitment problems will be site-specific, based on site and study-wide recruitment data.

**3.7 Screening and Eligibility Criteria**

The NANO Trial is unique in that there is a two-part screening process. The two-part screening process involves determining maternal eligibility and then infant eligibility. Accurate maternal and infant screening are crucial to maintaining the integrity of the NANO Trial.

**3.7.1 Screening**

Labor and delivery antenatal units will be screened for admissions of all mothers admitted with pregnancies ≤28 6/7 weeks gestational age (GA). All potential mothers should be screened to increase the likelihood of an eligible patient. All screenings should be logged to track possible selection bias. Both the mother and infant must be eligible to participate in the NANO Trial. Screening should be performed by site coordinators and research staff. It is expected that some eligible mothers will provide consent but deliver an ineligible baby, thus precluding their participation in NANO.

**3.7.2 Screening Log**

Screening logs provide documentation of patients that are screened for study eligibility. NANO clinical sites will be instructed to maintain patient screening logs per standard practice. The CCC will provide a template for sites to use. At any time, the CCC can request copies of deidentified screening logs to track enrollment.

**3.7.3 Eligibility Criteria**

Study eligibility is determined by specific inclusion and exclusion criteria that are outlined in the study protocol.

NANO enrollment will be restricted to populations of extremely low birth weight (ELBW) infants that are NOT considered low risk for early onset sepsis (EOS) and are NOT considered high-risk for EOS. The rationale for this strategy is to target infants for whom to prescribe or not prescribe EA remains most challenging based upon existing published research.

1. **Participant Inclusion Criteria**
   1. Newborn infants born with gestational age of 23-28 6/7 weeks
   2. Inborn infants at participating study sites
2. **Participant Exclusion Criteria**
   1. Infants at low risk for EOS – born for maternal indications via caesarean section with ROM within 6 hours of delivery, no attempts to induce labor, and no concern for maternal infection
   2. Infants at high risk of EOS born to mothers with intrapartum fever (> 38ºC) or clinical diagnosis of chorioamnionitis (suspected or definite)
   3. Infants with respiratory insufficiency requiring invasive mechanical ventilation and FiO_2_ > 0.40 or non-invasive ventilation and FiO_2_ > 0.60 at time of randomization
   4. Infants with ongoing hemodynamic instability requiring vasopressors or more than one fluid bolus at time of randomization
   5. Clinician concern for sepsis due to physical exam findings, e.g. lethargy
   6. Major congenital anomalies
   7. Infants not anticipated to survive beyond 72 hours
   8. Infants who have received antibiotics prior to randomization
   9. Mothers that are <18 years old at time of enrollment

**3.7.4 Co-Enrollments**

Subjects enrolled in NANO must receive approval from the Steering Committee before enrolling in other studies. The Steering Committee will evaluate competing outcomes in other studies to determine eligibility. In order to receive co-enrollment approval, sites will download co-enrollment documents from the online database, fill out and submit to the Steering Committee. A copy of this document can be found in Appendix 8.

**3.7.5 Medical Record Review**

The NANO Trial has been approved to review medical records once receiving written consent and HIPAA authorization. Maternal medical record information will be reviewed to verify eligibility.

Medical record information to verify eligibility for neonate subjects will be collected after written parental permission and HIPAA authorization has been obtained. Prior to randomization of neonate subjects, medical record information will be assessed by study coordinators and confirmed with the treating physician to ensure neonate eligibility.

**3.7.6 Re-Screening**

Due to the design of the NANO Trial, maternal screening will be performed twice. Maternal screening will occur after providing written consent and again after the neonate is born. The original written consent is applicable for this second screening and another consent form is not required. Screening must be performed prior to randomization.

Maternal eligibility will be monitored from the time informed consent is obtained until delivery. Maternal monitoring should occur several times a week by research staff. Monitoring will consist of checking the patient’s medical chart to ensure that she is still eligible before giving birth. Maternal monitoring is crucial to allow for efficient randomization of eligible mother/baby dyads, appropriate use of sample collection supplies and research staff’s time and energy.

Any mother/baby dyad that met inclusion criteria prior to delivery will be re-screened after delivery to verify that all inclusion criteria are still met and that no exclusion criteria are present. Screening for exclusion after delivery will include, but not limited to:

- Overall clinical status of newborn
- Evaluation of any required respiratory and oxygen support
- Hemodynamic instability
- Any concern for neonatal sepsis
- New diagnosis of maternal chorioamnionitis
- Administration of antibiotics

Assessment of infant inclusion/exclusion criteria will be performed by IRB approved staff. Inclusion/exclusion criteria will be logged into the online web-based database prior to randomization.

**3.8 Informed Consent and HIPAA**

This section of the MOOP describes the specific instructions for obtaining informed consent. Each NANO site will follow the University of Pittsburgh’s IRB with exceptions for local context as dictated by a sites local IRB. Each site is required to use the University of Pittsburgh IRB approved consent template.

If a patient agrees to participate in the NANO Trial after watching the informational video, reading through the pamphlet and discussing the trial with research staff, written or electronic authorization and consent will be obtained by licensed physician study investigators who are knowledgeable of the study and who have knowledge and training in the consent process and in the protection of human subjects. If a mother has multiples, a signed consent document is required for each infant. Consent may be obtained in person or through video conferencing. Video conferencing will be done in real time when obtaining consent. Physicians will be in a private room and will not save or record any of the consenting process. Once consent is obtained, the video will be immediately closed. Obtaining consent in person is preferred, however it is understood that a physician may not always be available in person and must use video conferencing.

Due to the nature of the NANO Trial, mothers can be approached before delivery or immediately after delivery to provide informed consent for themselves and their baby to participate in the research study. Because variable amounts of time will have elapsed between mothers who have provided prenatal consent and delivery, research staff will meet with these mothers while they are on labor and delivery floors. These visits are brief check-ins with the enrolled patient to ensure that there are no questions or changes in relevant health information that may affect eligibility. If antenatal consent is not possible, research staff will attempt to seek postnatal consent as clinical circumstances permit.

On the consent document there is a line for the mother’s name, mother’s signature and infant’s name. If prenatal consent is obtained and the mother’s baby is randomized to the trial, research staff must go back to the original consent document and fill in the baby’s legal name following birth. Anytime research staff makes changes to the consent document, the individual must place their initials and date of revision on the document next to the area that was changed and/or information was added. Once maternal consent is obtained, a signed copy may be given to the patient for their personal files. Patients should be reminded that this document is important to retain for information on who to contact with questions.

Appendix 9 contains a copy of the NANO informed consent template.

**3.8.1 HIPAA Authorization**

The Health Insurance Portability and Accountability Act (HIPAA) authorization form must be reviewed and signed by the study participant in addition to reviewing and signing the consent form. This determination is made, and the format of the HIPAA authorization is established by the local IRB. The NANO informed consent form in Appendix 9 includes HIPAA language.

Site investigators should review information provided in Impact of the HIPAA Privacy Rule on NIH Processes Involving the Review, Funding, and Progress Monitoring of Grants, Cooperative Agreements, and Research Contracts http://grants2.nih.gov/grants/guide/notice-files/NOT-OD- 03-025.html and contact their appropriate institutional officials to learn how the Privacy Rule applies to them, their organization, and their specific research project. Another helpful resource is Protecting Personal Health Information in Research: Understanding the HIPAA Privacy Rule, NIH Publication 03-5388 at: http://privacyruleandresearch.nih.gov.

The encrypted data will be stored at the NANO CCC within the project database, which resides on the secure, password protected network drive behind an enterprise firewall. Permission to access this database is restricted to the honest broker. The honest broker will provide necessary PHI and/or related contact information to the NANO Coordinating Center Long Term Follow-Up Core for required follow up calls and NDI searches, blinded to study intervention. All PHI and related contact information will be destroyed once long-term follow up survival data is obtained. No investigator or representative/agent of the study sponsor will have access to this protected information.

**3.9 Participant Retention**

Participant retention requires careful planning and continuous efforts which helps to ensure a successful study. Our strategy to maximize both enrollment and retention of study subjects relies upon continual communication between mothers and providers taking care of the infant. Site-specific and centralized strategies will be adopted to optimize retention.

Plans for participant retention include, but are not limited to:

- At the time of informed consent and during maternal check-ins, research staff will strive to explain the details and timeline of the study protocol as clearly as possible.
- Mothers of infants enrolled in the trial will be provided contact information for local research coordinators and/or site PIs to ask questions.
- Research staff will attempt to be as flexible as possible regarding the timing of sample collections, respecting the privacy of NICU families and respecting the limited time and availability of NICU nursing staff.
- As often as possible, research staff will strive to convey to NICU providers and families that the motivation for this trial is to minimize antibiotic exposure and to improve outcomes for preterm infants.

**3.10 Study Intervention**

**3.10.1 Assessing Eligibility**

Eligibility will be assessed by an IRB approved PI or Co-Investigator (Co-I) supporting the NANO Trial. Research staff will meet with PI’s or Co-I’s to assess infant eligibility based on the inclusion/exclusion criteria. Maternal eligibility should have been monitored throughout her hospital stay and re-assessed immediately before this process. For mothers who provided postnatal consent, maternal eligibility should be confirmed by medical chart review before assessing infant eligibility. Each clinical site will outline their maternal and infant assessment procedures during their site initiation call with the CCC. If a physician has questions about whether a baby is eligible for the NANO Trial, the PI should be contacted immediately for clarification.

**Note-** Coordinators and/or research staff must meet with the PI or Co-I’s listed on the clinical site’s IRB to assess eligibility. **Eligibility assessment is not valid from anyone who does not have training documentation or is not listed on the IRB.**

Pocket cards have been created for use by the PI’s and Co-I’s as a quick reference for eligibility assessment. The NANO pocket card template can be found in Appendix 7. Use of this pocket card is optional and may be modified as each clinical site feels fit.

**3.10.2 Randomization**

After eligibility is determined, site coordinators and/or other research staff, will input maternal and infant patient data into the web-based database. Once data is entered into the database, eligible families will be randomized 1:1 using web-based block randomization stratified by study site to receive ampicillin and gentamicin (empiric antibiotics (EA)) or a volume matched equivalent of normal saline (placebo). Multiples (i.e. siblings) will be randomized to the same treatment arm. All staff besides pharmacy will be blinded to patient’s assigned arm. Treatment arm assignment and pertinent infant information will be sent to each site’s investigational pharmacy via email where study drug will be prepared and sent to the patient’s location. The staff member who performed randomization will see a confirmation message that randomization was successful and that pharmacy was notified. Email and phone information (as a back-up) will be collected from each site’s pharmacy prior to roll out to set up this randomization process.

**Randomization must occur within the first 4 hours of life**.

To facilitate proper implementation of the study intervention, the coordinator should immediately inform the attending physician, nurse practitioner and/or providers that consent has been given and randomization has occurred. A randomization note should also be included in the patient’s EMR.

Each enrolled participant will be assigned a study number. The structure will be a numerical portmanteau of the Site ID code number (see list below) followed by a 4-5 digit participant code number. For example, the first participant at Yale will be 1320001. The study team will also be using prefixes “MAT” for maternal and “INF” for infant data collection purposes, thus “INF1320001” may be the full ID furnished to the pharmacy. Since there are no study drugs for mothers, using “INF” in pharmacy documentation is unnecessary.

**SiteID Site**

43 Brigham & Women's Hospital

123 Morgan Stanley Children's Hospital

124 Sharp Mary Birch Hospital for Women & Newborns

125 Thomas Jefferson University

126 Univ. of Virginia Children's Hospital

127 Penn Medicine, Pennsylvania Hospital (PAH)

128 Penn Medicine, Hospital of the University of Pennsylvania (HUP)

129 Univ. of Kansas Hospital (KUMC)

Children’s Mercy (CMH) (will add Site ID when site goes live)

131 University of Pittsburgh Medical Center - Magee-Women's Hospital

132 Yale New Haven Children's Hospital

133 Norton Children's Hospital

134 University Hospital San Antonio, TX

Randomization assignments will be documented via the web-based database so that they can be reviewed during a data review or audit. All NANO sites will maintain the assigned and blinded randomization code in an automated, computerized log that is separate from the study data. This separate sheet will be on the same online database used for NANO, but in a different location and will not be accessible to majority of research staff.

Technical Issues: If the online-web based database is having technical issues and staff cannot perform randomization, the site coordinator should immediately call Michael Morowitz, the PI, at (312-342-5590) who will perform a manual randomization. Random assignment will be determined by a coin-flip method. The PI will immediately communicate treatment assignment with 2 groups: 1) Clinical site’s investigational pharmacy and 2) University of Pittsburgh’s DCC (crismadmtapplication@upmc.edu). The DCC will assign manually randomized patient’s a study number and update the block randomization scheme. The DCC will escalate the issue to UPMC or Pitt who will work to resolve the technical issue.

All patient pre-randomization data collected should be entered into the web-based databases eDCF by site coordinators upon receiving patient study number.

**3.10.3 Study Drug**

Intravenous gentamicin, ampicillin, and normal saline are the “study drugs” that will be used. One study arm is the combination of ampicillin + gentamicin, the other arm is placebo + placebo. In other words, antibiotics vs no antibiotics. There are no other permutations (e.g. ampicillin + placebo vs. placebo + gentamicin). Ampicillin and gentamicin are referred to as “Empiric Antibiotics” or “EA” in the Study Protocol.

In order to maintain blinding, all study drugs (EA or placebo) must be prepared by unblinded pharmacy personnel and not by NICU nurses. Physical location of preparation (e.g. NICU vs. pharmacy) may follow site-specific standards as long as blinding is maintained. Study drug supplies will come from site standard formulary supplies. Sites are not being supplied with or reimbursed for study drug. Research subjects or their insurance providers will not be charged for any of the procedures performed for the purposes of this research study.

Dose and concentration: Each site may dose and prepare ampicillin and gentamicin based on local guidelines. The dosage and concentration are not dictated by the Study Protocol. The corresponding “dose” of placebo ampicillin and placebo gentamicin will be a matching volume of normal saline. Whatever the site-specific volume is for a participant’s specific dose of ampicillin and gentamicin, the placebo volumes should be the same. For example, if a site normally doses ampicillin 50 mg/kg at a concentration of 100 mg/mL, and gentamicin at 4 mg/kg at a concentration of 10 mg/mL, a participant who weighs 1 kg and who is randomized to placebo would receive 0.5 mL of saline instead of ampicillin and 0.4 mL of saline instead of gentamicin.

- Sodium exposure from study related use of saline; the amount of sodium in 0.5 mL of 100 mg/mL ampicillin sodium reconstituted with sterile water is 0.145 mEq. The amount of sodium in 0.5 mL of normal saline is 0.077 mEq. These differences are clinically insignificant. Commercial pediatric gentamicin contains no or negligible sodium while 0.4 mL of saline is 0.06 mEq which is also clinically insignificant for a 1 kg baby.

Dispensing: Per protocol, the first dose must be administered within 60 minutes of randomization. The quantity of ampicillin or placebo and gentamicin or placebo doses after the first doses will be determined by site local standard dosing regimens. For example, if ampicillin is dosed every 12 hours and gentamicin every 48h, participants will receive 3 more ampicillin or placebo doses after their first dose, and no further gentamicin or placebo doses. The number of doses dispensed at any one time, including at the time of the first doses, may follow site-specific NICU drug dispensing standards. Dosing must be specified during on-boarding.

Labeling: All investigational drug products (EA or placebo) should be labelled in such a way to maintain blinding. The dose, volume, and instructions that appear on the label and in the electronic medical record (EMR) orders and eMAR should be the same between drug and placebo. Label content may follow local site standards for investigational drugs and BCMA. In the syringe label examples below, blinded personnel handling the medications would not know what study arm the participant is assigned to.

Subject Name Unit/Bedspace

Hospital ID#

Gentamicin/Placebo Inj Investigational

4 mg/0.4 mL

Subject Name Unit/Bedspace

Hospital ID#

Ampicillin/Placebo Inj Investigational

50 mg/0.5 mL

Documentation: Appendix 6 contains a Dispensing Log which should be used by unblinded pharmacists and technicians.

Ordering: Study personnel or clinical care providers are expected to place orders for study drugs. Ideally, given the time-sensitive need to start study drug within 60 min of randomization, ordering will ideally involve CPOE workflows analogous to standard care ordering of empiric antibiotics for EOS. Sites are free to develop ordering workflows (e.g. who does the ordering and how they do it - CPOE vs paper) that suit their local safety standards and customs for investigational drugs; whatever works best for the site that can accomplish the needed timeliness while maintaining blinding. Study drug orders should preferably automatically discontinue after completion of the study period (e.g. 36 hours duration or as determined by local standards).

Study Drug Rescue Antibiotics:

1. Conversion to blinded EA
2. Addition of open-label antibiotic therapy within the first 48 postnatal hours
3. Early study drug discontinuation

**Conversion to blinded EA:** it is anticipated that a small % of study participants will experience worsening of their clinical condition prompting their NICU providers to want a guarantee the participant actually receives ampicillin and gentamicin and not only placebo. In order to guarantee this without unblinding the original treatment assignment, a second set of study drug orders will be placed. For this second set, if the original assignment was placebo, pharmacy will prepare ampicillin and gentamicin. If the original assignment was ampicillin and gentamicin, pharmacy will prepare ampicillin and placebo. If the second set of study drug orders will be dispensed differently than what is in the MOOP, this will be documented in a clinical site’s pharmacy manual.

The order and label should include some indication of set number to avoid preparation/dispensing/administration errors. If this rescue antibiotic scenario occurs, providers will have guaranteed that the infant is receiving ampicillin and gentamicin. Providers will not know what the initial order set consisted of, in order to maintain the integrity of the blind. The second set of orders should discontinue at the same time as the first round of orders.

**Open label non EA antibacterials:** may be added to study drugs at the discretion of the Attending Neonatologist to empirically treat suspected/confirmed bacterial threats not optimally covered by study drugs (e.g. penicillin for syphilis, ceftriaxone for gonorrhea, azithromycin for chlamydia, vancomycin or nafcillin for Staph, meropenem or cefepime for MDR GNR). These are ordered outside of the study and are not the responsibility of the IDS. Routine Candida prophylaxis (e.g. fluconazole) is not considered an open-label antibacterial for the purposes of this study and its use is not viewed as rescue therapy.

**Early Discontinuation**: study drugs may be discontinued at the discretion of the Attending Neonatologist prior to the completion of the study period. The Attending should ideally discuss their desire to discontinue study drugs early with the PI and research team before doing so. Potential reasons for discontinuing include:

- The Attending wants the participant to receive **open label** ampicillin + gentamicin. NICU prescribers will discontinue study drugs and begin open label treatment outside of the study (IDS not responsible for dispensing open label orders).
- They have decided that EA treatment is no longer necessary for clinical care.

Giving ampicillin and gentamicin after the study period is at the discretion of the Attending Neonatologist. In such cases, NICU prescribers will place new orders for these antibiotics per routine care. IDS is not responsible for these antibiotics since they are beyond the study period and the participant has completed the study drug therapy. The NICU treatment team will decide their dosing and start times. A sensible approach would be to stay on the same schedule as the study drugs. If the participant was previously converted to blinded EA, the start time can be the split difference between the two sets’ schedules. Gentamicin plasma concentration monitoring may be used to help with determining start times (see next section). Prescribers will not be unblinded to determine these start times. They should remain blinded and should not count study doses towards the total days of therapy that the baby ultimately receives.

Gentamicin plasma concentration monitoring: It is expected that a small percentage of participants will receive gentamicin after the study period. This will trigger site-specific procedures for routine care gentamicin therapeutic drug monitoring, the results of which may make it possible for blinded providers and staff to discern group allocation. This is expected and unavoidable. Pharmacists who routinely perform clinical pharmacokinetics in the NICU should therefore be part of the unblinded study pharmacist team. If allowed by site practice standards, clinical PK assessments that include dosing histories may be kept with the participant’s Dispensing Log rather than in their medical chart, in order to minimize the risk of unblinding.

Administration: Nurses should use site standard smart pump libraries for ampicillin and gentamicin as they would for standard care to administer study drug doses regardless of study arm assignment. Nurses are blinded so there should be no difference in administration technique between study arms. Recording administration of study drug on the participant’s eMAR is recommended to facilitate data collection by the research team.

Co-administration of study drug with other agents: Sites should follow their local guidelines for EA compatibility with other intravenous therapies that participants may be given during routine care. Sites should treat all study drugs as active drug when making co-administration recommendations. For example, if a participant randomized to placebo happens to be receiving a midazolam infusion and a site’s standard practice is to not y-site administer ampicillin with midazolam, the placebo should not be either. Allowing co-administration of placebo with the midazolam because saline is compatible would unblind the nurse. Nurses are blinded so there should be no difference in administration technique between study arms

Study coordinators will be responsible for recording the time in the online database that study drug was both administered and stopped. This information can be obtained from bedside nurses notes in the patient’s file.

**3.11 Blinding and Unblinding**

Investigators, research team members, and NICU clinical staff (e.g. physicians, advanced practice nurses, nurses, respiratory therapists, social workers, case mangers) are blinded to treatment assignment. Pharmacists and pharmacy technicians involved in the preparation or documentation of investigational products (ampicillin, gentamicin, or placebo) are unblinded. Sites can have non-Investigational pharmacists (e.g. NICU pharmacists, Antibiotic Stewardship pharmacists) as blinded members of the clinical care team if they are not involved in investigational product preparation, documentation, or TDM, and if permitted by local standards. Unblinded data evaluation during the trial will be restricted to a designated study statistician (Dr. Jonathan Yabes, University of Pittsburgh) and the DSMB.

We will only unblind investigators and begin analyses after all data collection forms are completed, data queries resolved, and data are locked for analysis.

**Unblinding is a serious action and will be limited to reduce potential bias.**

**3.12 Study Measurements and Procedures**

To ensure that assessments and measurements are conducted consistently across study participants and clinical sites, this section describes the procedures for collecting samples and recording patient data into the online web-based database.

All supplies and procedures needed for sample collection will be provided by the CCC. It is the responsibility of each clinical site to alert the CCC if additional study kits are needed, before the next scheduled shipment. Shipments will occur every 2-3 months. The study kits for each mother/infant dyad include: 1 maternal stool kit, 1 maternal swab kit (vaginal and rectal), 1 infant blood kit and 8+ infant stool kits. Infant stool kit amount will vary based on the length of the infant’s stay in the NICU. Each study kit contains all of the necessary items to successfully collect samples needed (sample tubes/swabs, folders with Lab ID’s on the outside that contain specimen ID stickers inside to place on tubes/swabs, biohazard bags). Sites should refer to SOP’s provided by the CCC to collect maternal and infant samples.

**3.12.1 Sample Collection**

**Infant**

One spontaneously expelled fecal sample for microbiome analyses will be obtained weekly from study subjects up until 8 weeks of life. After 8 weeks of life, one spontaneously expelled fecal sample will be collected monthly. If a subject is diagnosed with NEC or LOS, additional stool samples may be requested, as available. Tubes will be provided by the CCC for sample collection. Once a fecal sample is collected, it should be labeled and placed in a -80˚C freezer until ready for shipment. The date of collection and type of sample (fresh or diaper) should be recorded on the Patient Specimen form by whoever collected the sample. If necessary, a sample can be placed in a -20˚C freezer overnight and moved to the -80˚C freezer the following morning. Fecal samples will be collected for the first 8 months of life or until discharge, whichever occurs first.

One blood draw for genetic analysis will be performed. This blood sample will be drawn simultaneously with clinical blood draws when possible. The volume of blood that will be drawn is 0.3 to 0.4 mL. Samples will be collected in EDTA tubes and shaken well prior to storage. After collection, the Patient Specimen form should be filled out indicating the date the sample was drawn. The blood draw can be performed from existing vascular access, venipuncture or heel stick. After the sample is collected, a label will be placed on the tube and it will be frozen at -80˚C until ready for shipment. If the blood draw is missed during clinical draws, this draw can be done during the first week of life. In the rare instance that a baby is having many clinically indicated blood draws, this blood draw can be drawn later when it is safe for the baby.

**Maternal**

Maternal intrapartum vaginal and rectal swabs will be collected at study sites that have infrastructure that allows them to do so. We recognize maternal collections will not be feasible at all study sites and in all cases, and therefore anticipate collecting maternal fecal and vaginal samples from about 25% of mothers participating in the study. Sites that can collect these samples will be designated before enrollment of their first patient.

For sites that have the infrastructure to collect an intrapartum vaginal and rectal swab: Clinical sites will determine who will be collecting these samples prior to enrollment of their first patient. Swab samples must be collected at the time of delivery. After obtaining samples, swabs should be placed in their protective container that is properly labeled and placed in a -80˚C until ready for shipment. A Patient Specimen form should be completed for each swab that was collected. If necessary, a sample can be placed in a -20˚C freezer overnight and moved to the -80˚C freezer the following morning.

If a vaginal and rectal swab cannot be obtained due to missed rectal sample or not having the proper infrastructure:

A postpartum maternal fecal sample (self-collected) will be collected. Ideally this fecal sample will occur during the first week postpartum. Once a fecal sample is collected, it should be labeled and placed in a -80˚C freezer until ready for shipment. A Patient Specimen form should be completed. If necessary, a sample can be placed in a -20˚C freezer overnight and moved to the -80˚C freezer the following morning.

Weekly sample collections do not need to occur on the same day every week. However, sample collection should not occur on consecutive days. SOPs for all sample collections (infant blood, infant stool, maternal vaginal and rectal swabs and maternal stool) will be distributed to all sites and should be followed. Training on sample collection SOPs will occur prior to enrollment of first patient.

**3.12.2 Data and Specimen Banking**

A single sample of blood from each study subject will be cryopreserved at the University of Pittsburgh for genotyping and/or exome sequencing at a later date. Samples will be stored indefinitely. They will be housed in the CRISMA Clinical Research Biospecimen Core Laboratory at Pitt. All sites will receive shipping supplies and instructions on how to properly ship samples back to the University of Pittsburgh for proper storage.

Infant stool samples and self-collected maternal stool samples will be collected, de-identified and stored for microbiome analyses. Maternal vaginal and rectal swabs will be collected by the obstetrics team and stored for paired microbiome analyses.

Cryopreserved patient samples will be de-identified such that no patient identifier information is accessible. A key of de-identified samples will be preserved on a secure password protected institutional server. Patient identifiers will be preserved in this protected environment until the study is completed, and then they will be de-identified.

De-identified patient samples may be released or studied only with the express written consent of the NANO Steering Committee.

All banked biological specimens will be stored indefinitely.

**3.12.3 Shipping of Samples**

Samples will be shipped to the University of Pittsburgh at predetermined time points (i.e. every 3 months). These time points will be site dependent and will be discussed prior to enrollment of first patient. It is the clinical sites responsibility to print return labels for shipping. Directions on ordering and printing return labels will be discussed prior to enrollment of first patient and can also be found in SOP 6.0.

Things to remember when shipping hazardous goods:

- Patient samples must be triple packed: a biohazard bag with an absorbent pad, a Styrofoam (or leakproof) container, a fiberboard box.
- Individuals shipping packages must have completed the appropriate training and be certified to ship dangerous goods through their institution.
  - If you have any questions about this, please reach out to the CCC coordinator. The University of Pittsburgh offers a *Dangerous Goods* training module, if needed.
- If shipping on dry ice, a dry ice label (class 9 diamond hazard label) must be affixed to the outside of the box with completed shipper/receiver information.
- If shipping biological substances (category B) a UN 3373 label must be affixed to the outside of the box.

**3.12.4 Tracking Samples**

Clinical sites will be responsible for logging collected samples and their associated specimen ID into the online database prior to shipping to the CCC. This ensures that all samples are accounted for and do not get lost. A patient specimen form (PSF) will be placed in each patient’s room where the date of sample collection, infant day of life (DOL) and specimen ID’s will be recorded.

An example of a specimen ID is **NA-IF-A-2__ __ __ __ __ - 2**. The breakdown of what each letter means is below:

- The first two letters (NA) stands for the NANO Trial
- The second set of two letters (IF) stands for the type of sample collected
- The third letter (A) stands for Aliquot letter
- The six-digit (2 _ _ _ _ _ ) number stands for the Lab ID
- The last digit (2) stands for the time point of sample

All patient’s enrolled in the NANO Trial will have a lab ID that is different than their study number. Maternal and infant Lab ID’s will also differ. The link between lab ID’s and the subject’s patient ID is stored in the online database.

**3.12.5 Data Collection**

Clinical site coordinators and additional research staff will be responsible for ensuring that all necessary patient data is collected and entered into the eDCF. To ensure proper use and understanding of the database, a PowerPoint training will be completed prior to receiving access to the database. Clinical site coordinators will also be responsible for completing and maintaining all mandatory source documents in the patient’s research record. Data should be recorded on all randomized patients. Infant data collection occurs until discharge.

Table 1 below summarizes the data to be collected and entered into the eDCF. All data should be entered in its entirety and with accuracy. There are checks in place within the database to ensure the completion of all required questions.

**Note**- Whenever there is a question in the eDCF about antibiotic days, coordinators should manually count number of days by referring to the patient’s chart.

| **Table 1. Data variables for data collection form** | | |
| --- | --- | --- |
| **Maternal** | **Demographics**- to be collected on all mothers screened | DOB, race, highest education level |
|  | **Lifestyle** | Tobacco & alcohol use, BMI |
|  | **Current pregnancy history** | Final antepartum admission date, number of fetus(es), prenatal obstetrics visit, history of spontaneous preterm birth |
|  | **Diagnoses during current pregnancy** (Yes/No) | Insulin-dependent diabetes, maternal hypertension, preeclampsia, antepartum hemorrhage, placenta previa, abruptio placenta, fetal growth restriction, cervical insufficiency, preterm labor |
|  | **Antibiotics administered within 30 days of delivery** | Diagnoses: (positive urine cultures, genital tract infection, skin/soft tissue infection, vaginal yeast infection, PPROM, GBS, presumed or confirmed intra-amniotic infection or chorioamnionitis); drug names, length of treatment |
|  | **Other drugs administered during current pregnancy** | Tocolytic therapy, betamethasone, antenatal corticosteroids; drug names, length of treatment |
| **Infant Baseline** | **Delivery** | Date, time, gestational age, sex, birth weight (kg) |
|  | **Randomization** | Date |
| **Infant Hospital Course** | **Outcomes at 1 week of age** | EOS diagnosis   1. If Yes: causative organism, antibiotics and dates of treatment   Days of antibiotics received during Week 1 of life |
|  | **Weekly** | Weight (kg), length (cm), LOS diagnosis (Yes/No), NEC diagnosis (Yes/No), death (Yes/No) |
|  | **Nutrition (Days 3, 7, 14, 28, 60)** | Enteral nutrition, type of milk received, date subject reached full enteral feedings |
|  | **Discharge** | Date of discharge, culture results, diagnoses: (Grade 3 or 4 IVH, ROP, CLD, days of endotracheal intubation, positive blood cultures, positive respiratory cultures, positive urine cultures, positive cerebrospinal fluid cultures) |
|  | **Antibiotics prescribed** | Diagnosis, drug name, total number of days administered |
|  | **IV Antifungal Medications** | Total number of days prescribed |

**3.12.6 Early Discontinuation**

No subject will be withdrawn from the study unless his/her parent(s) elects to withdraw the infant from further participation and notifies the study team accordingly.

For subjects that have been withdrawn from the trial, data, including samples obtained prior to the point of withdrawal can be used by the research team as originally described in the informed consent document.

**3.13 Safety Reporting**

As with any experimental procedure, there may be adverse events or side effects that are currently unknown and some of these unknown risks could be permanent, severe or life threatening. Major potential adverse events related to EA during the study will be captured and examined using standard reporting methodology. Research staff will strictly comply with IRB policies for the reporting of adverse events.

Definition of an adverse event (AE): Any unfavorable medical occurrence in human subjects, including abnormal signs, symptoms, or disease temporally associated with, but not necessarily considered related to, the subject’s participation in the research.

Definition of a serious adverse event (SAE): Any untoward medical occurrence during the index hospitalization that: (1) results in death, (2) is life-threatening, or (3) results in disability/incapacity.

All AEs and SAEs will be recorded from the time an infant is consented until discharged from the hospital. AEs and SAEs will be solicited from parents of subjects, attending physicians, and bedside nurses. The medical record will also be reviewed for the presence of adverse events.

An AE/SAE/UAP document along with reporting timelines can be found in Appendix 12.

**3.13.1 Reporting to the IRB**

AEs, SAEs and UAPs will be recorded promptly in the NANO database. Upon submission of an event, the CCC will notify the correct parties.

Timeliness of Reporting: Internal SAEs which are unexpected, fatal or life-threatening, and related or possibly Related to the Research Intervention must be reported to the IRB within 24 hours of the PI learning of the event.

All other internal unexpected AEs will be reported to the IRB within 10 working days of the PI learning of the event.

External SAEs that are Unexpected, Serious AND suggest that the research places subjects or others at greater risk than was previously recognized and Related to the Research Intervention will be reported to the IRB within 30 working days of their receipt by the University/UPMC investigator.

Unanticipated Problems (UAPs) Involving Risk to Subjects or Others and Non-compliance

1. UAPs which meet the following definition of “any accident, experience or outcome” that meets **all three** of the following criteria must be reported:

- unexpected in terms of nature, severity, or frequency;
- related, or possibly related, to a subject’s participation in the research;
- places subjects or others at a greater risk of harm (including physical, psychological, economic, or social harm) than was previously known or recognized;

2. Incidents of non-compliance (aka protocol deviations), which meet the following must be reported:

- Failure on the part of the investigator or any member of the study team to follow the terms of University of Pittsburgh IRB approved protocol or to abide by applicable laws or regulations, or University of Pittsburgh IRB policies that:
  - adversely affect the rights and welfare of human subjects, or
  - significantly compromises the quality of the research data

3. Incidents of non-compliance which are not required to be submitted to the IRB:

- Incidents of non-compliance which do not meet criteria for reporting should be logged in real time and should be available upon request.

Investigators are to submit all UAPs Involving Risks to Human Subjects or Others that are *Possibly* or *Definitely* Related to the research and incidents of reportable Non-compliance within 10 working days of the PI becoming aware of the reportable event/reportable new information.

**3.13.2 Reporting to IDSMB**

All SAEs and reportable UAPs/protocol deviations should be sent the DSMB Chair and IDSMB Coordinators via email within 24-48 hours of the PI learning of the event. The IDSMB will then forward it to the rest of the Board for review and comment. Documentation of the SAE should include de-identified source documents along with the corresponding de-identified patient summary. Documentation of UAPs will include as much information as provided to the PI.Those who will be notified of SAEs and UAPs include IDSMB chair, (benitzwe@stanford.edu), Susan Sandusky, (sls127@pitt.edu), and Cheryl Leow, (csl28@pitt.edu). After review of the SAE report, the DSMB chair may call an emergency meeting.

All other adverse events are to be recorded and logged within the online-database and reported quarterly on the AE report. The quarterly AE Report will be reviewed via email by the Board if a meeting is not being held.

Local Review Process: The P.I., the study coordinators and co-investigators will schedule quarterly meetings to review study updates, enrollment and overall study progression.

Each participating site will identify an Independent Safety Monitor (ISM): At each site, the ISM will be a neonatologist independent of the study team. The named ISM will be available to independently review all adverse events in real-time and have access to participant records to investigate those considered serious and unexpected. The site PI will alert the ISM of adverse events and will be given clinical laboratory data, clinical records and other study-related records if necessary. The ISM will be kept appraised of the study and all modifications.

**3.14 Study Compliance**

The NANO CCC will provide comprehensive training on the study protocol, data entry into the web-based database, track site performance and routinely communicate with the sites to help minimize protocol deviations.

The intervention consists of administering ampicillin and gentamicin at site approved dosing and duration OR a volume matched equivalent of normal saline. Antibiotic dosing and duration will be recorded when added to the University of Pittsburgh’s IRB. The study drug or placebo will be ordered and, in most cases, discontinued just as a sepsis evaluation would normally be ordered and discontinued; specifically, this means that the drugs will be ordered and administered within 4 hours of life and then discontinued when the attending neonatologist elects either to end or extend the course of antibiotics. Timing of drug administration will be closely monitored and recorded in the online database.

For all study subjects: time of randomization, entry of orders for study drug, drug dosage, drug administration, reporting of blood cultures, and discontinuation of study drug will be recorded. The CCC will promote compliance to the study intervention with regular feedback to each study site and identify solutions for rectifying non-compliance.

If a protocol deviation occurs, study coordinators will document this in a deviation log. In addition, if the study monitor discovers any of these deviations during a site visit, they will list any such occurrence in their monitoring report. Site investigators will follow their local IRB requirements for reporting protocol deviations to their local IRBs.

The Protocol Deviation log must be accessible at all times. This log can be found in Appendix 11.

**3.15 Data Collection and Study Forms**

**3.15.1 Source Documentation**

Information will be obtained by direct observation and by review of the EMR. Patient data collected will subsequently be entered into the eDCF by site coordinators and/or research staff. A subset of data may be requested later for data monitoring and quality control. When this occurs, the clinical site staff will pull the requested source documents, copy and send securely to the NANO CCC for review. All data will be kept for a minimum of 10 years.

Trained NANO personnel will have access to the online database to update eDCF forms using their unique username and password. Each user will be assigned specific permissions based on their role assignment within NANO. It is the site’s responsibility to ensure that all data are complete, intact, and transmitted to the CCC, through entry into the web-based database.

**3.15.2 Participant Binder**

All study documents will be retained by site investigators and will include clinical lab results and signed consent forms.

**3.16 Study Forms**

Data must be collected consistently across participants and sites so that any variability is limited to participants’ individual responses to the intervention. All data for the NANO Trial will be entered into the web-based database.

Data Correction Procedures

NANO patient data will be entered into eDCFs. Clinical site coordinators and/or additional research staff are strongly encouraged to check data before saving forms. If a mistake is caught after saving a form, the CCC should be contacted.

Sites are permitted to use paper DCF’s but must transfer data into online database on a weekly basis, preferably more frequently.

**3.17 Administrative Forms**

Administrative forms provide documentation of study processes and assist with study operations. The following forms will be kept at all clinical sites in a regulatory binder:

- Protocol
- Informed Consent Documents
- IRB Correspondence/Documentation
- Federal Wide Assurance Agreement (FWA)
- Qualification Documentation
- Delegation of Authority Log
- Protocol Deviation Log
- Accountability Records
  - Maintained in the Pharmacy by the unblinded research pharmacists.
- Record of Destruction of Clinical Product
- Monitoring Visit Reports/Logs
- Training Log
- Case Report Forms
- SOPs

**3.18 Data Safety and Management**

Data management will be coordinated by the CRISMA Center at the University of Pittsburgh. The data management system will be based on a PC platform, using a relational database server (SQL Server software) and Microsoft .NET framework. Enrollment and randomization data will be handled by a web-based system that uses Microsoft’s .NET web application. A complete backup of the server data is performed every day. SSL certification ensures encryption of data between site and the web server. All participant data will be entered by the site NANO study staff into the secure web-based data entry system via secure internet connection. Laboratory data will be maintained in a separate, secure location within the CRISMA Center at the University of Pittsburgh, with access limited only to laboratory personnel. Any identifying data will be maintained separately from clinical, laboratory and follow-up data for added security and confidentiality.

**3.19 External Data**

As part of the close-out procedure, the Data Coordinating Center (CRISMA BDMC at Pitt) will:

1) Perform a final data edit on the complete data set

2) Create a final data file

3) Perform statistical analyses that support the study findings

4) Archive the final data set

5) Finalize the program and data management system documentation

6) Issue final reports to the committees

7) Provide copies of the finalized datasets to NICHD and the investigative teams

The finalized dataset will be made public, as required, with the requisite documentation. The clinical trial investigators will be given the first and exclusive opportunity to analyze, present and publish data collected from their efforts. The study investigators will disseminate results and will write manuscripts including study design and primary results as well as other methodology and preplanned analyses of secondary outcomes supported actively by the BDMC. CRISMA BDMC staff is extremely familiar with preparing data and documentation for archives that make the data available for public use.

The data sharing plans also include sharing with the scientific community and ultimately the public. Upon request and prior to completion of the study, examples of data collection forms and instructions, the study protocol as well as other critical information (e.g., clear documentation of derived variables) will be made available to assist others in their developmental work of similar studies. This supports the overall concept of collaboration in the use of federal research dollars and may also provide the opportunity for investigators to be consultants or co-investigators. A data inventory will be kept so that others may use some information in planning their trials. We also recognize that NICHD may have additional needs for access to the data and/or special reports that may benefit the overall work of the Institute.

The study investigators strongly believe the breadth of the data collected through this application and potential uses of the data extend substantially beyond the interest and resources of the investigators specific to this trial. It is projected that within two years of publication of the primary results paper, an archived dataset with documentation will be made available for additional uses by outside investigators, in collaboration with the study investigators. We will work with program staff at NICHD in the development of a broad data sharing plan over time. Datasets will not contain personal identifying information on research participants. We will work with the NICHD to have the materials made publicly available while protecting the privacy of study subjects and their families.

The Publications and Presentations (P&P) policies and procedures for NANO can be modeled after those that have been used successfully in other studies. These will be developed, implemented, and enforced by the Publications Committee. This committee will be composed of the same individuals on the NANO Steering

Committee, but will be chaired by Dr. Polin. The P&P policies and procedures will be developed as part of the MOP and communicated to participating sites as part of the site initiation activities. It will also be available on the Trial website. All investigators will be encouraged to participate in opportunities for presentation and publications; resources within the BDMC will be available to facilitate this. These policies will provide for optimizing the use of the valuable data collected by the study and provide an additional non-financial incentive for participating investigators.

**3.19.1 Genomic Data Sharing**

The NANO Trial will also generate large-scale data sets containing de-identified bacterial DNA sequencing information. This will be in the form of 16S rRNA gene sequence data, which will be deposited in the Sequence Read Archive (SRA), the NIH’s primary archive of high-throughput sequencing data, after ensuring that no human sequence data are present. The data will be deposited at the time of data publication, or 6 months after the cleaned dataset is available, whichever occurs first. The SRA data will then be available to the research community. We will deposit the data with metadata (e.g. treatment arm) when possible to provide more information about the circumstances under which samples were collected. All deposited data and metadata will be de-identified to protect participants’ identities. Our algorithms will be made available through publications and as standalone programs when this is feasible (for example at GitHub). Our algorithms will be made available through publications and as standalone programs when this is feasible. All results will be available both as raw data, and as aggregate results, based on NIH policies.

**3.20 Retention of Study Documentation**

Maternal and infants identifiable medical record information will be used and shared until the study is completed in 2024. The link between maternal PHI and the study ID number will be destroyed 10 years after study completion. Records of the infant will be maintained until the child is 23 years of age. After that time, identifiers will be destroyed, and research data will be coded and retained anonymously indefinitely. Even though the information will still be used for research, it will no longer be possible to trace it easily to an individual and will be in secured long-term storage.

**3.21 Quality Control Procedures**

CRISMA’s BDMC will serve as the study Data Coordinating Center (DCC). The core of the trial data management and communications system will be the NANO web-based database. Sections with restricted access will be setup for members of the steering committee, and other individuals as needed. The data system area of the website will be the interface for data entry and data management.

**3.21.1 Standard Operating Procedures (SOPs)**

Standard operating procedures for NANO lab sample collection, processing, and shipment will be distributed to all sites.

**3.21.2 Site Monitoring**

Site monitoring will occur on an as needed basis. Sites that have minimal protocol deviations, high recruitment numbers and good communication with the CCC will be monitored less frequently. Sites that are struggling with any aspect of the trial will be asked to report back to the CCC more for better monitoring.

**3.21.3 Reports**

Reports comparing actual with expected recruitment will be developed for each site. Drop-out will also be monitored routinely. Protocol adherence reports will include enrollment of ineligible patients, follow-up data collection outside of protocol-defined windows, and important deviations from protocol. Reports will be provided to the sites, the Steering Committee, and the DSMB.

**3.22 Confidentiality Procedures**

All study-related information, including regulatory and source documents, will be stored securely at the study site after data is entered electronically into the eDCF. All participant information will be stored in locked areas with limited access. All laboratory specimens, reports, data collection, process, and administrative forms at the local study site will be identified by a coded identification number only to maintain confidentiality. Participant data will be entered by trained research personnel into the secure web-based database via secure internet connection. PHI will be collected directly from the research sites via the study’s web interface by the NANO coordinating center’s certified honest broker. This information will be encrypted using a standard algorithm during transmission. The encrypted data will be stored within the project database, which resides on the secure network drive, behind an enterprise firewall. Permission to access this database is restricted to the IRB approved personnel. These personnel will provide necessary PHI and/or related contact information to the NANO CCC.

**3.23 Data and Safety Monitoring Activities**

The University of Pittsburgh Office of Clinical Research, Health Sciences / CTSI will provide the logistical management and support of the DSMB. We propose to include neonatologists, maternal fetal medicine specialists, infectious disease specialists, and a statistician and/or epidemiologist experienced in the conduct of clinical research and unaffiliated with any study team members. Members will consist of persons independent of the investigators who have no financial, scientific, or other conflict of interest with the study. Written documentation attesting to absence of conflict of interest will be required. The DSMB will review the study protocol prior to study rollout and will meet on a pre-determined basis to review recruitment, retention, data completeness, protocol deviations, and adverse events. Safety data will be examined on an ad hoc basis if safety concerns arise from trial data or from external research or literature.

**William E. Benitz, MD** will be the Chairperson of the DSMB Board. This person will be the contact person for serious adverse event reporting. The DSMB has reviewed the final protocol before commencing enrollment and approved it. Meetings will be held every 6 months and at any other needed interval (based on reports) to ensure that no serious adverse consequences occur because of either administering or withholding the intervention. An emergency meeting of the DSMB will be called at any time by the Chairperson should questions of patient safety arise. DSMB reviews will consider the occurrence of adverse events, problems with loss of confidentiality, or other unanticipated problems, and will consider whether the anticipated benefit-to-risk ratio of study participation is altered by the findings in the safety monitoring process. Particular attention will be given to confirm that the study protocol is protecting the privacy of research subjects as anticipated.

The following will be reported to the IRB**:** Date of data and safety monitoring; summary of adverse event data including an assessment of intervention causality; summary of the assessment of relevant scientific literature and its impact on the design of the study; summary of procedural reviews conducted to ensure subject privacy.

This report will be accompanied by a conclusion regarding changes of the anticipated benefit-to-risk ratio and recommendations related to continuing, changing, or terminating the study. Recommendations to change the study will be accompanied with a detailed rationale for the proposed changes.

**3.24 Study Completion and Closeout Procedures**

This section of the MOOP outlines the study completion and close-out procedures.

- Verification that study procedures have been completed, data have been collected, and study intervention and supplies are returned to the CCC or prepared for destruction.
- Comparison of the investigator’s correspondence and study files against the Coordinating Center's records for completeness.
- Assurance that all data queries have been completed.
- Assurance that correspondence and study files are accessible for external audits.
- Reminder to investigators of their ongoing responsibility to maintain study records and to report any relevant study information to the NICHD.
- Assurance that the investigator will notify the IRB of the study’s completion and store a copy of the notification.
- Preparation of a report summarizing the study’s conduct.

**3.24.1 Participant Notification**

There are no plans to inform participants of results following the completion of the study.

**3.25 Publications and Access to Data**

The NANO Trial and its protocol outline have been submitted to clinicaltrials.gov. The Steering Committee plans to publish a trial methodology, manuscript and our statistical analysis plan.

Study results will be published in a PubMed referenced journal, regardless of study findings (i.e., whether a “positive” or “negative” trial). Plans for publication authorship will be equitable and likely take advantage of reporting as joint first or senior authors. All core, currently planned, manuscripts will be drafted and submitted by the NANO Publication Committee, led by the Principal Investigator from the University of Pittsburgh NANO Coordinating Center. The Publication Committee will invite NANO site collaborators with satisfactory enrollment, protocol adherence, and data quality to participate on the writing committee for each planned manuscript. The preferred authorship of core manuscripts is “The NANO Investigators”, with the Publication Committee noted and available from electronic searches.

The final NANO study dataset will include de-identified demographic and clinical information, and outcomes data. Protected health information or patient identifiers will not be part of any dataset. The recommended data documentation elements will be provided in accordance with NIH policy on sharing and releasing data. We will employ methods and procedures for sharing data that have been developed and utilized successfully with previous projects within our research group. Once the data are scrutinized for errors and validated, the data team will generate appropriate datasets in response to the specific needs of the proposed analysis. Documentation elements necessary to utilize the study data (e.g., data dictionary, study operating procedures) will also be shared via the project website. The website will be password-protected with access provided only to those who have received approval of their data request.

Basic study results will be reported in ClinicalTrials.gov within 12 months of trial completion. In compliance with NIH policy, resources developed through this project will be made readily available to investigators and data will be released completely and in a timely manner to facilitate use within the broader community. It is our plan to freely allow access to the study data as soon as the study is completed, and the main manuscripts published.

**3.26 MOOP Maintenance**

The MOOP will be maintained and updated throughout the study. The NANO CCC will have the responsibility of continuous review and quarterly audits and updates of the NANO MOOP. Each page of the MOOP will be numbered, dated and contains a version number to facilitate any changes and/or additions. The NANO MOOP will serve as a history of the project, documenting the time and nature of any changes in procedures and policies.

**Appendix 1: NANO Clinical Site Protocol, Version 4 dated April 2020**

***PROTOCOL TITLE:***

The NICU Antibiotics and Outcomes Trial (NANO)

**PRINCIPAL INVESTIGATOR:**

**Michael J. Morowitz, MD, FACS**

Associate Professor of Surgery

University of Pittsburgh School of Medicine

Attending Pediatric Surgeon, Division of Pediatric General and Thoracic Surgery

Children’s Hospital of Pittsburgh of UPMC

Rangos Research Center 6^th^ Floor

4401 Penn Avenue

Pittsburgh, PA 15224

(412) 692-5976

[Michael.morowitz@chp.edu](mailto:Michael.morowitz@chp.edu)

**VERSION NUMBER/DATE:**

Version 4/April 2020

**REVISION HISTORY**

| **Revision #** | **Version Date** | **Summary of Changes** | **Consent Change?** |
| --- | --- | --- | --- |
| 1 | 1/30/2020 | Updated statistical analysis, specified infant blood draw collected for genetic analysis, changed study drug dosing and duration, specified how patients with suspected infection will be treated | Yes |
| 2 | 3/20/2020 | Updated rescue antibiotic language and statistical analysis plan | No |
| 3 | 4/21/2020 | Updated Section 8.0 and updated languages that recruitment and consent documents will be available in | No |
|  |  |  |  |
|  |  |  |  |

**Table of Contents**

[1.0 Study Summary 3](#_Toc496162129)

[2.0 Objectives 4](#_Toc496162130)

[3.0 Background 4](#_Toc496162131)

[4.0 Study Endpoints 5](#_Toc496162132)

[5.0 Study Intervention/Investigational Agent 5](#_Toc496162133)

[6.0 Procedures Involved 6](#_Toc496162134)

[7.0 Data and Specimen Banking 11](#_Toc496162135)

[8.0 Sharing of Results with Subjects 12](#_Toc496162136)

[9.0 Study Timelines 12](#_Toc496162137)

[10.0 Inclusion and Exclusion Criteria 12](#_Toc496162138)

[11.0 Local Number of Subjects 14](#_Toc496162140)

[12.0 Recruitment Methods 14](#_Toc496162141)

[13.0 Withdrawal of Subjects 14](#_Toc496162142)

[14.0 Risks to Subjects 15](#_Toc496162143)

[15.0 Potential Benefits to Subjects 16](#_Toc496162144)

[16.0 Data Management and Confidentiality 16](#_Toc496162145)

[17.0 Provisions to Monitor the Data to Ensure the Safety of Subjects 22](#_Toc496162146)

[18.0 Provisions to Protect the Privacy Interests of Subjects 25](#_Toc496162147)

[19.0 Compensation for Research-Related Injury 25](#_Toc496162148)

[20.0 Consent Process 26](#_Toc496162150)

[21.0 Process to Document Consent in Writing 26](#_Toc496162151)

[22.0 Setting 26](#_Toc496162152)

[23.0 Resources Available 27](#_Toc496162153)

[24.0 Multi-Site Research 27](#_Toc496162154)

**1.0 Study Summary**

| **Study Title** | The NICU Antibiotics and Outcomes Trial (NANO) |
| --- | --- |
| **Study Design** | Randomized, placebo controlled, double blinded multicenter trial |
| **Primary Objective** | To compare the incidence of late onset sepsis (LOS), necrotizing enterocolitis (NEC), and death in premature infants ≤28 weeks gestation randomized to receive empiric antibiotics (EA) or placebo at birth. |
| **Secondary Objective(s)** | To compare early patterns of gut microbial colonization in premature infants ≤28 weeks gestation randomized to receive empiric antibiotics (EA) or placebo at birth. |
| **Research Intervention(s)/ Investigational Agent(s)** | The intervention consists of administering ampicillin and gentamicin at site approved dosing guidelines OR a volume matched equivalent of normal saline. |
| **IND/IDE #** | IND Exempt (PIND 143353) |
| **Study Population** | Newborn infants born at participating study sites with gestational age of 23-28 weeks |
| **Sample Size** | 802 |
| **Study Duration for individual participants** | Duration of index hospitalization |
| **Study Specific Abbreviations/ Definitions** | **ELBW-** Extremely Low Birth Weight Infants  **LOS**- Late Onset Sepsis  **NEC**- Necrotizing Enterocolitis: A disease that affects mostly the intestine of premature infants. The wall of the intestine is invaded by bacteria, which can cause local infection and inflammation that can destroy the intestinal wall.  **EA**- Empiric Antibiotics  **EOS-** Early Onset Sepsis  **NICU-** Neonatal Intensive Care Unit  **NIH-** National Institutes of Health  **AE-** Adverse Event  **SAE-** Serious Adverse Event  **IND**- Investigational New drug  **IDS**- Investigational Drug service  **IRB-** Institutional Review Board  **HHS-** Health and Human Services  **DSMB-** Data and Safety Monitoring Board  **MOP-** Manual of Procedures  **DCC-** Data Coordinating Center |

**2.0 Objectives and Aims**

The goal of the NANO Trial is to test the hypothesis that the rate of adverse outcomes is higher in ELBW infants receiving EA compared to infants receiving placebo. We have three aims:

Aim 1. To test the hypothesis that the composite incidence of LOS, NEC, and/or death is significantly different in infants that receive EA and infants that receive placebo.

Aim 2. To test the hypothesis that fecal samples in the first month of life from infants receiving EA will contain lower diversity, higher abundance of pathogens, and lower abundance of commensal anaerobes than fecal samples from infants receiving placebo.

Aim 3**.** (Exploratory) To identify microbial taxa associated with delayed or accelerated somatic growth (weekly weight and length z-scores) during the first month of life in infants receiving placebo or EA.

**3.0 Background**

Early onset sepsis (EOS) is a rare but morbid bloodstream infection that can occur in newborn infants during the first 3 days of life. Timely diagnosis of EOS is challenging in extremely low birthweight infants since there are no accepted biomarkers for the disease and because the clinical signs of EOS (e.g. respiratory failure) can overlap with common non-infectious physiologic derangements in preterm infants. As a result, the standard of care in many NICUs is to administer empiric antibiotics (EA) to preterm infants during the first 2-7 days of life or until a workup for EOS is complete^9–11^. Population-based studies have consistently shown that 80-90% of extremely low birthweight (ELBW) infants receive EA despite an EOS incidence of 2% or less^12–14^.

It is increasingly recognized that adverse effects of antibiotics extend beyond the well-recognized problem of antibiotic resistance to include unintended clinical consequences related to eradication of the human microbiota. Several recent studies in preterm infants have identified associations between early antibiotic exposure and adverse events including necrotizing enterocolitis (NEC) and late onset sepsis (LOS)^15–24^. For these reasons, antibiotic stewardship initiatives have been widely adopted in NICUs and indeed they have successfully reduced the length of EA therapy – however nearly all ELBW infants continue to receive at least a short course of EA at birth. It is generally accepted that published guidelines on the subject are outdated^25^, and therefore it is not surprising that EA practice patterns (e.g. length of therapy) vary wildly among providers and across centers^12,26–28^.

Our multidisciplinary team has more than 10 years of experience monitoring temporal changes in the gut microbiota of preterm infants^32–44^. In previous studies, we have documented that EA exposure is associated with unambiguous changes in the microbiota favoring growth of pathogens^10–23^. Our preliminary data supports published studies demonstrating a dose-dependent relationship between EA and a composite outcome of LOS, NEC, or mortality^45^. We and others have also described associations between antibiotics, gut bacterial colonization patterns, and infant growth^46,47^.

Completion of this trial may position us to conclude that EA therapy **worsens** outcomes or that EA **improves** outcomes in premature infants. If the former is true, results could rapidly be translated into a decrease in antibiotic usage in NICUs. Should we find *improved* outcomes with antibiotic administration, this trial would provide physicians with confidence to continue administering EA – a common practice at present despite a lack of high quality data. It is also possible that significant differences will **not** be observed between study arms and, depending upon the details, this result may itself be useful to care providers.

**4.0 Study Endpoints**

Primary Outcome: Composite incidence of adverse outcomes (NEC, LOS, or death during the index hospitalization).

NEC will be defined strictly by Bell’s stage II or III criteria for moderate or advanced NEC^50^. To reduce overlap between NEC and diagnosis of spontaneous intestinal perforation, the diagnosis of NEC will only be considered in infants > 7 days of age. LOS is **defined as** a positive blood culture obtained after 72 hours of life and intent to treat with antibiotics for 5 days or more^195^. Death is defined as death during the index hospitalization.

Secondary Outcomes: NEC during the index hospitalization, LOS during the index hospitalization, or death during the index hospitalization.

**5.0 Study Intervention/Investigational Agent**

Once maternal and infant eligibility are established, infants will be randomized into one of two different study groups: receiving conventional empiric antibiotics (ampicillin and gentamicin) or placebo while completing an evaluation for early onset sepsis. It is essential that randomization occurs within the first 4 hours of life.

All key resources for this proposal will be authenticated to enhance the reproducibility of our data, as appropriate and according to NIH policy (Notice Number: NOT-OD-17-068). Antibiotics used in this clinical trial will be commercially available pharmaceuticals sourced individually at each study site and administered to investigative pharmacy SOPs specific to each site. Lot numbers will be tracked to aid in identifying reagents that may be performing outside acceptable limits.

**6.0 Procedures Involved**

The NANO trial has been designed to study the longstanding clinical practice of empirically administering intravenous antibiotics to extremely low birthweight (ELBW) infants in the first days of life. It is an 802-subject multicenter placebo-controlled double-blinded randomized clinical trial to test the hypothesis that the incidence of adverse outcomes is higher in babies receiving EA in the first week of life compared to babies receiving placebo. We target a population of ELBW infants in whom the clinical decision to use or not use EA is currently most challenging -- infants that are clinically stable that did not have a known exposure to intraamniotic infection and were not born preterm for maternal indications.

6.1 Randomization and Blinding*.* For each infant born to a mother that has provided consent, eligibility will be assessed. One consent document is sufficient for both the mother and her baby. Thus, there will be a two-part screening to first determine maternal eligibility, and then to determine infant eligibility. IRB approved staff will screen infants based on inclusion/exclusion criteria. The site coordinator, or research staff, will discuss eligibility with approved staff and if the patient is eligible, input patient data into the web based system. Once data is inputted, research staff will randomize eligible families 1:1 using web-based block randomization stratified by study site to receive EA or placebo. Multiples (i.e. siblings) will be randomized to the same treatment arm. Staff will not see the treatment arm that patients are assigned to. Rather, treatment arm and patient information will be sent to each site’s investigational pharmacy, where study drug will be drawn and sent to the patient’s room. The staff member who performed randomization will see a confirmation message that randomization was successfully sent to the pharmacy. The investigational pharmacy is unblind to patient’s allocation. Participants, treating clinicians, and study staff will all be blinded to allocation. Randomization must occur within the first 4 hours of life. Outcome assessors and statistical summaries for trial monitoring will be unaware of group allocation. Unblinded data evaluation during the trial will be restricted to a designated study statistician and the DSMB. We will unblind investigators and begin analyses only after all data collection forms are completed, data queries resolved, and data are locked for analysis.

6.2 Study Procedure*.* The intervention consists of administering either conventional EA or placebo while completing an evaluation for early onset sepsis. Each site will source antibiotics individually. Either conventional EA or placebo must be given intravenously within 60 minutes following randomization. If EA is given, it will consist of ampicillin and gentamicin administered according to local, site-approved dosing guidelines clearly reflected in each site’s SOP. Infants randomized to receive placebo will receive volume-matched equivalents of normal saline matching the analogous schedule of ampicillin and gentamicin administration.

6.3 Rescue antibiotics. We anticipate that <<5% of study subjects will experience worsening of conditions prompting clinicians to order additional antibiotics that will be termed "rescue antibiotics." Clinicians will have complete freedom to repeat blood cultures and to order the antibiotics that they feel are most appropriate to treat the infant, with no restrictions imposed by the study protocol. This is a specific safety measure for NANO but is fully consistent with the standard of care for infants experiencing clinical deterioration. Should this occur, the treating physicians will remain blinded to the initial study drug assignment. The study pharmacist will be unblinded. Patients who have received a rescue option will continue to be followed until discharge from the NICU.

Rescue antibiotic therapy. There are two primary scenarios -- both rare -- that may be encountered requiring rescue antibiotic therapy. Should NICU providers wish to guarantee the infant actually receives ampicillin and gentamicin and not only placebo, a second set of study drug orders will be placed while the original study drug orders are discontinued. For this second set, if the original assignment was placebo, pharmacy will prepare ampicillin and gentamicin. If the original assignment was ampicillin and gentamicin, pharmacy will prepare ampicillin and placebo. The schedule for subsequent doses of antibiotics (if desired) would be determined by the team of providers based upon the timing of drug administration to date. The order and label should include some indication of set number to avoid preparation/dispensing/administration errors. If this rescue antibiotic scenario occurs, providers will have guaranteed that the infant is receiving ampicillin and gentamicin. Providers will not know what the initial order set consisted of, in order to maintain the integrity of the blind. The second set of orders should discontinue at the same time as the first round of orders.

The second rescue scenario that will arise in very rare circumstances will involve a decision by an Attending Neonatologist to empirically treat suspected/confirmed bacterial threats not optimally covered by ampicillin and gentamicin (e.g. penicillin for syphilis, ceftriaxone for gonorrhea, azithromycin for chlamydia, vancomycin or nafcillin for Staph, meropenem or cefepime for MDR GNR) with open label non EA antibacterials. These are ordered outside of the study and are not the responsibility of the IDS. Routine Candida prophylaxis (e.g. fluconazole) is not considered an open-label antibacterial for the purposes of this study and its use is not viewed as rescue therapy.

6.4 Early Discontinuation of EA. Study drugs can be discontinued at the discretion of the Attending Neonatologist prior to the completion of the study period, just as EA therapy might normally be discontinued outside the context of the trial. The Attending Neonatologist should ideally discuss their desire to discontinue study drugs early with the PI and research team before doing so. Potential reasons for discontinuing study drugs include:

•The Attending wants the participant to receive rescue antibiotic therapy (see above).

•They have decided that EA treatment is no longer necessary for clinical care (e.g. blood culture negative and no clinical concern for infection).

Ordering and administering antibiotics after the study period is at the discretion of the Attending Neonatologist. In such cases, NICU prescribers will place new orders for these antibiotics per routine care. IDS is not responsible for these antibiotics since they are beyond the study period and the participant has completed the study drug therapy. The NICU treatment team will decide their dosing and start times. A sensible approach would be to stay on the same schedule as the study drugs. If the participant was previously converted to blinded EA, the start time can be the split difference between the two sets’ schedules. Gentamicin plasma concentration monitoring may be used to help with determining start times (see next section). Prescribers will not be unblinded to determine these start times. They should remain blinded and should not count study doses towards the total days of therapy that the baby ultimately receives.

6.5 Gentamicin plasma concentration monitoring. It is expected that a small percentage of participants will receive gentamicin after the study period. This will trigger site-specific procedures for routine care gentamicin therapeutic drug monitoring, the results of which will may make it possible for blinded providers and staff to discern group allocation. This is expected and unavoidable. Pharmacists who routinely perform clinical pharmacokinetics in the NICU should therefore be part of the unblinded study pharmacist team. If allowed by site practice standards, clinical PK assessments that include dosing histories may be kept with the participant’s Dispensing Log rather than in their medical chart, in order to minimize the risk of unblinding.

6.6 Timeliness*.* Timeliness of the study intervention is of paramount importance and will be closely monitored. To facilitate proper implementation of the study intervention, it is the job of the coordinator to inform the attending physician, nurse practitioner and/or providers that consent has been given and randomization has occurred using an online system.

This protocol has been reviewed by the Food and Drug Administration (Department of Health and Human Services), and it was determined that an IND was not required to administer ampicillin or gentamicin as proposed for the NANO Trial.

6.7 Records and Data. Data to be obtained will include de-identified demographic and clinical information extracted from the medical record. Site coordinators will collect data by direct observation, chart review, and/or physician interview, and will enter data into a web-based form that minimizes burden and cost. Clinical data relevant to the study will be entered into this same database, and investigators will use only a specially assigned study number. An encoding table that links the study number to the subject’s name and medical record number within each institution will reside on a password-protected computer behind clinical firewalls.

Maternal and infant data variables to be collected are summarized in Table 1 below.

| **Table 1. Data variables for data collection form** | | |
| --- | --- | --- |
| **Maternal** | **Demographics**- to be collected on all mothers screened | DOB, race, highest education level |
|  | **Lifestyle** | Tobacco & alcohol use, BMI |
|  | **Current pregnancy history** | Final antepartum admission date, number of fetus(es), prenatal obstetrics visit, history of spontaneous preterm birth |
|  | **Diagnoses** (Yes/No) | Insulin-dependent diabetes, maternal hypertension, preeclampsia, antepartum hemorrhage, placenta previa, abruptio placenta, fetal growth restriction, cervical insufficiency, preterm labor |
|  | **Antibiotics administered within 30 days of delivery** | Diagnoses: (positive urine cultures, genital tract infection, skin/soft tissue infection, vaginal yeast infection, PPROM, GBS, presumed or confirmed intra-amniotic infection or chorioamnionitis); drug names, length of treatment |
|  | **Other drugs** | Tocolytic therapy, betamethasone, antenatal corticosteroids; drug names, length of treatment |
| **Infant Baseline** | **Delivery** | Date, time, gestational age, sex, birth weight (kg) |
|  | **Randomization** | Date |
| **Infant Hospital Course** | **Outcomes at 1 week of age** | EOS diagnosis   1. If Yes: causative organism, antibiotics and dates of treatment   Days of antibiotics received during Week 1 of life |
|  | **Weekly** | Weight (kg), length (cm), LOS diagnosis (Yes/No), NEC diagnosis (Yes/No), death (Yes/No) |
|  | **Nutrition (Days 3, 7, 14, 28, 60)** | Enteral nutrition, type of milk received, date subject reached full enteral feedings |
|  | **Discharge** | Date of discharge, culture results, diagnoses: (Grade 3 or 4 IVH, ROP, CLD, days of endotracheal intubation, positive blood cultures, positive respiratory cultures, positive urine cultures, positive cerebrospinal fluid cultures) |
|  | **Antibiotics prescribed** | Diagnosis, drug name, total number of days administered |
|  | **IV Antifungal Medications** | Total number of days prescribed |

6.6 Sample Collection.

**Infant**

1-2 spontaneously expelled fecal samples for microbiome analyses will be obtained weekly from study subjects up until 8 weeks of life. After 8 weeks of life, 1 spontaneously expelled fecal sample will be collected monthly. If a subject is diagnosed with NEC or LOS, additional stool samples may be requested, as available. An SOP will be distributed to all sites to ensure proper collection of fecal samples.

An additional research blood sample for genetic analysis will be drawn one time and should be done at the time of clinical blood draws. However, if this blood draw is missed, it can be done in the neonate's first week of life. A volume of 0.3 to 0.4mL will be drawn in EDTA tubes and shaken well. After the sample is collected, it will be frozen for shipment. The blood draw will be performed by NICU personnel who routinely draw blood on preterm babies. It will be either the bedside nurse or the respiratory therapist depending on whether the blood is drawn from an umbilical catheter or by heelstick.

**Maternal**

Intrapartum vaginal and rectal swabs will be collected on mother’s at sites that have infrastructure that allows them to do so. We recognize maternal collections will not be feasible at all study sites and in all cases, and therefore anticipate collecting maternal fecal and vaginal samples from 25% of mothers participating in the study. Sites that can collect these samples will be designated before enrollment of their first patient. An SOP will be distributed to all sites to ensure proper collection of these samples.

If a vaginal and rectal swab cannot be obtained, a postpartum maternal fecal sample (self-collected) may be collected. If a rectal swab is missed at sites that plan on collecting maternal swabs, a postpartum fecal sample will be collected. Samples will be obtained exclusively for research purposes, and there will be no testing of patients beyond obtaining stool samples and recording demographic data and clinical history. An electronic database will be used to track sample collection and storage history.

6.8 Follow Up. Currently, there are no plans for follow-up.

6.9 Consent. Sites will develop an SOP to identify women as early as possible that are admitted to participating hospitals and expected to deliver an infant at or before 28 weeks gestation. If eligible, and if the attending obstetrician provides permission, then trained research personnel will explain the research protocol and the process of informed consent. Research staff will also have the option of showing an IRB approved video to parents. This video has been created to provide a brief background about the trial, discuss the procedures involved and touch upon the risks associated with participating in this trial. This video is not required to be shown at sites but is recommended. Informed consent requires that the mother of the study subject understand the details of the study and agrees, without coercion, to participation in the study. Study representatives at each site will introduce and explain the study to the mothers and present them with the detailed consent form to read and review. It will be the responsibility of the site investigator to ensure that the mother is given full and adequate verbal and written information about the nature, purpose, benefit, and the potential risks of the study. A copy of signed informed consent document will be provided to the mother and another will be placed in the medical chart. The original document will be placed in the study files. Permission for study participation will be obtained from one parent. If there are multiples, a consent document must be obtained for each child.

**7.0 Data and Specimen Banking**

A single sample of blood from each study subject will be cryopreserved at The University of Pittsburgh for genotyping and/or exome sequencing at a later date. They will be housed in the CRISMA Clinical Research Biospecimen Core Laboratory at Pitt. All sites will receive shipping supplies and instructions on how to properly ship samples back to the University of Pittsburgh for proper storage.

Infant stool samples and self-collected maternal stool samples will be collected, de-identified and stored for microbiome analyses. Maternal vaginal and rectal swabs will be collected by the obstetrics team and stored for paired microbiome analyses.

Cryopreserved patient samples will be de-identified such that no patient identifier information is accessible. A key of de-identified samples will be preserved on a secure password protected institutional server. Patient identifiers will be preserved in this protected environment until the study is completed, and then they will be de-identified.

De-identified patient samples may be released or studied only with the express written consent of the NANO Steering Committee.

All banked biological specimens will be stored indefinitely.

**8.0 Sharing of Results with Subjects**

Parents of study subjects will not be informed of results of genetic or microbiome analyses that may be performed on samples collected from their children.

**9.0 Study Timelines**

The duration of study participation for each individual study subject will be identical to the duration of his/her index NICU hospitalization. Enrollment in the NANO Trial is anticipated to begin in 2020 and conclude in 2024. Primary analyses of study data are anticipated to be completed by 2025.

Major annual milestones are also listed here:

- **Year 1:** IRB approval at the coordinating center and all study sites; completion of web-based data collection form; negotiation and execution of contracts between study sites; completion of two pre-launch site visits; initial study rollout.
- **Year 2:** Study launched at all sites; 30% enrollment completed; data from first interim analysis sent to DSMB; microbiome sequencing and analysis begins.
- **Year 3:** Data from second interim analysis sent to DSMB after 60% enrollment completed.
- **Year 4:** 90% enrollment completed.
- **Year 5:** 100% enrollment completed; completion of data querying and cleaning; data lock achieved; microbiome analysis completed; submission of abstracts/manuscripts for publication; submission of final study report to NICHD.

**10.0 Inclusion and Exclusion Criteria**

Labor and delivery antenatal units will be screened for all admissions of mothers with pregnancies ≤28 weeks GA.

10.1 Inclusion/Exclusion Criteria.

Participant Inclusion Criteria:

- Newborn infants born with gestational age of 23-28 weeks
- Inborn infants at participating study sites
- Mothers of babies (23-28 weeks) that were not approached prenatally may be approached within 4 hours of delivery if they are inborn and do not present with any maternal or infant exclusion criteria

Participant Exclusion Criteria:

- Infants at low risk for EOS – born for maternal indications via caesarean section with ROM within 6 hours of delivery, no attempts to induce labor, and no concern for maternal infection
- Infants at high risk of EOS born to mothers with intrapartum fever (> 38ºC) or clinical diagnosis of chorioamnionitis (suspected or definite)
- Infants with respiratory insufficiency requiring invasive mechanical ventilation and FiO_2_ > 0.40 or non-invasive ventilation (i.e. CPAP) and FiO_2_ > 0.60 at time of randomization
- Infants with ongoing hemodynamic instability requiring vasopressors or more than one fluid bolus at time of randomization
- Clinician concern for sepsis due to physical exam findings, e.g. lethargy
- Major congenital anomalies
- Infants not anticipated to survive beyond 72 hours
- Infants who have received antibiotics prior to randomization
- Mothers that are <18 years old at time of enrollment

10.2 Study Restriction. As detailed in the text, study participation will be restricted to premature newborns and sample collection will be limited to the first months of life. This will enable us to test the hypothesis that EA increases the incidence of adverse outcomes after premature birth. For this reason, it is necessary to limit our study to this specific patient demographic. Prisoners and institutionalized women are excluded from the study.

10.3 Screening Log. A screening log provides documentation of all individuals that are reviewed for study eligibility. NANO sites will be instructed to maintain patient screening logs and submit the information via the online database. This will allow the coordinating center to review efforts and barriers regarding enrollment. Study sites are permitted to keep a paper screening log.

Items included in the NANO subject screening log include:

- Maternal age
- Race
- Screening date
- Did not meet inclusion criteria
- Did meet exclusion criteria
- Reasons for not screening (staff unavailable, consent refusal and other reasons)

**11.0 Local Number of Subjects**

We anticipate 40% of enrollments to occur at The University of Pittsburgh, around 325 patients, with the remainder distributed relatively even across the additional study sites.

**12.0 Recruitment Methods**

Sites will develop protocols to identify women as early as possible that are admitted to participating hospitals and expected to deliver an infant at or before 28 weeks gestation. Trained research personnel will identify and discuss informed consent with parent(s) of potential study subjects. Because variable amounts of time will have elapsed between consent and delivery, research staff will meet briefly on a regular basis with parents that have provided antenatal consent. If antenatal consent is not possible, research staff will seek postpartum consent as clinical circumstances permit. For each infant born to a mother that has provided consent, the coordinator will confirm eligibility of the infant with IRB approved staff. Thus, there will be a two-part review to first determine maternal eligibility, and then to determine infant eligibility. Only infants meeting eligibility criteria and consented for participation will be enrolled.

Our strategy to maximize enrollment and retention of study subjects relies upon continual communication with parents and providers of study subjects. We will adopt site-specific and centralized strategies designed to optimize retention. At the time of informed consent and subsequently, we will strive to explain the details and the timeline of the study protocol as clearly as possible. We will encourage parents of study subjects to contact research coordinators and/or site PIs with questions. We will also attempt to be as flexible as possible regarding the timing of sample collections, respecting the privacy of NICU families and respecting the limited time and availability of NICU nursing staff. As often as possible, we will strive to convey to NICU providers and families that the motivation for this trial is to minimize antibiotic exposure and to improve outcomes for preterm infants. Barriers to retention of infants should be minimal as the period during which research data are being collected is limited to the index NICU hospitalization.

Labor and delivery antenatal units will be screened for all admissions of mothers admitted with pregnancies ≤28 weeks GA.

There is no reimbursement (payment) for participating in this study.

**13.0 Withdrawal of Subjects**

No subject will be withdrawn from the study unless his/her parent(s) elects to withdraw the infant from further participation and notifies the study team accordingly.

For subjects that have been withdrawn from the trial, data, including samples obtained prior to the point of withdrawal can be used by the research team as originally described in the informed consent form.

**14.0 Risks to Subjects**

14.1 Potential risks*.* The trial may be associated with the following risks. Mechanisms to protect against these risks are specified below and will be detailed in the study consent document.

1. Risk of not receiving EA therapy. As shown in our preliminary data, many or most ELBW infants currently receive EA after delivery. **We do not know if outcomes for these infants will be improved without antibiotics. The study is designed to answer this question.** As with any placebo controlled trial, it is possible that participants will be harmed by receiving placebo instead of receiving active treatment. In this trial, the use of a placebo is in accordance with the Declaration of Helsinki since the efficacy and safety of EA therapy have not been clearly established. Because the study targets infants that are not critically ill, we believe that the risk of withholding antibiotic therapy is low. Further, subjects receiving placebo will not be subject to additional risks of serious or irreversible harm as a result of receiving placebo rather than antibiotics, since the trial will not hinder subjects from receiving any care or interventions necessary for their protection. Accordingly, both the plan for statistical analysis and the MOP anticipate and account for the small percentage of subjects that will be converted from EA to an extended, therapeutic course of antibiotics.
2. Risk of receiving EA therapy. Studies suggest that the following major medical problems may be significantly more likely in premature infants that receive antibiotics at birth when they do not demonstrate signs of a blood stream infection: blood stream infections after 3 days of life, side effects from the antibiotics that include; skin rashes, hives, mild gastrointestinal upset with changes in stool, temporary changes in kidney function and rarely hearing loss, severe intestinal problems (i.e. Necrotizing enterocolitis), serious long-term breathing problems, serious long-term eye problems and abnormal brain development. These side effects are usually seen when high doses of the antibiotics have been ordered or the drugs were given for an extended period of time.
3. Risk from collection of fecal samples and vaginal and rectal swabs. Infant stool samples will be collected by the bedside nurse from spontaneously expelled feces discovered in infant diapers. Maternal fecal samples will be self-collected. Maternal vaginal and rectal swabs will be collected by obstetricians during delivery. There are no known risks to infants or their mothers associated with these collections.
4. Risk from collection of blood samples. If heel stick, then the added blood draw may very slightly increase the risk of bruising due to the addition time of squeezing the heel and sometimes even needing a second “prick”. If by venipuncture or through a line, then the risk is negligible since the risks are already assumed by the clinical lab draw.
5. Risk of loss of confidentiality. There is a risk of breach of confidentiality related to the demographic and clinical data that has been recorded. In order to minimize this risk, all records pertaining to subject identifiable data will be stored in a locked file cabinet in the office of research staff and/or on password-protected computers behind a firewall. All study personnel who have contact with potential participants or data will have completed a course on human subjects’ protection that covers the importance of maintaining confidentiality. The PHI information collected for the purposes of this research study will be assigned a research study code and any personal identifiers will be removed from this information. Personal identifiers will not be attached to research data. All subject information will be handled in compliance with HIPAA.

**15.0 Potential Benefits to Subjects**

Both the published literature and our preliminary data suggest that infants randomized to the placebo may enjoy improved health outcomes relative to infants that receive empiric antibiotics. These possible benefits include but are not limited to: 1) decreased incidence of NEC, LOS, and death, 2) improved postnatal growth, and 3) decreased incidence of common morbidities among preterm infants, e.g. retinopathy. We therefore believe it is plausible that the benefits of study participation will outweigh the risks of study participation.

**16.0 Data Management and Confidentiality**

The hypothesis of the primary outcome is that the incidence of composite adverse events (NEC, LOS, or death) is significantly different in ELBW infants receiving EA and infants receiving placebo. Based on 1,000,000 simulations for the group-sequential test for comparing two proportions and O’Brien-Fleming alpha spending method for two interim analyses, we need 382 infants in each arm to reach 90% power to test the hypothesis of the primary outcome using a two-sided significance level of 0.05. We base our event rates for the standard of care (i.e. infants receiving EA) on published literature regarding risk of adverse events with each day of antibiotics, on recent data from the Vermont Oxford Network ^51^, and upon data from the Pediatrix CDW. We used 13.5% and 22% for the placebo and EA groups, respectively in the sample size calculations. The effect size (odds ratio 1.35 *per day of* EA) is based on the scant literature available to assess the risk-benefit ratio of EA. Another assumption made in the calculation includes intraclass correlation (ICC) of 0.01. Based on in-house data for babies born at Magee-Womens Hospital in the past 4 years, we estimate that 20 out of 100 infants will be twins or triplets. Anticipating a 5% attrition rate, we will recruit 802 infants (from ~670 families; 401 infants in each arm) to reach at least 90% power.

Given recent admission numbers and anticipated moderate growth at NANO study sites, we estimate that the 7 sites will admit 2700 infants ≤28 weeks GA over a 4-year period, allowing 6 months for trial rollout and 3-6 months in Year 5 for final analyses and preparation of manuscripts. We estimate that 1755 infants (65% of total) will meet eligibility criteria.  Based upon our surveys of recently delivered mothers (see above) and our experience with NICU RCTs^173,174,176^, we believe it is reasonable to expect that parents of 30-50% of these 1755 infants will elect to participate. A 46% consent rate will yield approximately 807 study subjects (29.9% of total admissions ≤28 weeks GA).  If the rate of missing outcome data is 5%, then the trial will have 766 subjects – yielding 383 patients per group. This randomized clinical trial will thus have excellent power to detect clinically meaningful differences between the EA and placebo groups with respect LOS/NEC/Death. If the consent rate is lower than 46%, we will add enrollments at 1-2 additional sites.

We will compare the distribution of baseline variables between study arms to assess randomization success. To summarize continuous variables, we will use means and standard deviations or medians and interquartile range, and will use frequencies and percentages for categorical variables. Graphical methods (e.g., histogram, boxplot) will be used for assessing the overall shape of a continuous variable. No formal statistical hypothesis tests will be performed to avoid unnecessary testing.

The primary analysis is an intent-to-treat (ITT) analysis that includes two interim analyses at 1/3 and 2/3 enrollment, and a final analysis using O'Brien-Fleming stopping rules. The primary test for the primary outcome is that the **incidence of composite adverse events is significantly different in ELBW infants receiving EA compared to infants receiving placebo**.

The primary outcome will be analyzed using a generalized linear model (GLM) with a log link fitted via generalized estimating equations (GEE) with exchangeable working correlation matrix and employ robust variance estimates. This will account for non-independence of observations due to clustering of infants within families. The exchangeable correlation was selected primarily due to parsimony. Furthermore, infants within a twin or triplet can be considered exchangeable (i.e., the assigned infant ID within a set is arbitrary). The model will include treatment as fixed effects adjusted for site and gestational age. The primary hypothesis will be tested via the Wald test of the treatment assignment and effect estimates will be presented using risk ratios (RR) with 95% confidence intervals (CI).

In secondary analyses, we will examine each component (NEC, LOS, death) of the composite adverse outcome separately using the same analytic approach as the primary outcome.

*Microbiome analysis*. For microbiome analyses in **Aims 2 and 3**, assuming that the alpha diversity is normally distributed, we need a total samples size of 78 over two groups (39 per group) to detect a 43% reduction in alpha diversity (from 1.75 to 1) at the 14 day timepoint with a 5% level of significance and 90% power. This effect size is based upon our preliminary data and also published reports, as described in the Research Strategy. We will easily exceed power to discern differences in alpha diversity but will plan for much larger sample size to allow not only for taxonomic analyses across samples from EA and placebo groups, and also subgroups (as defined above for Aim 1 analysis).

Bacterial 16S rRNA gene sequences will be extracted from infant samples, amplified, and sequenced on the Illumina Miseq according to established protocols used routinely by the Morowitz, Gregory, and Peddada laboratories. In analyses of these samples, we shall investigate three important parameters, namely, the alpha diversity (Richness and Shannon Index), the beta diversity, and the differential abundance of individual bacterial taxa. We hypothesize that samples from EA subjects will contain increased abundance of pathogens (e.g. *Enterococcaceae* and *Enterobacteriaceae)* and decreased abundance of commensal anaerobes (e.g. *Bacteroidaceae* and *Bifidobacteriaceae*) relative to samples from infants receiving placebo.

**For Aim 2**, bacterial 16S rRNA gene sequences from weekly fecal samples collected during the first month of life from each experimental group will be compared at each time point and also with longitudinal trend analyses. Temporal trend analysis of alpha diversity will be performed with the Constrained Linear Mixed Effects (CLME) models package developed by the Peddada group. The analysis does not make any distributional assumptions, and takes into account the repeated measurement feature in the data since longitudinal measurements are obtained on each subject. The method is also robust against heteroscedasticity as it is based on Best Linear Unbiased Predictor (BLUP) residual bootstraps. The temporal trend analysis of beta diversity will be performed using an extension of PERMANOVA for repeated measurements data. As for temporal differential abundance analyses within each experimental group, we will use the newer version of ANCOM for detecting trends in abundance, while allowing repeated measurements as well as covariates. ANCOM was developed by Peddada and colleagues, and is based on the software ORIOGEN developed by Peddada. Note that differential abundance analyses of taxa between groups cannot be performed using standard ANOVA or t-test because these data reside inside a simplex. Currently, among the many methods used in the literature for differential abundance analyses, ANCOM is the only method that controls the false discovery rate at the desired nominal level.

We will measure the importance of clinical factors known to impact the infant gut microbiota, including mode of delivery, gestational age, diet, and maternal antibiotic exposure while remaining blinded to the identity of treatment assignment groups. In each case, we shall perform pairwise comparisons of individual variables (e.g. vaginal delivery vs. caesarean section) after adjusting for the remaining factors. Initially, we will consider diet (formula milk vs. human milk and maternal vs. donor milk) as a binary variable, but ultimately may perform more granular analyses regarding percentage of calories from each type of milk. Similarly, we will initially consider gestational age (23-26 weeks vs. 27-28 weeks) and maternal antibiotic exposure (yes/no) in pairwise comparisons, but later could consider gestational age as a continuous variable or could distinguish between classes of maternal antibiotics. We will also incorporate microbiome analyses of maternal vaginal swabs and early postpartum fecal samples, but anticipate such samples for only 25% of study subjects.

**For Aim 3 (exploratory)**, weight, length, and head circumference Z-scores will be calculated for birth and weekly postnatal growth measurements using Fenton and Olsen growth curves for preterm infants. Using the CLME methodology, we shall (a) develop nonparametric temporal growth curve model for each group of babies and (b) compare the temporal differences in growth curve patterns between the two groups of babies. The methodology will adjust for various confounders in the study. Furthermore, as noted earlier, CLME is entirely nonparametric as it does not make any distributional assumptions, robust to heteroscedasticity and does not rely on any parametric shape of the growth curve. Secondly, we shall also perform high dimensional regression analysis by regressing growth at time “t” on the vector of OTU counts from the previous time point using LASSO type high dimensional regression methodology used by us in Bertelsen et al.^191,197^. By including an interaction in the model between OTUs and experimental groups, we hope to detect taxa that are differentially associated with delayed and accelerated growth patterns in the two groups. The methodology will not only identify taxa that are associated with growth but will also provide statistical significance of the selected taxa.

The University of Pittsburgh will be the home of the NANO DCC (CRISMA BDMC) and the central IRB (Pitt HRPO). The DCC will be responsible for assuring the standardization, collection, management and quality control of the data as well as the statistical design and analysis of the study. The DCC will monitor the data from all sites.

16.1 CRISMA’s BDMC. CRISMA will serve as the study Data Coordinating Center (DCC). The core of the trial data management and communications system will be the project website, which will include a shared document section and a data system area. The website will include a personnel directory, project calendar, and shared documents. Sections with restricted access will be setup for members of the steering committee, and other individuals as needed. The data system area of the website will be the interface for data entry and data management. Reports comparing actual with expected recruitment will be developed for each site. Drop-out will also be monitored routinely. Protocol adherence reports will include enrollment of ineligible patients, follow-up data collection outside of protocol-defined windows, and important deviations from protocol. Reports will be provided to the sites, the Steering Committee, and the DSMB.

They will monitor all aspects of study performance (e.g., enrollment, data processing time) and protocol compliance (e.g., randomization), as well as adherence to established adverse event reporting and event adjudication procedures. They will regularly provide reports to the clinical site coordinators addressing scheduling and delinquency. The project manager and PIs will conduct data monitoring site visits per a predetermined plan, prioritizing sites where specific data issues are identified or data concerns arise (risk-based monitoring).

16.2 Online Portal. The center of the NANO trial’s communications system will be a portal accessible via a password-protected study website which will be maintained by the DCC. The site will be used for day-to-day communications among the NANO study team by providing immediate communication, effective collaboration and project management. Access will be restricted to study investigators, research staff and committee members via unique usernames and passwords. The DCC’s password policy is consistent with National Institute of Standards and Technology (NIST) password policy. The primary interface is a collection of organized and individual web features, each representing a single tool which will vary in presentation and availability based on a defined user’s role (e.g., investigator, clinical site coordinator) and group association(s) (e.g., clinical site, committee membership).

16.3 Missing data. Despite best efforts to obtain follow-up data, we anticipate some loss-to-follow-up. We will describe the extent and reasons that data are missing, summarizing the proportion of patients with missing data for each outcome and by study arm and by site. We will compare baseline patient characteristics between those who have complete outcome data and those that do not. It is anticipated that, on rare occasion (<<5% of subjects), even before culture results are received, an attending neonatologist caring for a study subject will elect to prolong antibiotic therapy beyond 72 hours (e.g. due to clinical deterioration). This will be classified as a protocol deviation. Such infants will be analyzed according to intention to treat principles.

Optimal ITT includes analysis of data from all subjects randomized and cannot be directly adopted in the presence of missing data. To resolve this, we chose multiple imputations to conduct the ITT analyses. Multiple imputed datasets will be generated using multivariate imputation by chained equations, an approach designed for multivariate data that can accommodate mixed data types. We will use predictive mean matching and logistic regression to impute continuous and binary outcomes, respectively. We will generate 100 imputed datasets to maintain power, although 3-5 imputed datasets are usually sufficient to obtain excellent results^196^. We assume that missing outcome data are missing-at-random (MAR) in that they can be imputed reasonably well from the observed study data. We will perform imputation based on the study arm to which the patient was assigned. Auxiliary variables for the imputation model will include patient baseline variables (e.g., age, sex, race, and study site).

16. 4 Sensitivity analyses. We will assess the robustness of study findings to assumptions about missing data, the primary analysis population, and covariates used for adjustment. Specifically, we will perform the following:

- We will conduct a missing-not-at-random (MNAR) sensitivity analysis using control-based imputation in which all data are imputed based on the placebo arm.
- Since ITT generally biases towards no difference, we will perform a per-protocol analysis in which the complier average causal effect (CACE) will be estimated by using the treatment assignment as an instrumental variable.
- Baseline variables that were found to be strongly imbalanced between the treatment groups will be added as adjustment variables to the primary analysis model.

16.5 Data storage and security. Initial stages of data management will be done by experienced clinical data managers, database developers, data research associates, and data processing staff, supported by computer system analysts, programmers, and information technology specialists. These persons will be responsible for data quality and timeliness, documentation of processes and procedures, and training of data management staff.

Data will be entered directly into a secure, backed-up, 24-hour, web-based database using electronic forms developed by the BDMC staff with the assistance of project investigators and statisticians. Data entry screens will incorporate range and logical edit checks, both within and across forms. A data monitoring plan written before the start of data collection, will serve as a reference guide for the development of case report forms, data handling conventions, reporting, data dictionaries, supporting meta data, as well as project closeout activities, communication and coordination plans among the PIs, clinical teams, sites coordinators, and staff and faculty-level statisticians.

Identifiable medical record information will be used and will remain at each site until the study is completed in 2025. PHI information will not be shared across sites.

The link between your PHI and the study ID code will be destroyed ten years after study completion. Records of your baby will be maintained until he/she is 23 years of age. After that time, identifiers will be destroyed, and research data will be coded and retained anonymously indefinitely.

**17.0 Provisions to Monitor the Data to Ensure the Safety of Subjects**

17.1 Safety monitoring and event reporting. As with any experimental procedure, there may be adverse events or side effects that are currently unknown and certain of these unknown risks could be permanent, severe or life threatening. We will capture the major potential adverse events related to EA during the study and examine each adverse event using standard reporting methodology. Research staff will strictly comply with IRB policies for the reporting of adverse events. To assist with reporting of adverse events, an event table will be distributed to all sites prior to enrollment.

*Definition of an adverse event (AE).* Any untoward medical occurrence in a subject temporally associated with the trial protocol, whether or not it is considered causally related to the trial protocol. Examples include: (1) significant or unexpected worsening or exacerbation of the condition under study, (2) or new conditions detected or diagnosed after protocol initiation even though it may have been present prior to the start of the study.

*Definition of a serious adverse event (SAE).* Any untoward medical occurrence during the index hospitalization that: (1) results in death, (2) is life-threatening, or (3) results in disability/incapacity.

All AEs and SAEs will be recorded from the time of randomization until hospital discharge. AEs and SAEs will be solicited from parents of subjects, attending physicians, and bedside nurses; the medical record will also be reviewed daily for the presence of events. Notification of coordinating center regarding any SAE will take place within 48 hours of recognition. The coordinating center will then be responsible for notifying the DSMB regarding all SAEs within 48 hours. When an AE/SAE occurs, it will be the site PI’s responsibility to review the pertinent records, notes, laboratory, and radiographic data. This information will be recorded along with the site PI’s impression of the diagnosis. The site PI will assess causality between the event and the study protocol using best clinical judgment, and this will be reviewed by the DSMB who will recommend if follow-up or modification of the study protocol is necessary. All deaths will be reviewed by the DSMB within 30 days of reporting said event.

17.2 Data safety and monitoring board (DSMB). The University of Pittsburgh Office of Clinical Research, Health Sciences / CTSI will provide the logistical management and support of the DSMB. A letter of support can be provided to sites, if needed. We propose to include neonatologists, maternal fetal medicine specialists, infectious disease specialists, and a statistician and/or epidemiologist experienced in the conduct of clinical research and unaffiliated with any study team members. Members will consist of persons independent of the investigators who have no financial, scientific, or other conflict of interest with the study. Written documentation attesting to absence of conflict of interest will be required. The DSMB will review the study protocol prior to study rollout and will meet on a semi-annual basis to review recruitment, retention, data completeness, protocol deviations, and adverse events. Safety data will be examined on an ad hoc basis if safety concerns arise from trial data or from external research or literature.

The DSMB will:

1. Review the research protocol, informed consent documents and plans for data and safety monitoring;
2. Evaluate the progress of the study, including periodic assessments of data quality and timeliness, participant recruitment, accrual and retention, participant risk versus benefit, adverse events, unanticipated problems, performance of the trial sites, and other factors that can affect study outcome;
3. Consider factors external to the study when relevant information becomes available, such as scientific or therapeutic developments that may have an impact on the safety of the participants or the ethics of the study;
4. Review clinical center performance, make recommendations and assist in the resolution of problems reported by the PI;
5. Protect the safety of the study participants;
6. Report on the safety and progress of the study;
7. Make recommendations to the PI, and if required, to the NIH / NICHD concerning continuation, termination or other modifications of the study based on the observed beneficial or adverse effects of the treatment under study;
8. Monitor the confidentiality of the study data and the results of monitoring;
9. Assist the PI by commenting on any problems with study conduct, enrollment, sample size and/or data collection.

The first meeting will take place before study initiation to discuss the protocol, approve the commencement of the study, and to establish guidelines to monitor the study. A safety officer (Chairperson) will be identified at the first meeting. This person will be the contact person for serious adverse event reporting. The DSMB will review the final protocol before commencing enrollment and then meet semi-annually and at any other needed interval (based on reports) to ensure that no serious adverse consequences occur because of either administering or withholding the intervention. An emergency meeting of the DSMB will be called at any time by the Chairperson should questions of patient safety arise.

DSMB reviews will consider the occurrence of adverse events, problems with loss of confidentiality, or other unanticipated problems, and will consider whether the anticipated benefit-to-risk ratio of study participation is altered by the findings in the safety monitoring process. Particular attention will be given to confirm that the study protocol is protecting the privacy of research subjects as anticipated. The following will be reported to the IRB: date of data and safety monitoring; summary of adverse event data including an assessment of intervention causality; summary of the assessment of relevant scientific literature and its impact on the design of the study; summary of procedural reviews conducted to ensure subject privacy. This report will be accompanied by a final conclusion regarding changes of the anticipated benefit-to-risk ratio and recommendations related to continuing, changing, or terminating the study. Recommendations to change the study will be accompanied with a detailed rationale for the proposed changes.

17.3 Interim analyses and stopping rules. The NANO Coordinating Center will conduct two interim analyses and a final analysis using O’Brien and Fleming stopping rules defined *a priori* controlling for an overall Type I error rate of 0.05. The first and second interim analyses will be scheduled at approximately 1/3 and 2/3 enrollment, respectively. The DSMB will review recruitment, safety, data collection, and analysis results at each interim analysis. Before trial completion, only the DSMB and a designated study statistician will have access to unblinded data. **During the interim assessments, the DSMB could recommend early trial termination due to unanticipated safety concerns, or due to one or more of the following reasons:**

1. A significant difference is found for the study hypothesis and a Z test statistic significance bound is crossed because of excess incidence of a composite adverse outcome (NEC, LOS, or death) in the EA arm.
2. A significant difference is found for the study hypothesis and a Z test statistic significance bound is crossed because of excess incidence of a composite adverse outcome (NEC, LOS, or death) in the placebo arm.
3. Failure to obtain success in the implementation of the trial either through failure to accrue subjects at the necessary rate, improper data handling, or inability to implement study protocols.

**18.0 Provisions to Protect the Privacy Interests of Subjects**

18.1 Risk of loss of confidentiality. The risk of loss of confidentiality will be minimized by de-identifying patient samples such that no patient identifier information is accessible and preserving a key of de-identified samples which is password protected on a secure institutional server. Hardcopies of consent forms will be kept in locked offices. Patient identifiers will be preserved in this protected environment until the study is completed, and then they will be de-identified. A copy of the consent form will also be added to subject’s medical chart as well as a separate notation in EMR that they are a participating in the NANO trial. Non-identifiable data including microbiome data will be preserved until all analysis is complete. Data collection forms will be accessed via a secure web-based data entry system with access limited to study personnel only. The data will be transferred to the study database server via a secure internet connection. Database access is limited to the NANO data management team in accordance with state and federal regulations.

Parents of potential study subjects will be encouraged to take as much time as needed to ask questions and feel comfortable deciding whether to allow their children to participate or not.

**19.0 Compensation for Research-Related Injury**

Emergency medical treatment for injuries solely and directly related to a study subject’s participation in this research study will be provided by the hospital participating in NANO caring for the particular study subject. Insurance providers may be billed for the costs of this emergency treatment, but none of those costs will be charged directly to study subjects or their families. If a subject’s research-related injury requires medical care beyond this emergency treatment, his/her family will be responsible for the costs of this follow-up care. At this time, there is no plan for any additional financial compensation. Subjects and their families do not give up any legal rights by agreeing to participate in the NANO Trial.

**20.0 Consent Process**

Sites will develop a protocol to identify women as early as possible that are admitted to participating hospitals and expected to deliver an infant at or before 28 weeks gestation. If eligible, and if the attending obstetrician provides permission, then trained research staff will explain the research protocol and the process of informed consent. Informed consent requires that the parents of the study subject understand the details of the study and agree, without coercion, to participation in the study. Study representatives at each site will introduce and explain the study to the parents and present them with the detailed consent form to read and review. It will be the responsibility of the site investigator to ensure that each parent is given full and adequate verbal and written information about the nature, purpose, benefit, and the potential risks of the study. A copy of signed informed consent document will be provided to the parents and another will be placed in the medical chart. The original document will be placed in the study files. Participation for study participation will be obtained from one parent. If there are multiples, a consent document must be obtained for each child.

Child assent for this trial involving newborn infants will not be possible.

Oral and written information provided to parents of possible study subjects will be available in Spanish and other languages, as needed.

**21.0 Process to Document Consent in Writing**

For each infant enrolled in NANO, documentation of informed consent and study enrollment will be entered into the official medical record.

**22.0 Setting**

NANO study sites are university affiliated birthing hospitals that have participated in clinical trials involving preterm infants and/or their mothers. They have been selected for participation based upon clinical research experience, projected recruitment, infrastructure that will enable study protocol execution, and geographic diversity. We expect each site to recruit 2-3 subjects monthly, allowing for completion of study enrollment in approximately 4 years.

**23.0 Resources Available**

Each NANO study site is equipped with clinical, laboratory, and health records systems that will enable prompt identification of subjects, randomization, and delivery of study drug or placebo. Additional sites may be added during the trial planning period. Primary study activities of subject enrollment and randomization will occur across all sites according to a single study protocol. Each site will have a lead investigator, and together they will ensure that the protocol and trial is implemented as designed. Given recent admission numbers and anticipated moderate growth at NANO study sites, we estimate that study enrollment can be completed within a 4-year period, allowing 6 months for trial rollout and 3-6 months in Year 5 for final analyses and preparation of manuscripts.

**24.0 Multi-Site Research**

Collectively, NANO study sites will enroll **802 inborn infants** delivered in the obstetrical facilities of participating NANO study sites, and their mothers. The NIH Single IRB policy applies to this study, and we have identified no exceptions. As indicated in the attached letter, the Pitt HRPO will serve as the central IRB for the NANO trial, including the lead center, the data coordinating center, and those selected or added after award. Sites will sign a reliance agreement that will include a communication plan. All participating sites will agree to rely on the designated single IRB, without exception. All proposed study sites are SmartIRB centers, which will facilitate finalization of agreements between institutions. The project manager at The University of Pittsburgh, under supervision of Dr. Morowitz and Dr. Polin, will provide applicable conflict of interest management plans for relying site study teams to the Pitt HRPO, and will provide confirmation to the Pitt HRPO that relying site study teams have completed relevant training and are qualified to conduct the proposed research. The project manager will also provide documentation of IRB determinations to relying site study teams, and provide copies of IRB-approved materials to the lead study team. Additional responsibilities of the Pitt team will include providing the consent form template to relying site study teams, obtaining and collating study wide information for continuing review to the Reviewing IRB. Dr. Polin will coordinate interactions with the Data and Safety Monitoring Board, will lead all reviews of protocol deviations, and will report reportable events (e.g., unanticipated problems, noncompliance, subject complaints) to the Pitt HRPO in conjunction with Dr. Morowitz.

24.1 Communication. **Effective communication is the single most critical factor to achieve the goals of any clinical trial.** The project management strategy for NANO includes tools and techniques that place emphasis on communicating at the appropriate time with highly pertinent information and as widely as possible to all appropriate study personnel. A communication plan will be developed in the first phase of the study that identifies each stakeholder group, their communication needs, the facilitator, and the preferred frequency and methods of communication. The plan will also set the standard for how and when study information will be shared. Recognizing the importance of communication with all members of the NANO investigative team, the other investigators and clinic settings will have both regularly scheduled meetings with the PIs, and be encouraged to communicate via email and unscheduled calls as needed.

A major portion of the communication plan includes meetings and conference calls. For meetings and conference calls, standardized agendas and meeting minutes will be used to aid consistent communication. All minutes will include a list of agenda items, action items and follow-up of action items from previous meetings. The supporting documentation will be provided to committee members before each meeting with enough lead-time to allow for additional input.

The center of the NANO trial’s communications system will be a portal accessible via a password-protected study website which will be maintained by the DCC. The site will be used for day-to-day communications among the NANO study team by providing immediate communication, effective collaboration and project management. Access will be restricted to study investigators, research staff and committee members via unique usernames and passwords. The DCC’s password policy is consistent with National Institute of Standards and Technology (NIST) password policy. The primary interface is a collection of organized and individual web features, each representing a single tool which will vary in presentation and availability based on a defined user’s role (e.g., investigator, clinical site coordinator) and group association(s) (e.g., clinical site, committee membership).

24.2 Document Library*.* The Document Library will be used to store operations memos, data collection forms, manuals, training materials, and manuscripts so that study personnel have easy access to these materials. The Document Library area will have restricted access to individual libraries as needed.

24.3 Help Center. A Help Center/Frequently Asked Questions (FAQ) section of the website will be used to assist Co-investigators and Coordinators when answers are needed to questions, such as, clarification of inclusion/exclusion criteria or particular aspects of the study protocol. The web-based Help Center will list the most frequently accessed FAQ entries. A search dialog will permit the researcher to find other FAQ entries based on keywords. If the FAQ database does not sufficiently aid in finding an answer to a question, a direct link to the Help Request form is available. The Help Request form may be completed by the researcher and submitted online. Upon submission, an email notification will be sent to the appropriate DCC and/or CCC personnel. Once a request is resolved, the requestor will receive an email verifying the solution.

24.5 Data Management System. A link to the Data Management System will be accessible to study personnel who have appropriate authorization. This area will provide access to the data management system for data entry, verification, error correction, data tracking, reporting and randomization. In order to prevent unauthorized access to incoming data while in transit, this area of the website will use a server certificate to encrypt all incoming traffic over a secure channel using Secure Sockets Layer (SSL).

**Appendix 2. Historical Summary of NANO Protocol Changes**

| **IRB Approval Date** | **Protocol Version** | **Description of Modification(s)** |
| --- | --- | --- |
| 03/03/2020 | Version 2, Dated 01/2020 | - Updated statistical analysis plan. - Included additional blood draw for infant (additional 0.3 to 0.4 mL taken at time of clinical draws, when possible) for genetic analysis. Updated consent document to reflect this. - Updated study drug dosing and duration to state “site approved dosing guidelines” – this allows each site to dose as they typically would. Each site will designate dosing in SOP. - Updated Section 6.3 to address study drug and implementation of rescue antibiotics. - Removed data collection table. Created new data table to reflect updated DCF’s. - Included language to allow sites to show a supplemental IRB approved informational video to potential participants prior to consent. This video will not take the place of the informed consent process. - Updated the language for withdrawal. |
| 04/01/2020 | Version 3,  Dated 03/2020 | - Updated statistical analysis plan based on feedback from DMSB - Updated language to reflect how rescue antibiotics will be discontinued and dispensed if the treating physician wants an Ampicillin/Gentamicin guarantee |
| 05/19/2020 | Version 4,  Dated 04/2020 | - Updated Section 8.0 - Updated languages that recruitment and consent document will be available in |

**Appendix 3: Coordinating Center Study Roster**

**Principal Investigator:**

**Michael J. Morowitz, MD, FACS**

Associate Professor of Surgery

University of Pittsburgh School of Medicine

Attending Pediatric Surgeon, Division of Pediatric General and Thoracic Surgery

Children’s Hospital of Pittsburgh of UPMC

Rangos Research Center 6^th^ Floor

(412) 692-5976

Michael.morowitz@chp.edu

**Co-Investigators:**

**Toby Debra Yanowitz, MD, MS**

Associate Professor of Pediatrics

Division of Newborn Medicine

University of Pittsburgh School of Medicine

(412) 641-6260

yanotd@upmc.edu

**Liza Konnikova, MD, PhD**

Assistant Professor in Pediatrics

Children’s Hospital of Pittsburgh of UPMC

liza.konikova@chp.edu

**Jennifer L Kloesz, MD**

Professor of Pediatrics

UPMC Magee-Womens Hospital

(412) 641-4111

kloejl@upmc.edu

**Joanne Duara**

duarajl@upmc.edu

**Kinsey Roth**

kinsey.roth2@chp.edu

**Kathleen Schwabenbauer**

schwabenbauerks@upmc.edu

**Brighid O’Donnell**

odonnellbm@upmc.edu

**Bianca Loverde**

loverdebe@upmc.edu

**Pallavi Karunakaran**

Pallavi.karunakaran@upmc.edu

**Alice Randall**

randallp@upmc.edu

**Thomas Hooven**

hooventa@upmc.edu

**Chelseà Johnson**

Chelsea.johnson2@chp.edu

**Danielle Browning**

browningdn@chp.edu

**Karena Lawrence**

karena.lawrence@chp.edu

**John Ibrahim**

john.ibrahim@upmc.edu

**Kalyani Vats**

VATSKR@upmc.edu

**Stacy Beck, MD**

Becks2@upmc.edu

Maternal Fetal Medicine

**Christina Megli, MD, PhD**

meglicj@upmc.edu

Maternal Fetal Medicine

**Project Coordinator/Project Manager**

**Alyssa M Harris, MS**

Department of Pediatric Surgery

Children’s Hospital of Pittsburgh of UPMC

4401 Penn Avenue

Pittsburgh, PA 15224

(412) 692-8003

Harrisam4@upmc.edu

**Investigative Drug Services**

Mary Beth Pasqualicchio, R.PH.

Investigational Drug Pharmacist

UPMC Magee-Womens Hospital

300 Halket Street

Suite 4670

Pittsburgh, PA 15213

pasqmb@upmc.edu

(412) 641-2523

**Appendix 4: Clinical Site Contact Information**

**Brigham & Women’s Hospital**

| **Address/Phone number** | **PI** | **Site Coordinator** | **IRB Coordinator** | **Pharmacy Contact** |
| --- | --- | --- | --- | --- |
| Boston, MA | Katherine L. Gregory, MD  Kgregory1@bwh.hardvard.edu | Jennifer Filatava  efilatava@bwh.harvard.edu | Maria Sundquist  msundquist@partners.org | Kevin Anger  kanger@bwh.harvard.edu  Alka Patel  apatel11@bwh.harvard.edu  Caitlin Grant  cgrant@bwh.harvard.edu  bwhrxids@partners.org  (617) 732-6410 |

**Morgan Stanley Children’s Hospital**

| **Address/Phone number** | **PI** | **Site Coordinator** | **IRB Coordinator** | **Pharmacy Contact** |
| --- | --- | --- | --- | --- |
| NYC, NY | **Co-Investigator**  Richard A. Polin, MD  Rap32@cumc.columbia.edu  Noa Fleiss, MD  Nof9012@nyp.org | Caitlin Ehret  [Ce2310@cumc.columbia.edu](mailto:Ce2310@cumc.columbia.edu)  Kathleen Overman  [ko102@cumc.columbia.edu](mailto:ko102@cumc.columbia.edu) | Tasha Smith  Ts2257@cumc.columbia.edu | Connie Eng  ce2166@cumc.columbia.edu  Marta Scotto  mis2121@cumc.columbia.edu  Lucy Liu  ll2840@cumc.columbia.edu  (212) 305-9867 |

**Norton Children’s Hospital**

| **Address/Phone number** | **PI** | **Site Coordinator** | **IRB Coordinator** | **Pharmacy Contact** |
| --- | --- | --- | --- | --- |
| Louisville, KY | Tamina Singh, MD  Tamina.singh@louisville.edu | Kristen Gossett  Kristen.lee@louisville.edu | Sarah King  Sarah.penny@louisville.edu  (502) 629-2721  Christy LaDuke  Christy.laduke@louisville.edu  (502) 852-5188 | Ivy Tiu  ivy.tiu@nortonhealthcare.org  Sarah Smith  Sarah.Smith@nortonhealthcare.org  (502) 629-5568 |

**Children’s Hospital of Philadelphia (CHOP)**

| **Address/Phone number** | **PI** | **Site Coordinator** | **IRB Coordinator** | **Pharmacy Contact** |
| --- | --- | --- | --- | --- |
| Philadelphia, PA | Eric Eichenwald, MD  EICHENWALD@email.chop.ed | Megan Dhawan, CRNP  DhawanM@email.chop.edu | Amy Schwarzhoff, MS, MBA, CIP  Schwarzhoffa@email.chop.edu  (267) 426-2346 | N/A |

**Hospital of the University of Pennsylvania (HUP)**

| **Address/Phone number** | **PI** | **Site Coordinator** | **IRB Coordinator** | **Pharmacy Contact** |
| --- | --- | --- | --- | --- |
| Philadelphia, PA | Eric Eichenwald, MD  EICHENWALD@email.chop.ed | Megan Dhawan, CRNP  DhawanM@email.chop.edu | Patrick Stanko, BA, CIP  pstanko@penn.edu  (215) 573-2197 | Arleen Kessler  Arleen.Kessler@pennmedicine.upenn.edu  Investigational Pharmacy  pennIDS@pennmedicine.upenn.edu |

**Pennsylvania Hospital (PAH)**

| **Address/Phone number** | **PI** | **Site Coordinator** | **IRB Coordinator** | **Pharmacy Contact** |
| --- | --- | --- | --- | --- |
| Philadelphia, PA | Karen Puopolo, MD, PhD  karen.puopolo@pennmedicine.upenn.edu | Toni Mancini, RN  Toni.mancini@pennmedicine.upenn.edu  (732) 773-5900 | Patrick Stanko, BA, CIP  pstanko@penn.edu  (215) 573-2197 | Erin Ticehurst  Erin.Ticehurst@pennmedicine.upenn.edu |

**Sharp Mary Birch Hospital for Women & Newborns**

| **Address/Phone number** | **PI** | **Site Coordinator** | **IRB Coordinator** | **Pharmacy Contact** |
| --- | --- | --- | --- | --- |
| San Diego, CA | **Co-Investigator**  Anup Katheria, MD  Anup.Katheria@sharp.com  (858) 939-4170  David Kaegi  David.kaegi@sharp.com  (858) 939-4171 | Katie Baker  Katherine.baker@sharp.com  (858) 939-4113 | Marcie Portillo  Marcie.Portillo@sharp.com  (858) 939-4112 | **Lead Pharmacist**  Jason Sauberan  jason.sauberan@sharp.com  (858) 939-7424  (858) 939- 4299 |

**Thomas Jefferson University Hospital**

| **Address/Phone number** | **PI** | **Site Coordinator** | **IRB Coordinator** | **Pharmacy Contact** |
| --- | --- | --- | --- | --- |
| Philadelphia, PA | Zubair Aghai, MD  Zubair.aghai@nemours.org | Margaret Lafferty  Margaret.lafferty@nemours.org | Crystal Lijadu  Crystal.Lijadu@jefferson.edu | Braden Rall  IDS-service.pharmacy@jefferson.edu  (215) 955-6923 |

**University Hospital**

| **Address/Phone number** | **PI** | **Site Coordinators** | **IRB Coordinator** | **Pharmacy Contact** |
| --- | --- | --- | --- | --- |
| San Antonio, TX | J.B Cantey, MD  cantey@uthscsa.edu | Diana Anzueto  anzuetod@uthscsa.edu | Brandie Otten  otten@uthscsa.edu  (210) 567- 9251 | Armando Garcia  Armando.GarciaJr@uhs-sa.com  (210) 743-4033 |

**University of Kansas Hospital (KUMC)**

| **Address/Phone number** | **PI** | **Site Coordinator** | **IRB Coordinator** | **Pharmacy Contact** |
| --- | --- | --- | --- | --- |
| Kansas City, KS | Marc Parrish, DO  mparrish@kumc.edu | John Moore  Jmoore20@kumc.edu  (913) 588-6287 | Melody Solace  Msolace3@kumc.edu  (913) 588-1493 | Leslie Curtis  Lcurtis@kumc.edu  (913) 945-7621  (913) 588-7756 |

**Children’s Mercy Hospital**

| **Address/Phone number** | **PI** | **Site Coordinator** | **IRB Coordinator** | **Pharmacy Contact** |
| --- | --- | --- | --- | --- |
| Kansas City, MO | Howard Kilbride, MD  hkilbride@cmh.edu  Venkatesh Sampath, M.B.B.S  vsampath@cmh.edu | Cheri Gauldin  cagauldin@cmh.edu | Amanda Matthews  ajmatthews@cmh.edu | Investigational Drug Services  IDSPharmacy@cmh.edu  816-983-6645 |

**University of Virginia Children’s Hospital**

| **Address/Phone number** | **PI** | **Site Coordinator** | **IRB Coordinator** | **Pharmacy Contact** |
| --- | --- | --- | --- | --- |
| Charlottesville, VA | David Kaufman, MD  dak4r@hscmail.mcc.virginia.edu | Monika Thielen  MJT3C@hscmail.mcc.virginia.edu | Monika Thielen  MJT3C@hscmail.mcc.virginia.edu | Amy Adam  apa4n@virgina.edu  (434)-982-1048 |

**UPMC Magee-Women’s Hospital**

| **Address/Phone number** | **PI** | **Site Coordinator** | **IRB Coordinator** | **Pharmacy Contact** |
| --- | --- | --- | --- | --- |
| Pittsburgh, PA | Michael J. Morowitz, MD, FACS  Michael.morowitz@chp.edu  (412) 692-5976 | Alyssa Harris  Harrisam4@upmc.edu | Allison Gerger  Irb.reliance@pitt.edu  (412) 383-1981 | Lynn Mitterer  mitterel@upmc.edu  Mary Beth Pasqualicchio  pasqmb@upmc.edu  Debbie Zowacki  zowackidl@upmc.edu  (412) 641-6626 |

**Yale New Haven Children’s Hospital**

| **Address/Phone number** | **PI** | **Site Coordinator** | **IRB Coordinator** | **Pharmacy Contact** |
| --- | --- | --- | --- | --- |
| New Haven, CT | Matthew Bizzarro, MD  matthew.bizzarro@yale.edu | Christine Henry  christine.henry@yale.edu  (203) 390-9148  Taryn Zamary  Taryn.zamary@yale.edu  (203) 688-2320 | Taryn Zamary  Taryn.zamary@yale.edu  (203) 688-2320 | Jing Lu  jing.lu@ynhh.org  (203) 688-4872 |

**Appendix 5: Steering and Publication Committee Members**

**Committee Chair:**

Michael J. Morowitz, MD, FACS

Michael.morowitz@chp.edu

(412) 692-5976

**Committee Members:**

Anup Katheria, MD

Anup.Katheria@sharp.com

Richard A. Polin, MD

Rap32@cumc.columbia.edu

David Huang, MD, MPH, FACEP, FFCM

huangdt@ccm.upmc.edu

Alyssa Harris, MS

Harrisam4@upmc.edu

Elizabeth Pace, MD

paceek@upmc.edu

Joyce Chang, PhD

changj@pitt.edu

Jason Sauberan, PharmD

Jason.Sauberan@sharp.com

Jonathan Yabes, PhD

Jgy2@pitt.edu

Shymal Pedadda, PhD

Sdp47@pitt.edu

**Appendix 6: NANO Dispensing Example and Log**

| **Participant Study ID #:** 1320001 | **Randomization Arm** (check one)**: □ drug / □ placebo** | **Dosing weight (kg):** 0.8 |
| --- | --- | --- |
|  | Date starting blinded EA (if applicable): |  |

| **Date**  **(MM/DD/YY)** | **Ampicillin** | | | **Saline** | | | | **Gentamicin** | | | | **Saline** | | | **Initials** |
| --- | --- | --- | --- | --- | --- | --- | --- | --- | --- | --- | --- | --- | --- | --- | --- |
|  | **Syringe #** | **Mfr, Lot #** | **Dose (mg/mL)** | **Syringe #** | | **Mfr, Lot #** | **Dose (mL)** | **Syringe #** | **Mfr, Lot #** | **Dose (mg/mL)** | | **Syringe #** | **Mfr, Lot #** | **Dose (mL)** |  |
| 02/04/20 | - | - | - | 1 | | Hospira 62-158-DK | 1.3 | - | - | - | | 1 | Hospira 62-158-DK | 0.32 | JL |
| 02/05/20 | - | - | - | **2** | | **same** | **same** |  |  |  | |  |  |  | **MBP** |
| 02/05/20 | - | - | - | **3** | | **same** | **same** |  |  |  | |  |  |  | **IT** |
| 02/06/20 | - | - | - | **4** | | **same** | **same** |  |  |  | |  |  |  | KA |
|  |  |  |  |  | |  | SAMPLE |  |  |  | |  |  |  |  |
|  |  |  |  |  | |  |  |  |  |  | |  |  |  |  |
|  |  |  |  |  | |  |  |  |  |  | |  |  |  |  |
|  |  |  |  |  | |  |  |  |  |  | |  |  |  |  |
|  |  |  |  |  | |  |  |  |  |  | |  |  |  |  |
|  |  |  |  |  | |  |  |  |  |  | |  |  |  |  |
| **Participant Study ID #:** | | | | | **Randomization Arm** (check one)**: □ drug / □ placebo** | | | | | | **Dosing weight (kg):** | | | |  |
|  |  |  |  |  | Date starting blinded EA (if applicable): | | | | | |  |  |  |  |  |

| **Date**  **(MM/DD/YY)** | **Ampicillin** | | | **Saline** | | | **Gentamicin** | | | **Saline** | | | | **Initials** | |
| --- | --- | --- | --- | --- | --- | --- | --- | --- | --- | --- | --- | --- | --- | --- | --- |
|  | **Syringe #** | **Mfr, Lot #** | **Dose (mg/mL)** | **Syringe #** | **Mfr, Lot #** | **Dose (mL)** | **Syringe #** | **Mfr, Lot #** | **Dose (mg/mL)** | | **Syringe #** | **Mfr, Lot #** | **Dose (mL)** | |  |
|  |  |  |  |  |  |  |  |  |  | |  |  |  | |  |
|  |  |  |  |  |  |  |  |  |  | |  |  |  | |  |
|  |  |  |  |  |  |  |  |  |  | |  |  |  | |  |
|  |  |  |  |  |  |  |  |  |  | |  |  |  | |  |
|  |  |  |  |  |  |  |  |  |  | |  |  |  | |  |
|  |  |  |  |  |  |  |  |  |  | |  |  |  | |  |
|  |  |  |  |  |  |  |  |  |  | |  |  |  | |  |
|  |  |  |  |  |  |  |  |  |  | |  |  |  | |  |
|  |  |  |  |  |  |  |  |  |  | |  |  |  | |  |

**INSTRUCTIONS:**

1. One log per study participant.
2. May use multiple pages per participant if needed, but should normally only need one page since study duration is 36-48 hrs.
3. Participant Study ID# will be assigned during the randomization process (see p. 3).
4. Each row represents one syringe dispensed.
5. Depending on randomization arm, not all cells in a row will need to be filled out (see sample examples).
6. It is acceptable to use “same” or “as above” if data in one cell is the same as the one above.
7. If participant has been starting on blinded EA (see p. 5), indicate the date in the heading, and then every cell in a row on and after that date will be filled out because the participant will be receiving both active drug and place

**Appendix 7: Educational Materials**

Nurse Enrollment Reminder sign

**
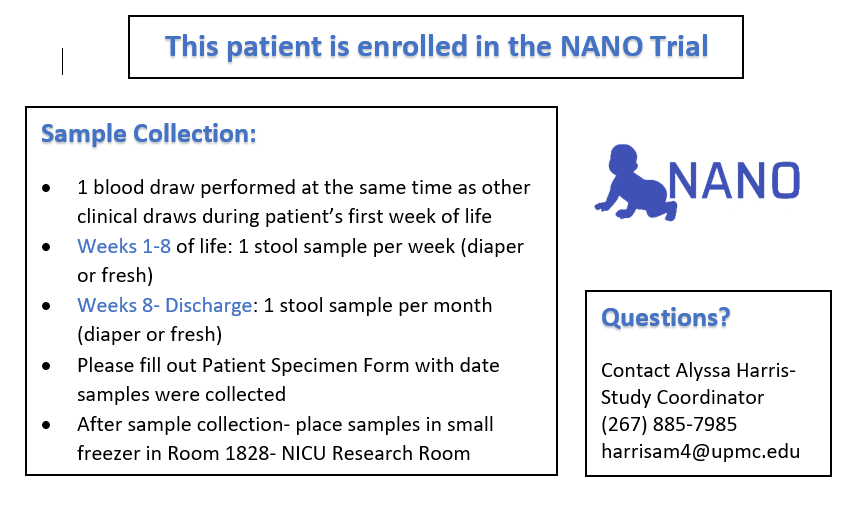
**

Investigator/Co-Investigator Inclusion/Exclusion criteria reminder

**FRONT**

**
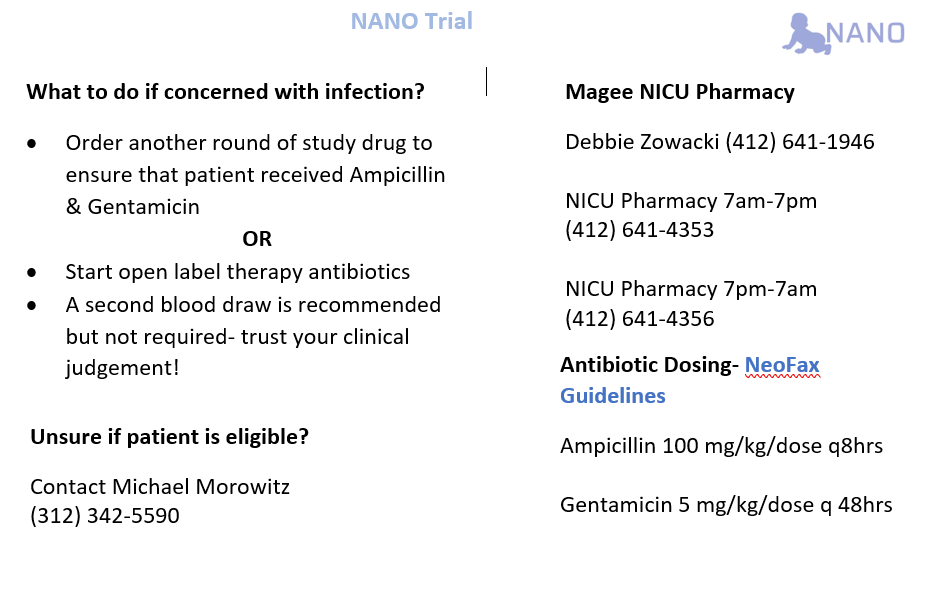
**

**BACK**

**
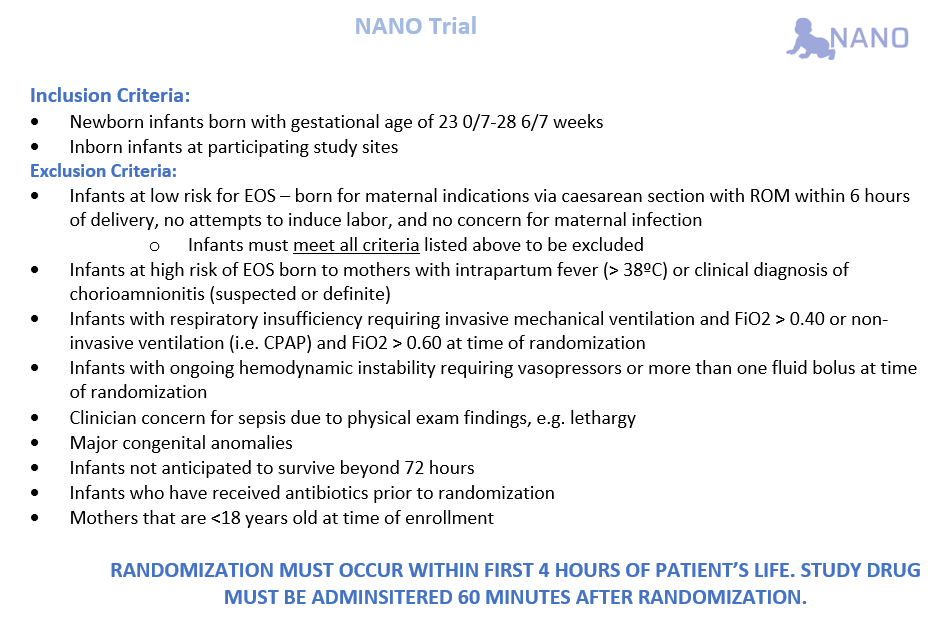
**


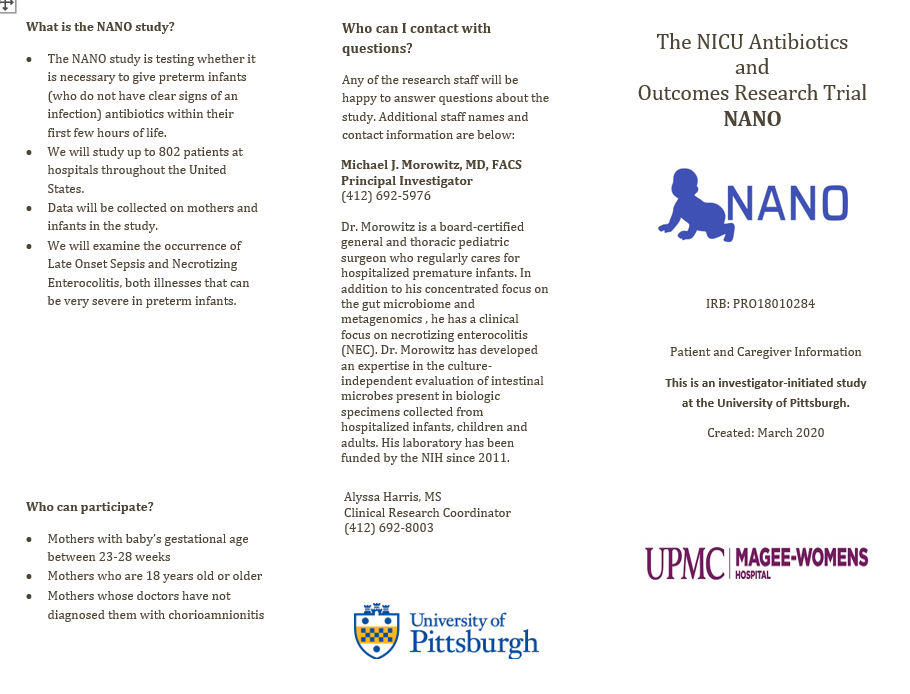
NANO Informational Pamphlet

**
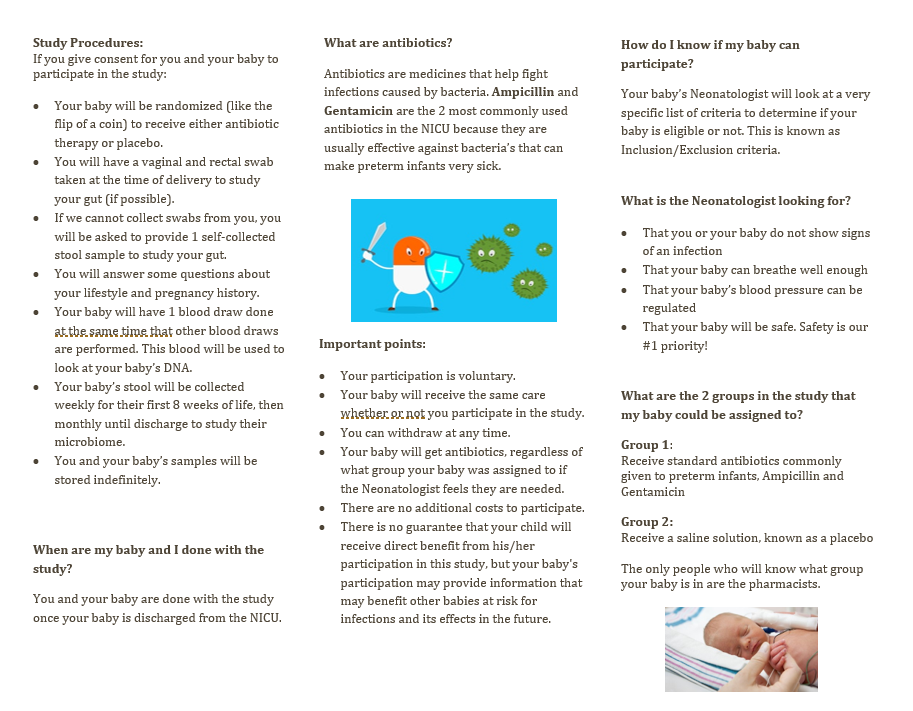
**

**Appendix 8: Co-Enrollment Instructions and Application**

INSTRUCTIONS FOR REQUEST FOR CO-ENROLLMENT APPLICATION FORM

REQUEST FOR CO-ENROLLMENT OF NANO STUDY SUBJECTS INTO ANOTHER CLINICAL TRIAL

In this document, **co-enrollment** is defined as enrolling NANO study subjects into another clinical trial at any time during the NANO study period up until discharge from the NICU or death. An **intervention** is any active process for which study consent is required, regardless of whether the intervention involves administration of pharmaceuticals.

NANO Investigators or collaborators seeking to co-enroll patients in the NANO trial studies must apply for permission to co-enroll. The NANO Steering Committee will review the application. If co-enrollment is approved, the Steering Committee will request DSMB approval.

The following should be provided using the Co-enrollment Application form:

I. Title of the study, name of the PI, study type (interventional vs. observational), and funding source.

II. Brief description of the study using the information below:

- Interventional Trials:
- Does the intervention have a potential mechanistic interaction with the NANO intervention?
- Will the intervention of proposed study have effects on the primary and secondary outcome variables of the NANO study?
- List the risks associated with the intervention.
- Provide plan on how to attribute overlapping safety concerns to separate trials.
- Interventional AND Observational Trials:
- Does the proposed trial include long term outcome assessments? If so, describe the timing and instruments to be used for long term assessments.
- What is maximum volume of blood to be drawn as part of the proposed study?
- How will informed consent burden be coordinated and minimized?

III. Items to Include with Interventional Study Submissions:

- A written confirmation from the non-NANO study PI of willingness to provide the unblinded treatment assignment to the NANO Steering Committee and DSMB for evaluation of adverse events in co-enrolled subjects.
- A written confirmation that the interventional study protocol does not contain administration of probiotics or prebiotics, or any other elements likely to interfere with gut bacterial colonization in newborns.
- Copy of the informed consent form

REQUEST FOR CO-ENROLLMENT APPLICATION FORM

*Please complete the following questions and submit this form to the* ***NANO Steering Committee***

**Study Title:**

**Principal Investigator:**

**Funding source:**

**Study Type *(check one)*:**  ❑ Intervention Trial ❑ Observational Study

**Did your IRB determine this study to be minimal risk or greater than minimal risk?**

❑ Minimal Risk ❑ Greater than Minimal Risk


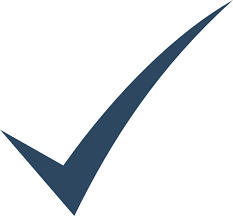
 Include the following with ***intervention*** trials:

- A written confirmation from the non-NANO study PI of willingness to provide the unblinded treatment assignment to the NANO Steering Committee and DSMB for evaluation of adverse events in co-enrolled subjects.
- A written confirmation that the interventional study protocol does not contain administration of probiotics or prebiotics, or any other elements likely to interfere with gut bacterial colonization in newborns.
- Copy of the informed consent form.

# Study Description:

*Answer the following questions in the sections below. Information can be provide as a separate document but should not exceed 3 pages.*

## Abstract Summary

Include the following information: background, objectives, intervention if any, risks, primary outcome, and study design.

## Coordination with NANO

1. Describe how informed consent will be obtained and how it will be coordinated with the NANO consent process.
2. What is the potential effect of the co-enrollment project on enrollment in NANO?
3. Describe plans for coordination of data collection between this study and NANO.
4. Are there any blood samples *(circle one)*? **Yes / No**
   - If yes, note the total volume of blood to be collected: ________
5. Address the following questions for **Intervention** trials:
   - Describe any potential interaction with the NANO intervention
   - Provide a plan for attributing overlapping safety concerns to separate trials.

**Appendix 9: ICF Template**

THE NICU ANTIBIOTICS AND OUTCOMES TRIAL (NANO)

This page summarizes key details about the NANO trial. We hope that this information will help you decide whether you would like to enroll yourself and your newborn baby in this study. More detailed information is provided in the rest of this document. If you have questions, please be sure to ask a member of the research team.

You are being asked whether you would like to enroll yourself and your newborn baby in a clinical trial, a type of research study. Being in a clinical trial may offer some benefits and/or pose some risks compared to regular medical care.

If you decide not to take part in the clinical trial, your baby will receive regular medical care for his/her hospitalization in the NICU at [INSERT LOCAL SITE NAME]. The standard of care for extremely low birthweight neonates is usually to receive antibiotics for 36-72 hours after delivery until the results from blood cultures that were drawn at the time of delivery are available.

The purpose of this clinical trial is to:

- determine if it is safe to **not** give newborn premature infants like yours (babies that do **not** have signs of infection) antibiotics in the first 48 hours of life.
- compare health outcomes (e.g., infections, poor growth, etc.) of infants who do receive antibiotics after delivery versus infants who do not receive antibiotics after delivery
- collect samples (infant stool and blood and maternal vaginal, rectal and stool) that will be used to study the relationship between antibiotics and the development of your baby’s gut microbiome (the types of bacteria, viruses and fungus that are present in your baby’s intestines).

If you decide to participate, your baby will be randomly assigned to receive 1-2 days of either antibiotics or a normal saline solution after he/she is admitted to the NICU. The nurses will collect weekly stool samples from your baby’s dirty diapers until eight weeks of life and then 1 sample will be collected monthly until your baby is discharged from the NICU. The nurses will also collect a sample of blood from your baby at the time of birth.

No researchers have ever conducted a study such as this. Therefore, we do not know if giving antibiotics increases or decreases the risks of health problems.

Possible risks of this study:

- If your baby is randomized to receive antibiotics, the possible risks are health problems that may be associated with giving antibiotics when an infection is not present. These include baby’s blood stream infections after 3 days of life, severe intestinal problems, serious long-term breathing problems and others that are listed on pages (7,8) of this consent.
- If your baby is randomized to receive a placebo (normal saline) instead of antibiotics, there is a risk if your baby has an infection in his/her bloodstream that was not expected. This risk would be a delay in antibiotic treatment for an infection that was not treated immediately after delivery, which may or may not increase the risks of complications of sepsis, which would include death and neurological impairment.
- If you choose not to enroll your baby to participate and your baby is given antibiotics as standard of care the possible risks are health problems that may be associated with giving antibiotics when an infection is not present. These include; blood stream infections after 3 days of life, severe intestinal problems, serious long-term breathing problems and others that are listed on pages (7,8) of this consent.

This research study may offer a potential benefit to infants randomized to the placebo group, as this group may have less health problems and infections than infants that receive antibiotics right after delivery; however, there is no guarantee that your child will benefit from participating this study. We hope that the information learned from this study will improve health outcomes in premature infants in the future.

If you decide not to participate in this research, your choices may include:

- Your baby receiving antibiotics if recommended by his/her doctor
- Taking part in another study
- You and your baby receive routine care for a premature delivery

CONSENT TO ACT AS A PARTICIPANT IN A RESEARCH STUDY

**STUDY TITLE**: THE NICU ANTIBIOTICS AND OUTCOMES TRIAL (NANO)

**Principal Investigator (PI):** [INSERT NAME, ADDRESS, PHONE (24 HOUR)]

**Co-Investigator (Co-I):** [INSERT NAME, ADDRESS, PHONE]

**Source of Support:** National Institute of Health (NIH)

You are being asked to take part in a research study and permit your baby to participate in this same research study for premature infants born between a gestational age (GA) of 23 weeks to less than or equal to 28 weeks. A member of the research team will explain what is involved in this study and how it will affect you and your baby. This consent form describes the study procedures, the risks and benefits of participation, as well as how you and your baby’s confidentiality will be maintained. Please take your time to ask questions and feel comfortable deciding whether to allow you and your baby to participate or not. This process is called informed consent. If you decide to allow yourself and your baby to participate in this study, you will be asked to sign this form. You will be given a copy of the form and should keep this copy for your records. It has information, including important names and telephone numbers, to which you may wish to refer in the future.

**WHY IS THIS STUDY BEING DONE?**

Historically, doctors have prescribed a few days of antibiotics to most premature babies, as a small percentage of premature babies are born with a bad infection called sepsis. If blood tests prove that there is no infection, then the antibiotics are stopped. However, approximately less than 2% of premature babies have this infection.It is possible that giving antibiotics increases the risk of infection and may worsen your baby’s health. It is also possible that giving antibiotics will improve your baby’s health. The purpose of this study is to help doctors decide whether antibiotics should be used in this situation.

**HOW MANY PEOPLE WILL TAKE PART IN THE STUDY?**

About 802 premature newborns and their mothers will be enrolled in this study from [INSERT LOCAL SITE NAME] Hospital and at least 6 other large hospitals within the United States. Approximately [INSERT LOCAL PROJECTED ENROLLMENT] mother and baby pairs will be enrolled from [INSERT LOCAL SITE NAME].

**WHAT PROCEDURES WILL BE PERFORMED FOR RESEARCH PURPOSES?**

By enrolling your baby in the study, you will also authorize (give permission to) this research team to access your medical record and obtain information regarding:

- Demographics
- Information about your pregnancy and delivery
- Antibiotics given to you before, during and after delivery
- Steroids given to you prior to your delivery
- Information about your placenta

This list is meant to serve as an example and additional questions may be asked.

This research study will involve the recording of current and/or future identifiable medical information from your baby’s hospital medical record including:

- Your baby’s medical record number and date of birth
- Antibiotics that your baby received during his/her NICU admission
- What types of feedings (your breast milk, donor breast milk or formula) and extra calories (fortification) that your baby was given in the hospital
- Any medical problems that your baby may have had while they were in the hospital
- The results of any tests or procedure that your baby received while in the NICU
- Your baby’s weekly length and weight measurements that are recorded in his/her medical record

This authorization is valid for an indefinite period of time; this identifiable medical record information may be made available to members of the research team for an indefinite period of time.

***Research Procedures:***

**Mother’s Procedures**:

1. When you deliver your baby, the doctor may obtain a vaginal sample from you at the time of delivery. A sterile swab (q-tip) will be gently inserted into the lower part of the vagina. The doctor will insert this swab without using a speculum (medical instrument that can be used for a vaginal exam). The swab will be rotated for about 10 seconds and then removed. We will analyze the germs (bacteria, viruses and fungii) present within this sample and compare it with the germs (bacteria, viruses and fungii) found in your baby stool samples.
2. When you deliver your baby, the doctor may obtain a rectal sample from you at the time of delivery. A sterile swab (q-tip) will be gently inserted to a depth of 4cm into the rectum. The swab will be rotated 3 times and then removed. We will analyze the germs (bacteria, viruses and fungi) present within this sample to help us understand your microbiome (genetic material that lives on and inside your body).
3. If you and your baby are enrolled after you deliver your doctors will not collect a vaginal or rectal sample.
4. You may be asked to collect a maternal stool sample within the first week after you deliver to provide to the study team. We will provide you with a hospital toilet liner, a pair of gloves, a wooden stick and a container to collect your stool sample. You can give the sample to the nurse taking care of your baby. This sample will be de-identified and put in the freezer in the research room.
5. We would like you to answer some questions about you and your general health. These questions will include questions about your diet, medications you were taking during your pregnancy, your education and ethnicity. If you chose not to answer these questions because you are uncomfortable, you and your baby can still take part in this study.

**For your baby:**

1. Randomization: One group of infants in this study will receive standard antibiotic therapy (ampicillin and gentamicin). The other group will receive an inactive substance (normal saline) instead of antibiotics. This normal saline is also called a placebo. Your baby will be randomly assigned to one of these two research groups. This means that your baby will have a 50/50 chance (like flipping a coin) of being assigned to either the antibiotic group or the placebo group. Neither the researchers or you will be able to choose what group your baby is in.

If you deliver more than one baby and they are each enrolled in this study, each of the babies that you deliver will be randomized to the same study treatment group.

1. Blinding: Neither you, your baby’s doctor, or the people taking care of your baby will know if your baby is receiving antibiotics or placebo (normal saline). This is called “blinding”. It is done to make sure that the observations and results are not affected by bias when the treatment group is known.

Your baby will only be permitted to participate if his/her doctor believes there are no signs of an infection. After your baby is born, the doctors taking care of your baby will closely monitor his/her vital signs (heart rate, breathing and temperature) and laboratory results. During this time, if your baby’s doctors identify signs of an infection, then your baby will receive antibiotics regardless of whether antibiotics or placebo were given to your baby in the first 2 days. Sample collection will continue.

**Participation in this trial will in no way affect any other part of your baby’s care.**

1. Stool collection: Spontaneously passed stool (feces) samples will be collected by your baby’s nurses from his/her dirty diapers. One to two samples will be collected for the first eight weeks after your baby is born and then once a month until your baby’s discharge from the NICU. These samples will be used to study the germs in your baby’s stool samples, how they change over time and to identify chemicals produced by the bacteria and viruses within the stool. The samples will be labeled only with a code number so that nobody outside of the [INSERT LOCAL SITE NAME] will be able to link a sample to your baby’s name. The samples may be sent outside of [INSERT LOCAL SITE NAME] for analysis but nobody outside of the [INSERT LOCAL SITE NAME] study team will be able to link samples to your baby’s name.
2. Blood draw: A blood draw of 0.3 to 0.4 mL will be taken to analyze your baby’s genes. will be taken.The blood draw will coordinate with clinical blood draws so as to minimize the number of times an indwelling line is accessed or to reduce the possibility of bruising by performing the heelstick After the sample is collected, it will be frozen for shipment. The samples will be labeled only with a code number so that nobody outside of the [INSERT LOCAL NAME] will be able to link a sample to your baby’s name. The samples may be sent outside of the [INSERT LOCAL SITE NAME] for analysis but nobody outside of the [INSERT LOCAL SITE TEAM] study team will be able to link samples to your baby’s name.
3. If your baby is transferred to [INSERT LOCAL SITE NAME], the nurses there will continue to collect stool samples as described above until your baby’s discharge. We will continue to review your baby’s medical record as described above while they are at [INSERT LOCAL SITE NAME].

In addition to several different types of bacteria, your baby’s stool samples will also contain some genetic material, proteins and other compounds (metabolites) that are produced by his/her body. We will study what is in your baby’s intestinal tract and how it relates to the genetic material and proteins that are produced by the bacteria. As part of this research, we may send genetic material and/or a sample of your baby’s stool to researchers at other institutions. There is a possibility that future genetic testing may include whole genome sequencing (WGS). These researchers will not receive any information about your baby, and the samples that we send to these researchers will be de-identified, which means they will have no way of knowing which samples came from which babies.

You will not be informed of the genetic information that may be received from your samples.

**HOW LONG WILL I BE IN THE STUDY?**

If you provide consent to participate in this study, you and/or your baby will remain in this study until your baby is discharged from [INSERT LOCAL SITE NAME]. You will not be asked to bring your baby in for any follow-up visits or exams for this study.

**WHAT ARE THE RISKS OF THE STUDY?**

- Randomization – Your baby will be assigned to a study group shortly after delivery (to receive antibiotics or not receive antibiotics). Although giving antibiotics is standard of care for most premature babies, it is possible that giving antibiotics increases the risk of infection and may worsen your baby’s health. It is also possible that giving antibiotics will improve your baby’s health. Finally, it is also possible that giving antibiotics makes no difference at all. The purpose of this study is to help doctors decide whether antibiotics should be used in this situation.
- Placebo – If your baby is assigned to the placebo group, your baby will receive normal saline instead of antibiotics. Normal saline is made up of water and salt and has no active substances. Normal saline is commonly given to infants to treat dehydration and has no known side effects.
- Ampicillin and gentamicin are usually given as standard of care. Since your baby may receive these antibiotics through randomization for this study, the risks of their administration may include;

skin rashes, hives, mild gastrointestinal upset with changes in stool. Temporary changes in kidney function that regulate electrolytes (sodium, calcium and magnesium) that go away after the drugs have been stopped and rarely hearing losses. These side effects are usually seen when high doses of the antibiotics have been ordered or the drugs are given for an extended period of time.

- Confidentiality – There is also a potential risk of loss of privacy when involved in a research study. The study staff will make every effort to keep you and your baby’s information private. All samples obtained from you and/or your baby will be identified with a study number, not names or medical record numbers. The code that links this number with you and/or your baby will remain in the research office at [INSERT LOCAL SITE NAME] in a password protected electronic file accessible to only the research coordinator or a person chosen the by the PI or Co-I.
- If we would use any of the stored samples for genetic research, the samples would be de-identified and no information would be put into your baby’s medical record.
- **Genetic Testing –** The risks associated with gene studies include the potential for a breach of confidentiality which could affect future insurability, employability, or reproduction plans, or have a negative impact on family relationships and/or result in paternity suits or stigmatization.
- **To further safeguard your privacy, genetic information that may be obtained in this study will not be placed in your baby's medical record**. Therefore, this study will not affect your baby’s future employment or his/her health insurance coverage. A Federal law, called the Genetic Information Nondiscrimination Act (GINA), generally makes it illegal for health insurance companies and group health plans to use genetic information in making decisions regarding eligibility or premiums. GINA also makes it illegal for employers with 15 or more employees to use your genetic information when making decisions regarding hiring, promoting, firing, or setting the terms of employment. This new Federal law does not protect you against genetic discrimination by companies that sell life, disability, or long-term care insurance.

**ARE THERE ANY BENEFITS TO TAKING PART IN THE STUDY?**

This research study may offer a potential benefit toinfants randomized to the placebo group, as this group may have less health problems and infections than infants that receive antibiotics right after delivery; however, there is no guarantee that your child will benefit from participating in this study . We hope that the information learned from this study will improve health outcomes inpremature infants in the future.

**ADVANTAGES** AND **DISADVANTAGES** OF PARTICIPATION IN THE NANO TRIAL

| **Treatment Arm 1: Assigned to receive antibiotics for at least 2 days after delivery** | | |
| --- | --- | --- |
| Possible advantages | - We estimate that 1 or 2 of every 100 study participants will be born WITH a blood stream infection that was not immediately recognized. These 1 or 2 babies will receive earlier antibiotic treatment if they are in this treatment group. It is possible that this earlier treatment is beneficial in babies born without signs of infection. | |
| Possible disadvantages | - Studies suggest that the following major medical problems may occur in premature infants that receive antibiotics at birth when they do not demonstrate signs of a blood stream infection: | |
|  | - Blood stream infections after 3 days of life - Side effects from the antibiotics (ampicillin and gentamicin) that include; skin rashes, hives, mild gastrointestinal upset with changes in stool, temporary changes in kidney function and rarely hearing losses. - Severe intestinal problems (e.g. Necrotizing enterocolitis) - Serious long-term breathing problems - Serious long-term eye problems - Abnormal brain development - Death during the NICU hospitalization | |
|  |  |  |
| **Treatment Arm 2: Assigned to receive saline solution (placebo) for 2 days after delivery** | | |
| Possible advantages | - If our research finds that the above medical problems are more common for infants that receive antibiotics at birth, then infants assigned to this treatment group can be expected to have fewer major medical problems. - Exposure to antibiotics can promote the growth of harmful bacteria that are more difficult to kill with antibiotics. This is called antibiotic resistance. Infants assigned to this treatment group may have less types of bacteria in their gut or lungs that are antibiotic resistant. - Studies suggest that infants who are born without a blood stream infection and do not receive antibiotics may grow better | |
| Possible disadvantages | - The risks of receiving placebo rather than antibiotics have not previously been studied and therefore unproven. We estimate that about 1 or 2 of every 100 study participants will be born WITH a blood stream infection that is not immediately known. It is possible that babies randomized to placebo could suffer from a delay in starting antibiotic coverage if they in are one of the babies that have an infection not immediately known in the first few hours of life. Such a delay may or may not increase the risks of complications of infection, which may include death or neurologic impairment. If your baby would be randomized to placebo, they will not receive antibiotics until laboratory results or the baby’s clinical condition suggests that an infection might be present. | |

**RESEARCH RELATED INJURY**

INSERT LOCAL COMPENSATION FOR INJURY LANGUAGE.

**WHAT ARE THE COSTS?**

There will be no costs to you, your family, or your insurance carrier resulting from participation in this study.

Usual medical care costs include all services that are considered medically necessary for your baby’s care during his/her hospitalization. The cost of this usual, ongoing medical care will be the responsibility of you or your insurance and may include deductibles and co-payments. Similarly, this care will be subject to all the same requirements and restrictions of your insurance.

**WILL I BE PAID FOR MY PARTICIPATION?**

There is no reimbursement (payment) for participating in this study.

You/your baby’s information and specimens used in this research study may contribute to a new discovery or treatment.  In some instances, these discoveries or treatments may be of commercial value and may be sold, patented, or licensed by the investigators and the [INSERT LOCAL SITE NAME] for use in other research or the development of new products.  You will not retain any property rights, nor will you share in any money that the investigators, the [INSERT LOCAL SITE NAME], or their agents may realize.

**WHAT ABOUT CONFIDENTIALITY?**

Study records that identify you and your baby will be kept confidential. Paper records and electronic records with confidential information will be stored in locked rooms and electronic records will be password protected. These records will be accessible only by study representatives.

A notation that you are taking part in this research study may be made in your electronic medical record. A copy of this signed consent form will also be added your/your child’s medical record information. Information from the research that relates to your general medical care may be included in the record (for example, list of allergies, results of standard blood tests done at the hospital labs).

The data collected in this study will be used for the purpose described in the form. By signing this form, you are allowing the research team access to your baby’s medical records and your medical records, which include Protected Health Information. Protected Health Information (PHI) consists of any health information that is collected about you or your baby, which could include your medical history or your baby’s medical history. The research team includes the individuals listed on this consent form and other personnel involved in this study at [INSERT LOCAL SITE NAME].

In addition to the investigators listed on the first page of this authorization (consent) form and their research staff, the following individuals will or may have access to identifiable information (which may include your identifiable medical information) related to you and your baby’s participation in this research study:

- Authorized representatives of the study sponsor (NIH) and the University of Pittsburgh Office of Research Protections may review your identifiable research information (which may include your identifiable medical information) for the purpose of monitoring the appropriate conduct of this research study.
- The de-identified research information that is obtained about you and your baby may be shared with investigators that are participating in this study from other hospitals.
- [INSERT ADDITIONAL LOCAL ENTITIES THAT MAY HAVE ACCESS TO PHI]

We will protect the privacy and confidentiality of your and your baby’s records, as described in this document, but cannot guarantee the confidentiality of the research records, including information obtained from the medical record, once your and your baby’s personal information is disclosed to others outside of [INSERT LOCAL SITE NAME].

To help us protect your privacy, we have a Certificate of Confidentiality from the National Institutes of Health. The researchers can use this Certificate to legally refuse to disclose information that may identify you in any federal, state, or local civil, criminal, administrative, legislative, or other proceedings, for example, if there is a court subpoena. The researchers will use the Certificate to resist any demands for information that would identify you, except as explained below.

The Certificate cannot be used to resist a demand for information from personnel of the United States Government that is used for auditing or evaluation of federally‐funded projects or for information that must be disclosed to meet the requirements of the federal Food and Drug Administration (FDA).

You should understand that a Certificate of Confidentiality does not prevent you or a member of your family from voluntarily releasing information about yourself or your involvement in this research. If an insurer, employer, or other person obtains your written consent to receive research information, then the researchers may not use the Certificate to withhold that information.

The Certificate of Confidentiality will not be used to prevent disclosure to state or local authorities to prevent serious harm to yourself, children or others, for example in cases of baby abuse or neglect. If the researchers learn that you or someone with whom you are involved is in serious danger of harm, they will need to inform the appropriate agencies as required by [INSERT LOCAL STATE] law.

The PI is not required to release to you research information that is not part of your baby’s medical record. The information and stool samples will be kept indefinitely. This is because information that is collected for research purposes continues to be used and analyzed for many years and it is not possible to determine when this will be complete. The data, samples and genetic data generated from samples may be shared with other researchers and with federal repositories, in a de-identified manner (without identifiers).

Information from this study may be used in medical publications or presentations or may be deposited into publicly accessible databases of genetic information. Your name and your baby’s name and other identifying information will be removed before this information is used.

A description of this clinical research study is available on <http://www.ClinicalTrials.gov>, as required by U.S. law. This web site will not include information that can identify you. At most, the web site will include a summary of the results. You can search the web site at any time.

**WHAT ARE MY RIGHTS AS A PARTICIPANT?**

Taking part in this study is voluntary. If you choose not to participate in this study, your care and your baby’s care at the [INSERT LOCAL SITE NAME] will not be affected. You may withdraw, at any time, consent for you and your baby’s participation in this research study, including your authorization to allow the research team to review your/your baby’s medical records. If you do so, you and your baby will no longer be permitted to participate in this study. Any information obtained from you/your baby up to that point will continue to be used by the research team. Leaving the study will not affect your care at the [INSERT LOCAL SITE NAME].

If you choose for you and your baby to no longer be in the study, and you do not want any of your future health information to be used, you must inform the PI in writing at the address on the first page.

**FOR HOW LONG WILL THE INVESTIGATORS BE PERMITTED TO USE AND DISCLOSE IDENTIFIABLE INFORMATION RELATED TO THIS RESEARCH STUDY?**

[INSERT LOCAL DATA RENTENTION LANGUAGE]

**VOLUNTARY CONSENT/ PARENTAL PERMISSION**
The above information has been explained to me and all of my current questions have been answered. I understand that I am encouraged to ask questions about any aspect of this research study during the course of this study, and that such future questions will be answered by a qualified individual or by the investigator(s) listed on the first page of this consent document at the telephone number(s) given. I understand that I may always request that my questions, concerns or complaints be addressed by a listed investigator.

I understand that I may contact the Human Subjects Protection Advocate of the IRB Office, University of Pittsburgh (1-866-212-2668) to discuss problems, concerns, and questions; obtain information; offer input; or discuss situations in the event that the research team is unavailable.

By signing this form, I agree for me and my baby to participate in this research study and authorize the use of my and my baby's medical record information for the purpose described above. A copy of this consent form will be given to me/my baby.

I understand that, as a minor (age less than 18 years), my baby is not permitted to participate in this research study without my consent. Therefore, by signing this form, I give my consent for his/her participation in this research
study.

___________________________________________

Printed Name of Mother-Subject Signature of Mother

_________________________________ ___________________________________________

Printed Name of Baby-Subject Date/Time

**CERTIFICATION of INFORMED CONSENT**
I certify that I have explained the nature and purpose of this research study to the above-named individual(s), and I have discussed the potential benefits and possible risks of study participation. Any questions the individual(s) have about this study have been answered, and we will always be available to address future questions as they arise. I further certify that no research component of this protocol was begun until after this consent form was signed.

Printed Name of Person Obtaining Consent Role in Research Study

Signature of Person Obtaining Consent Date/Time

**Appendix 10: NANO Event Table**

|  | **In NICU/Hospital** | | | | | | |  |
| --- | --- | --- | --- | --- | --- | --- | --- | --- |
|  | **Pre-Randomization** | **Delivery** | **Randomization**  **(up to 4 hours after birth)**** | **Post- Randomization**  **(60 mins after randomization)**** | **Week 1** | **Weeks 2-8** | **Months 3-8** | **Discharge from NICU** |
| **Enrollment** |  |  |  |  |  |  |  |  |
| Maternal Eligibility Screen^a^ | X | X | X |  |  |  |  |  |
| Informed Consent | X |  |  |  |  |  |  |  |
| Infant Eligibility Screen^b^ |  | X |  |  |  |  |  |  |
| Randomization^c^ |  |  | X |  |  |  |  |  |
| **Intervention** |  |  |  |  |  |  |  |  |
| Study Drug Administration^d^ |  |  |  | X | X |  |  |  |
| **Data Collection** |  |  |  |  |  |  |  |  |
| Maternal PHI | X |  |  |  | X |  |  |  |
| Maternal Demographics | X |  |  |  | X |  |  |  |
| Maternal Antibiotic Exposure | X |  |  |  | X |  |  |  |
| Pregnancy | X |  |  |  | X |  |  |  |
| Infant Baseline |  |  |  | X |  |  |  |  |
| Infant Rule-Out |  |  |  |  | X^e^ |  |  |  |
| Infant Weight & Length |  |  |  |  | X | X | X |  |
| Infant Nutrition |  |  |  |  | X^f^ | X | X |  |
| Infant Antibiotic Administration^g^ |  |  |  |  | X | X | X |  |
| Discharge |  |  |  |  |  |  |  | X |
| **Sample Collection** |  |  |  |  |  |  |  |  |
| Maternal Vaginal & Rectal swabs |  | X^h^ |  |  |  |  |  |  |
| Maternal Stool |  |  |  |  | X |  |  |  |
| Infant Blood |  |  |  |  | X |  |  |  |
| Infant Stool^i^ |  |  |  |  | X | X | X |  |
| **Reporting** |  |  |  |  |  |  |  |  |
| Adverse Events^j^ |  |  |  | X | X | X | X | X |

^a^ Maternal Eligibility will be monitored up until delivery.

^b^ Research staff will ensure eligibility by going through the Inclusion/Exclusion criteria with treating Neonatologist.

^c^ Randomization must occur within 4 hours following birth. If randomization does not occur in this 4 hour window, the baby is no longer eligible.

^d^ EA administration will consist of IV administration of Ampicillin/Gentamicin or placebo at site approved dosing guidelines. EA administration must occur 30-45 minutes following randomization.

^e^ Sepsis rule out will be based on blood tests drawn at birth. This rule out period will follow local site guidelines.

^f^ Infant nutrition should be collected on Days 3, 7, 14, 28 and 60.

^g^ Documentation of antibiotic administration should be for ALL antibiotics administered outside of study drug. This will be marked as a protocol deviation.

^h^ Maternal vaginal & rectal swab collection will not be feasible at certain sites.

^i^ Infant stool samples should be randomly expelled (fresh or from diaper). 1 stool sample per week for the first 8 weeks will be collected. Following 8 weeks, 1 stool sample per month will be collected.

^j^ All SAEs or events that are unexpected and thought to be related to lack of antibiotic administration/placebo will be reported to the CCC PI (Morowitz) within 72 hours of the site becoming aware of the event. Full documentation of the event is required within 7 days of becoming aware of the event.

** If time limits are exceeded, the baby is no longer eligible for the trial

**Appendix 11: Protocol Deviation Log**

| **Subject ID#** | **Date of Event** | **Date PI was notified of event** | **Brief Description of Event** | **Does the event represent non-compliance that must be reported to the IRB^[[1]](#footnote-2)^ ? If yes, list date.** | **Was the event reported to the sponsor, DSMB or an external group? If yes, list group and report date** | **Provide a Corrective Action to Prevent Future Occurrence of the Event as applicable.** | **PIs Initials and date** |
| --- | --- | --- | --- | --- | --- | --- | --- |
|  |  |  |  | Yes  No  Date: |  |  | Date: |
|  |  |  |  | Yes  No  Date: |  |  | Date: |
|  |  |  |  | Yes  No  Date: |  |  | Date: |
|  |  |  |  | Yes  No  Date: |  |  | Date: |

**Appendix 12: AE/SAE Event Table**

## Adverse and Serious Adverse Events

**Expected Adverse Events** –reported in DSMB analyses

The following listed adverse events are expected and will be recorded in the electronic database. The DCC will track and report all adverse events and report to the DSMB every 3 months. The DSMB will report to the Investigators regarding the risks of the study. Safety reports will be provided to all sites for reporting to their local IRBs.

1. IVH grade 1-2 -Within the first 10 days of life
2. PVL- Head ultrasound findings until discharge
3. ROP (retinopathy of prematurity) -Until discharge

**Serious Adverse Events**

The following SAEs will be reported within 24-48 hours of the PI learning of the event. All SAEs will be reported in the electronic database. Events will be flagged and the DCC’s PI and Study Coordinator will receive an automatically generated alert of the event. The DCC will forward (via email) all such events to the IDSMB and IRB for review. Documentation of the SAE should include de-identified source documents along with the corresponding de-identified patient summary.

1. Death -Until discharge
2. Severe IVH – grades III, IV -Head ultrasound findings until discharge
3. Sepsis (early and late onset) – positive microbiology culture and decision by treating team to treat with a course of intravenous antibiotics
4. Spontaneous Intestinal Perforation or NEC requiring drain placement and/or laparotomy DOL 0-7
5. NEC (stage > 2) after DOL 7

**Unanticipated Problems**

UAPs should be sent to the DSMB within 24-48 hours of the DCC learning of the event. UAPs will be reported in the electronic database. Documentation of UAPs will include as much information as provided to the DCC PI.

Internal SAEs that are Unexpected, Related or Possibly related to the research intervention and places subjects or others at a greater risk of physical or psychological harm than was previously known or recognized must be reported to the DCC IRB as a UAP within 24 hours of the PI learning of the event.

External SAEs that are Unexpected, Related to the research intervention and suggest that the research places subjects or others at greater risk than was previously recognized and Related to the research intervention will be reported to the DCC IRB as a UAP within 30 working days of their receipt by the DCC PI.

All other reportable UAPs should be sent to the DCC IRB within 10 working days of the DCC PI becoming aware of the event.

1. Noncompliance that is reportable to the IRB is outlined in Chapter 17. This includes:

   Noncompliance that meets the definition of an unanticipated problem involving risks to human subjects or others in that it is related or possibly related to the research, is unexpected and places research subjects or others at greater risk of harm (physical, psychological, economic or social) than was previously known or recognized.

   Noncompliance that may significantly adversely affects the rights or welfare of participants, or significantly compromises the research data.

   In PittPRO, these submissions are titled Reportable New Information (RNI). In OSIRIS, these are titled Noncompliance or Unanticipated Problems Involving Risks to Human Subjects or Others. [↑](#footnote-ref-2)
